# Improved integration of single-cell transcriptome data demonstrates common and unique signatures of heart failure in mice and humans

**DOI:** 10.1093/gigascience/giae011

**Published:** 2024-04-04

**Authors:** Mariano Ruz Jurado, Lukas S Tombor, Mani Arsalan, Tomas Holubec, Fabian Emrich, Thomas Walther, Wesley Abplanalp, Ariane Fischer, Andreas M Zeiher, Marcel H Schulz, Stefanie Dimmeler, David John

**Affiliations:** Institute of Cardiovascular Regeneration, Goethe University Frankfurt, Theodor-Stern-Kai 7, 60590 Frankfurt am Main, Germany; German Centre for Cardiovascular Research (DZHK), 60590 Frankfurt am Main, Germany; Cardio-Pulmonary Institute (CPI), Goethe University Frankfurt, Theodor-Stern-Kai 7, 60590 Frankfurt am Main, Germany; Institute of Cardiovascular Regeneration, Goethe University Frankfurt, Theodor-Stern-Kai 7, 60590 Frankfurt am Main, Germany; German Centre for Cardiovascular Research (DZHK), 60590 Frankfurt am Main, Germany; Department of Cardiovascular Surgery, Goethe University Hospital, 60590 Frankfurt am Main, Germany; Department of Cardiovascular Surgery, Goethe University Hospital, 60590 Frankfurt am Main, Germany; Department of Cardiovascular Surgery, Goethe University Hospital, 60590 Frankfurt am Main, Germany; German Centre for Cardiovascular Research (DZHK), 60590 Frankfurt am Main, Germany; Cardio-Pulmonary Institute (CPI), Goethe University Frankfurt, Theodor-Stern-Kai 7, 60590 Frankfurt am Main, Germany; Department of Cardiovascular Surgery, Goethe University Hospital, 60590 Frankfurt am Main, Germany; Institute of Cardiovascular Regeneration, Goethe University Frankfurt, Theodor-Stern-Kai 7, 60590 Frankfurt am Main, Germany; German Centre for Cardiovascular Research (DZHK), 60590 Frankfurt am Main, Germany; Cardio-Pulmonary Institute (CPI), Goethe University Frankfurt, Theodor-Stern-Kai 7, 60590 Frankfurt am Main, Germany; Institute of Cardiovascular Regeneration, Goethe University Frankfurt, Theodor-Stern-Kai 7, 60590 Frankfurt am Main, Germany; Institute of Cardiovascular Regeneration, Goethe University Frankfurt, Theodor-Stern-Kai 7, 60590 Frankfurt am Main, Germany; German Centre for Cardiovascular Research (DZHK), 60590 Frankfurt am Main, Germany; Cardio-Pulmonary Institute (CPI), Goethe University Frankfurt, Theodor-Stern-Kai 7, 60590 Frankfurt am Main, Germany; Institute of Cardiovascular Regeneration, Goethe University Frankfurt, Theodor-Stern-Kai 7, 60590 Frankfurt am Main, Germany; German Centre for Cardiovascular Research (DZHK), 60590 Frankfurt am Main, Germany; Cardio-Pulmonary Institute (CPI), Goethe University Frankfurt, Theodor-Stern-Kai 7, 60590 Frankfurt am Main, Germany; Institute of Cardiovascular Regeneration, Goethe University Frankfurt, Theodor-Stern-Kai 7, 60590 Frankfurt am Main, Germany; German Centre for Cardiovascular Research (DZHK), 60590 Frankfurt am Main, Germany; Cardio-Pulmonary Institute (CPI), Goethe University Frankfurt, Theodor-Stern-Kai 7, 60590 Frankfurt am Main, Germany; Institute of Cardiovascular Regeneration, Goethe University Frankfurt, Theodor-Stern-Kai 7, 60590 Frankfurt am Main, Germany; German Centre for Cardiovascular Research (DZHK), 60590 Frankfurt am Main, Germany; Cardio-Pulmonary Institute (CPI), Goethe University Frankfurt, Theodor-Stern-Kai 7, 60590 Frankfurt am Main, Germany

**Keywords:** cross-species analysis, cardiovascular disease, heart failure with reduced ejection fraction, coronary artery ligation, single-cell integration, cross-species integration workflow

## Abstract

**Background:**

Cardiovascular research heavily relies on mouse (*Mus musculus*) models to study disease mechanisms and to test novel biomarkers and medications. Yet, applying these results to patients remains a major challenge and often results in noneffective drugs. Therefore, it is an open challenge of translational science to develop models with high similarities and predictive value. This requires a comparison of disease models in mice with diseased tissue derived from humans.

**Results:**

To compare the transcriptional signatures at single-cell resolution, we implemented an integration pipeline called *OrthoIntegrate*, which uniquely assigns orthologs and therewith merges single-cell RNA sequencing (scRNA-seq) RNA of different species. The pipeline has been designed to be as easy to use and is fully integrable in the standard Seurat workflow.

We applied *OrthoIntegrate* on scRNA-seq from cardiac tissue of heart failure patients with reduced ejection fraction (HFrEF) and scRNA-seq from the mice after chronic infarction, which is a commonly used mouse model to mimic HFrEF. We discovered shared and distinct regulatory pathways between human HFrEF patients and the corresponding mouse model. Overall, 54% of genes were commonly regulated, including major changes in cardiomyocyte energy metabolism. However, several regulatory pathways (e.g., angiogenesis) were specifically regulated in humans.

**Conclusions:**

The demonstration of unique pathways occurring in humans indicates limitations on the comparability between mice models and human HFrEF and shows that results from the mice model should be validated carefully. *OrthoIntegrate* is publicly accessible (https://github.com/MarianoRuzJurado/OrthoIntegrate) and can be used to integrate other large datasets to provide a general comparison of models with patient data.

## Introduction

Animal experiments are a powerful tool to improve our understanding of pathophysiological conditions and to predict responses to new therapeutic approaches [1]. However, due to ethical considerations, they are controversially discussed [2], and their predictive capacity for toxicity and drug responses is limited [3, 4]. Especially mice are commonly used to model human diseases as they are relatively inexpensive, have short generation times, and have large numbers of offspring. Additionally, mice have a relatively close physiological and phylogenetic relationship with humans [5, 6]. Mice protein-coding genes are on average 85% identical to humans [4], and over 90% of both genomes have regional conserved synteny [7]. Due to these advantageous breeding characteristics and their high sequencing conservation to humans, hundreds of different mouse models have been developed to study human diseases [8] like heart failure [9] or even diseases that do not occur naturally in mice like Alzheimer or Parkinson disease [10].

To study cardiovascular diseases, which remain the leading cause of morbidity and mortality in the aging society, the ligation of the left anterior descending coronary artery model (LAD) is often used to induce myocardial infarction, which results in ischemic heart failure with reduced ejection fraction (HFrEF) [11, 12]. Thereby, the LAD is ligated to mimic the clotted artery as it occurs after infarction. While short-term reperfusion then allows to mimic the reopening of the coronary artery by catheter-based interventions, often chronic ligation is used to induce heart failure over the course of >4 weeks. As this method describes a similar decline in heart function, scientists use LAD mouse models to simulate HFrEF and develop and test new therapeutic strategies [13–15]. Patients who have HFrEF are unable to pump sufficient amounts of blood to meet the demands of body organs [16].

To address the comparability of HFrEF in human to mouse models, we used single-nuclei RNA sequencing data, enabling us to assess transcriptional regulatory pathways in all cardiac cell populations with high resolution and accuracy [17, 18]. In order to analyze single-cell RNA sequencing (scRNA-seq) data from various samples, integration pipelines were developed to combine individual cells from different subjects into clusters with similar expression patterns [18, 19]. Yet these bioinformatic tools can only integrate datasets from identical species. Several studies developed algorithms to compare messenger RNA (mRNA) expression patterns across species [20–22]. However, a standardized and easy way to compare single-cell/nuclei RNA sequencing datasets of human and mouse by directly integrating the data is still missing [18, 23, 24]. Overcoming these limitations and the highly increasing demand for comparison of various organisms prompted us to develop a R package called *OrthoIntegrate*. It features a pipeline for integration of single-cell datasets and ortholog assignment, allowing the simple integration of data from animal models and human patients. For the ortholog assignment process, we implemented an algorithm in the workflow that adjusts the different nomenclature between species before the integration takes place, by using the databases of Ensembl, NCBI, and Uniprot [25–27]. Using our newly established pipeline, which is completely compatible with standard seurat workflows, we explored the gene expression patterns in mouse models of HFrEF compared to human samples. While 54% of genes were commonly regulated in both species, we also observed significant differences in differentially expressed genes and regulated pathways in patients with heart diseases compared with the corresponding mouse model.

## Results

### 1-to-1 ortholog assignments

To integrate single-cell data from different species, we established a table of gene names, which contains 1 human gene for each mouse gene, by which it will be replaced (1-to-1 orthologs). We performed the same approach for generating a table of gene names between human and zebrafish genes.

In order to generate these 1-to-1 orthologs, we utilized the Needleman–Wunsch algorithm [28] to perform a pairwise global alignment between possible orthologs retrieved by the Ensembl database. This calculation determines alignment scores based on differences in the amino acid or nucleotide sequences. In case no orthologs were found or a protein or nucleotide sequence was not available for a particular gene, a lowercase matching of the human gene was searched for in the mouse gene database (Supplementary Fig. S1A).

**Figure 1:**
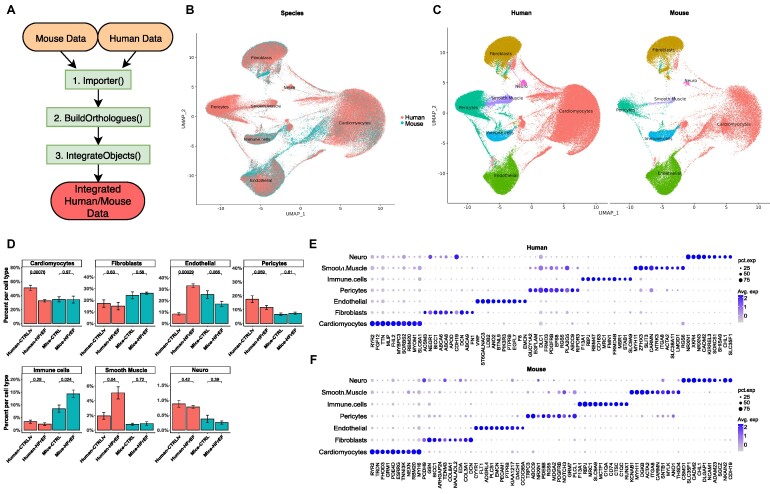
Integrated human and mouse snRNA-seq data of healthy and heart failure samples. (A) Use case diagram of *OrthoIntegrate*: shown are the steps that are run by the user within their standard Seurat workflow. First, the Import function is used to create Seurat objects from scRNA-seq data; second, orthologs are searched by the BuildOrtholog function, and the third step creates an integrated object with uniform nomenclature by using the IntegrateObjects. (B) UMAP showing human cells (red) and mice cells (blue) in a common UMAP projection. In addition, cell types for the cell clusters can be seen. (C) UMAP with defined clusters according to Seurat’s clustering, divided by species. Cells of mouse and human origin commingled in all clusters. There are no clusters formed that originated from only 1 of the 2 species. The cells were identified as cardiomyocytes (red), fibroblasts (yellow), endothelial cells (green), pericytes (turquoise), immune cells (blue), smooth muscle cells (purple), and neuronal cells (pink). (D) Bar plot showing cell composition of cell types in human (red) and mice (blue) samples. Samples were grouped based on their origin into human controls from the left ventricle (Human-CTRLlv), human HFrEF (Human-HFrEF), mouse controls (Mice-CTRL), and mouse HFrEF model (Mice-HFrEF). Cell types were then analyzed for their composition from the previously mentioned groups and plotted. *P* values above the certain groups were calculated by 2-sided Student’s *t*-test. (E) Dot plot depicting the average expression levels and expression proportions in human samples of the top 10 feature genes for the found cell types. The size of the dot represents the proportion of cells expressing the indicated gene within a cell type, and the color indicates the average expression level of cells. (F) Dot plot depicting the average expression levels and expression proportions in mice samples of the top 10 feature genes for the found cell types. Similar to (E), the size of the dot represents the proportion of cells expressing the indicated gene within a cell type, and the color indicates the average expression level of cells.

The Ensembl database assigned a total of 21,428 mouse orthologs to our human gene ID symbols. However, only 77% (16,573) of these were uniquely assigned. Through our *OrthoIntegrate* pipeline, we increased the number of assignments to 82% (17,504). Hereby, 714 mouse genes were assigned by protein sequence alignment, 89 through nucleotide sequence alignment, 42 by using the Levenshtein distance between gene names, and 86 using our lowercase matching approach. Most of the 86 matches found by lowercasing were long noncoding RNAs with identical gene names. We then proceeded by filtering the human and mice data by these orthologs in our pipeline and replaced the mice nomenclature by the human nomenclature for the corresponding samples (Supplementary Fig. S1B). In the end, we could assign ∼82% of the mice genes to human orthologs (Supplementary Table S2). Replacing mouse gene names with the human ortholog allowed us to integrate the human patient data with the mouse model data into 1 single-cell object (Fig. 1A). Moreover, we aimed to underscore the versatility of *OrthoIntegrate*. Therefore, we integrated and clustered scRNA-seq datasets related to Alzheimer disease from human, mouse, and zebrafish with the *OrthoIntegrate* pipeline (Supplementary Fig. S6). We successfully created clusters representing excitatory and inhibitory neurons, as well as astrocytes, in the 3 species (Supplementary Fig. S6A, B). Given the focus and the size of the human study and data, most of the excitatory neurons found were of human origin, but we showed that excitatory neurons found in mice were also assigned to the same clusters and showed comparable marker genes (Supplementary Fig. S6A–D). Similar results were obtained for inhibitory neurons and astrocytes, proving a successful integration of all 3 datasets (Supplementary Fig. S6C–F).

### Cell-type composition in human and mouse upon HFrEF

After demonstrating the practicality of the integrated dataset, the biological differences of the human mouse datasets were analyzed. The absence of species-specific clusters in the combined Uniform Manifold Approximation and Projection (UMAP) plot confirms that human and mouse hearts comprise similar cell types and gene expression patterns (Fig. 1B). This is additionally verified by similar cell type–specific marker genes in both species in the different cell clusters (Fig. 1E, F). The specific marker genes allowed the annotation of the clusters into cardiomyocytes (CMs), pericytes (PCs), smooth muscle cells (SMCs), fibroblasts (FBs), endothelial cells (ECs), immune cells (ICs), and neuronal cells (NCs) (Fig. 1C). In addition, we analyzed how the distribution of cell types was affected by the heart failure phenotype. Thereby, a 20% decrease in human CMs was observed when comparing the control samples with the HFrEF samples (45% → 25%) (Fig. 1D). However, in mice, there was no difference in the numbers of CMs between the infarcted and control mice (both comprise about ∼25% CMs) (Fig. 1D). Furthermore, we found differences in the distribution of ECs in the human versus mouse samples. Specifically, we observed a significant increase in the EC population in samples from HFrEF patients (∼30%) compared to healthy hearts (∼8%). In contrast, we noticed decreased EC numbers in mice upon infarction (from 25% in controls to 18% after chronic infarction). Minor changes were also observed in the contributions of other cell types (Fig. 1D).

### Comparison to other integration methods

**Figure 2:**
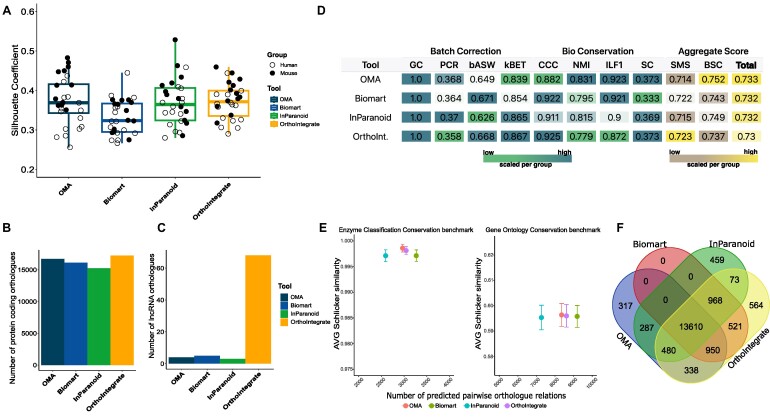
Comparison of snRNA-seq data integration with orthologs from *OrthoIntegrate* and other ortholog databases. (A) Box plot showing the average silhouette coefficient for clusterings based on different databases and tools. The dark blue box stands for the silhouette coefficient of the clustering made with an orthologous list using the tool OMA (orthologous matrix). It is followed by the results for biomaRt (light blue), InParanoid (green), and the pipeline *OrthoIntegrate* (yellow). On the y-axis, you can see the value of the silhouette coefficient. Additionally, each silhouette coefficient was calculated for each sample and depicted as a circle in their species-specific color. (B) Bar plot with number of orthologs found that codes for a protein (C) and bar plot with number of orthologs found that codes for lncRNA. On the x-axis, the used tool is depicted. (D) Table showing results of different metric calculations to comprehend batch correction and biological conservation of clusterings based on orthologous lists of OMA, biomaRt, InParanoid, and *OrthoIntegrate* (bASW: batch average silhouette width; BCS: bioconservation score; CCC: cell cycle conservation; GC: graph connectivity; ILF1: isolated labels F1 score; NMI: normalized mutual information; PCR: principal component regression comparison; SC: silhouette coefficient; SMS: species mixing score). The color code represents low and high values and is scaled per column (low = green, brown; high = blue, yellow). (E) Schlicker similarity scores calculated for OMA (red), Biomart (green), InParanoid (blue), and *OrthoIntegrate* (purple) in terms of enzyme classification conservation (left) and gene ontology conservation (right). (F) Venn diagram highlighting the numbers of uniquely found orthologs between human and mouse per tool and their overlap between each other (blue = OMA, red = biomart, green = biomart, yellow = *OrthoIntegrate*).

We carefully inspected our data to determine species-specific distribution by creating UMAP plots of all cells in our integrated object. Figure 1B shows that cells of mouse and human origin commingled in all clusters, which indicates a successful integration based on the cell types and not on the species. We additionally compared our *OrthoIntegrate* pipeline to other ortholog databases and tools to assess the advantages of our ortholog assignments. For this purpose, we created the same scRNA-SEQ datasets using the different ortholog lists OMA, Biomart, and InParanoid [29–31]. Visualization of the integration by UMAP plots shows an integration of human- and mouse-derived cells in the individual cell clusters also with the alternative orthologous list (Supplementary Fig. S2A–C). However, besides the visual impression, quantitative metrics were used to assess the quality of the clustering, and we calculated silhouette coefficients, which measure the quality of the clustering independent from the number of clusters. Integration by *OrthoIntegrate* resulted in the highest silhouette coefficients compared to the other ortholog databases, suggesting an improved clustering (Fig. 2A). Additionally, it is noteworthy to mention that our pipeline achieved by far the most 1:1 protein coding and lncRNA coding orthologous pairs in comparison to the other described methods (Fig. 2B, C). To further determine the clustering quality after integration, we computed supplementary metrics recommended by the single-cell integration benchmark scib package [32] and the Orthology Benchmark Service. We also calculated the species mixing score and bioconservation score, following the guidelines of the BENGAL pipeline (Fig. 2D) [32, 33]. Remarkably, our method not only achieved the highest number of uniquely mapped orthologous pairs but also demonstrated high performance across individual metrics in comparison with alternative tools (Fig. 2D–F).

### Differential gene expression between mice and humans

The differentially expressed gene (DEG) analysis showed strong similarities in the regulated genes upon HFrEF. However, some genes showed differences in their expression patterns, mainly when the cell types were analyzed individually. Overall, we found a comparable number of DEGs in both species (4,141 in humans, 4,654 in mice).

**Figure 3:**
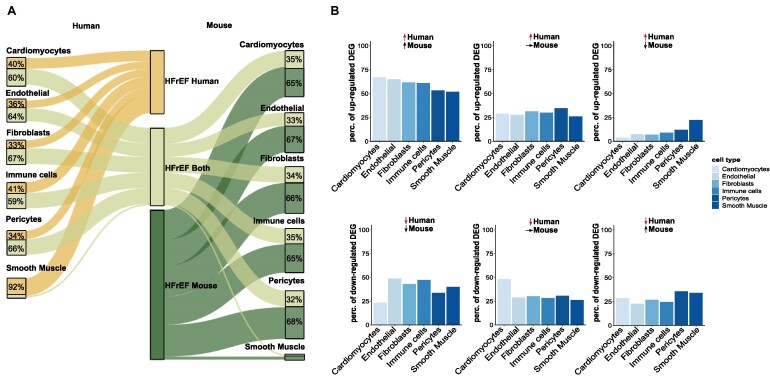
Similarities and differences revealed by DEG analysis. (A) Sankey plot illustrating the distribution of DEGs in the corresponding cell types. The width of the paths illustrates the number of DEGs that are human specific (yellow), detected in both species (light green), or mouse specific (dark green). DEG analysis was performed for each cell type individually. Neuronal cells were omitted from all further analyses due to their insufficient number of cells in the mouse data. (B) Bar graph of upregulated (top) and downregulated (bottom) genes in humans, along with the expression in mice. The panels show genes that are either commonly regulated (left), regulated in humans, and not regulated in mice (middle) and regulated in opposite directions.

The average of commonly regulated genes per cell type (Fig. 3A, left side) showed that around 54% of DEGs found in humans were also regulated in mice, with minor differences between cell types. Upregulated genes showed a generally higher comparability compared to downregulated genes (Fig. 3B). Only in smooth muscle cells were many more human-specific DEGs regulated in opposite directions (Fig. 3B, right upper panel). Averaging the mouse regulated DEGs (Fig. 3A, right side) showed that only about 34% of the cell type–specific DEGs in mice were regulated in humans, indicating a more substantial transcriptional effect of the LAD model compared to the human disease.

Figure 3B separately shows the upregulated (top panel) and downregulated (lower panel) genes in humans and their regulation in mice. For the upregulated genes in humans, around 50–70% of the corresponding mouse genes were also upregulated, around 25% were not regulated, and only about 5–20% were regulated in the opposite direction, suggesting that overall activation occurs mainly in similar expression pathways across all cell types. In the downregulated genes in humans, we observed a strikingly low number of commonly regulated genes in cardiomyocytes. There, only 23.3% of the downregulated genes were also decreased in mice. Most of them were either not regulated (48.2%) or even upregulated in mice (28.5%). The other cell types show a higher percentage of commonly downregulated genes.

**Figure 4:**
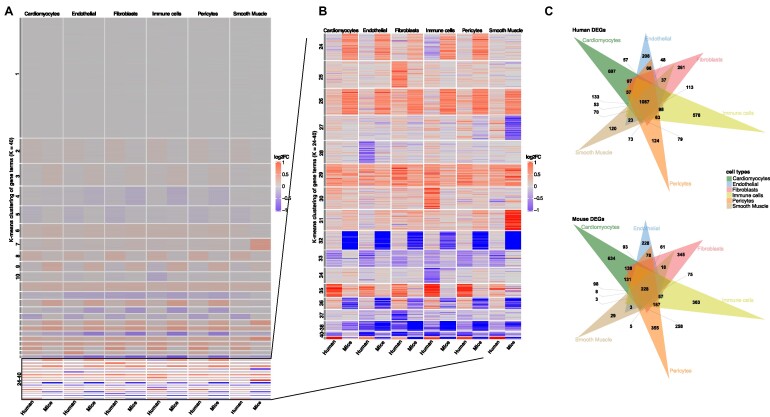
DEG analysis shows similar and different populations of regulation in gene expression patterns upon heart failure in humans and mice. (A) Heatmap of log2FC values (control vs HFrEF) for all genes and all cell types. The y-axis describes all genes (16,545) clustered by a *k*-means algorithm (*k* = 40). The x-axis shows the species and the additional clustering into the different cell types. Positive log2FCs are represented by red, while negative scores are given in blue. (B) Closeup of the 24–40 *k*-means clusters of log2FCs of genes in which most cell type–specific differences are observed. (C) Venn diagrams of all identified DEGs in human (top) and mouse (bottom) (log2FC > 0.1 and *P*-adjusted < 0.05).

We visualized all expression changes in 1 heatmap to further validate individual gene changes upon HFrEF (Fig. 4A, B). Thereby, we found that around 30% of the genes show no changes in their expression upon heart failure (Fig. 4A, cluster 1). Most expression changes were consistently observable in all cell types (clusters 2–23) and therefore appeared as general responses to injury that could not be attributed to individual cell types. However, the remaining 16 clusters showed cell type–specific expression patterns (Fig. 4B). For example, cluster 25 held a set of genes that showed increased expression of genes in human FBs, whereas cluster 28 in human ECs contained many genes that were downregulated. These changes were not detectable in other cell types for these genes and therefore of utmost interest to follow up on specific gene expression changes in species-specific cells. Similar patterns were found by observing commonly regulated genes (Fig. 4C). For humans, the largest number of DEGs was found in all cell types (1,087 DEGs). The second largest groups contained DEGs that were found only in the individual cell types (Fig. 4C; Supplementary Fig. S4A). Thus, we identified 687 DEGs specific to human CMs and 208 DEGs that could only be found in ECs. Determining the distribution of DEGs in mice revealed larger populations of cell type–specific genes and fewer DEGs, which were found in all populations (*n* = 228). Notably, we found far fewer DEGs in the mouse SMCs than in the human samples. However, this could be related to the total number of SMCs in mice, which was far less in mice than in human samples (Figs. 1C and 3A). This could explain the lower number of DEGs found in all cell types. When we excluded SMCs from the common DEG population, we observed a similar number of DEGs in all cell types as in humans previously (Supplementary Fig. S4B).

Further, we analyzed the highest upregulated genes per cell type in humans and mice along with the regulation of that gene in the other species. Hereby, we observed how the genes with the largest changes in human heart failure patients behave in the respective mouse model (Supplementary Fig. S3).

We observed that the expression of the most regulated genes in human cell types showed comparably less regulation in the mouse models. For example, we found *LDB2*, a gene of the LIM-domain family, in human CMs as highly upregulated (log2FC = 2.15) (Supplementary Fig. S3A). The LIM-domain family genes are well known as adapter molecules that allow the assembly of transcriptional regulatory complexes in CMs. However, in mice, *LDB2* was only mildly regulated upon HFrEF (log2FC = 0.38). Other genes such as the VEGF receptor *FLT1*, which is upregulated in human cardiomyocytes, showed a downregulation in mice CMs. This demonstrates that some genes have completely different expression patterns in humans and mice. However, some genes share similar regulation in their respective cell types. Thus, we observed that phosphodiesterase 4D (*PDE4D*) and ADP ribosylation factor like GTPase 15 (*ARL15*) showed similar changes in ECs. Among the 10 most upregulated genes in the mouse model data, we found 3 genes that also showed a significant increase in their expression in humans (*RBPJ, SLC9A9, RUNX1*) (Supplementary Fig. S3B). The other genes, however, showed little to no change. In contrast, if we investigate the expression changes in ECs, DEGs showed an opposite direction in their expression change (*RBPJ, PID1, SLC9A9*). These differential gene expressions in the cell types suggest that some cell type–specific responses may be different between human patients and mouse models.

### Pathway enrichment results in cardiomyocytes

**Figure 5:**
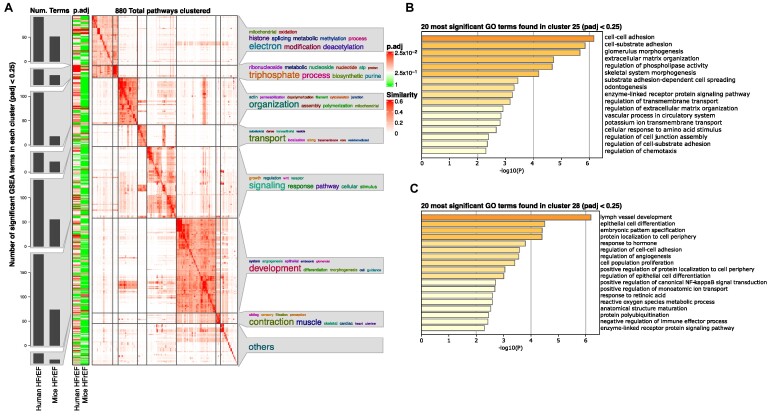
GSEA analysis reveals more regulated pathways in heart failure in human cardiomyocytes than in mice, with the terms found sharing many keywords. (A) Heatmap clustering of significant GSEA results (*P*-adjusted < 0.25) of DEGs found in human and mouse cardiomyocytes by similar GeneIDs in the pathways. Bar graphs are shown on the left y-axis representing the number of pathways found in the respective cluster for the given species and condition. In addition, the adjusted *P* value is color-coded from 1 (green) to the smallest *P* value found, ∼0.025 (red). On the right side of the y-axis, keywords describing the found pathways in that cluster are shown, where the size of the word represents its frequency in the terms (larger = most, smaller = less). (B) Bar graph showing the first 20 GO terms found by analyzing genes in cluster 25. Terms were sorted by their logarithmized and Bonferroni-adjusted *P* values, resulting in high significant pathways depicted first (*P*-adjusted < 0.05). (C) Bar graph similar to (B) terms found in a subsection of genes in cluster 28.

To address whether the relatively high number of significantly regulated genes indicates overall changes in pathways and pathological processes or whether the differences relate more to the alternative use of genes with similar functions in mice and humans, we further determined the implications for overall pathways in the individual cell types. Figure 4 shows a *simplifyEnrichment* heatmap cluster with word clouds of Gene Ontology (GO) terms regulated in human or mouse cardiomyocytes. We generally observed more significantly enriched gene set enrichment analysis (GSEA) terms in humans than in mice (Fig. 5A). Important pathway terms regarding mitochondrial energy production and the electron chain were enriched in both species. Other terms involving developmental processes were enriched in humans compared to mice. Additionally, we investigated the set of genes found in cluster 25 and cluster 28 in more detail (Fig. 4B and Fig. 5B, C). GO analysis on subsections of genes found in these clusters revealed a change in pathways associated with cell adhesion and extracellular processes (Fig. 5B). The second subsection of cluster 28 was associated with terms regarding cell differentiation processes, like “epithelial cell differentiation” or “angiogenesis” (Fig. 5C).

**Figure 6:**
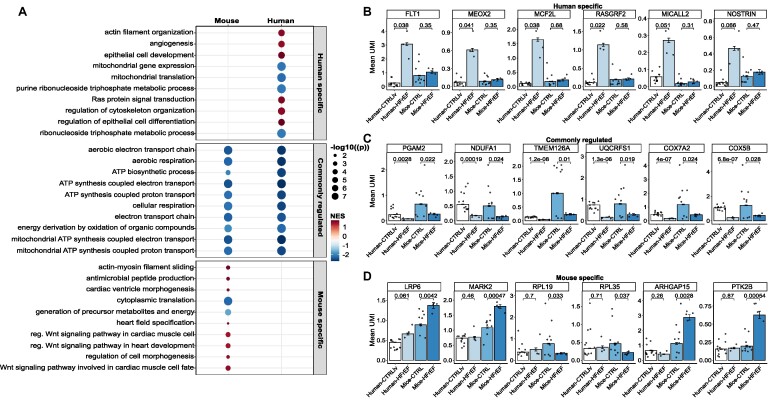
Common and distinct regulated pathways found in human and mouse cardiomyocytes. (A) Dot plot visualizing the 10 most significant pathways for terms only to be found regulated in humans, commonly regulated, and specific in mice. The size of the dots corresponds to the negative log10 of the Benjamini–Hochberg adjusted *P* value, and the color code represents the normalized enrichment score (NES), with upregulated pathways shown in red and downregulated pathways in blue. The y-axis depicts the description of the identified term. (B) Bar plot with mean values for the amount of unique molecular identifiers (UMIs) in the cells for the shown genes. The genes are identified to be dissimilarly regulated between humans and mice for pathways specifically found in humans. (C) Bar graph similar to (B) with mean values for UMIs in cells for genes downregulated in both species for commonly found terms. (D) Bar graph similar to (B) and (C) with mean values for UMIs in cells for genes that are uniquely found to be regulated in terms specifically identified in mice. *P* values above the certain groups were calculated by 2-sided Student’s *t*-test.

We identified cell-type specifically regulated pathways upon HFrEF. Therefore, we investigated how the enriched signaling pathways differ between humans and mice in cardiomyocytes. We observed larger differences for pathways that were specifically regulated in humans. Among the most regulated pathways, specifically detected in humans, we found the terms “actin filament organization” and “angiogenesis” (Fig. 6A). Genes associated with these pathways were explicitly upregulated in patients (Fig. 6B). These gene sets were not found among the regulated pathways in mice (Supplementary Table S4). Examples of angiogenesis-related genes that are specifically induced in human heart failure but not in mouse models include receptors such as the *VEGF*-receptor FLT1 or transcription factors like the mesenchyme homeobox protein 2 (MEOX2) (Fig. 6B). In addition, many GTPase regulatory genes were found specifically increased in humans, including *MCF2L* and *RASGRF2*, which are known to regulate *RAC1*, and *SPATA13*, which enables guanyl-nucleotide exchange factor activity [34, 35]. In contrast, we observed that signaling pathways mainly dealing with energy metabolism were commonly regulated in patients with heart disease as well as in mouse models. The genes included in pathways such as “ATP biosynthetic process,” “mitochondrial ATP synthesis,” “aerobic electron transport chain,” and “cellular respiration” showed significant downregulation compared to their corresponding control (Fig. 6C). These data suggest conservation of disturbed mitochondrial metabolism in both mice and humans upon heart failure.

On the other hand, pathways such as “Wnt signaling pathway,” “actin–myosin filament sliding,” and “regulation of cell morphogenesis” were upregulated specifically in the mouse HFrEF model (Fig. 6A). Genes associated with Wnt signaling include *LRP6*, a known inhibitor of cardiomyocyte proliferation [36], and the serine/threonine–protein kinase *MARK2*, which regulates the stability of microtubules through phosphorylation and inactivation of several microtubule-associated proteins [36].

Furthermore, we repeated the GSEA analysis with the identified ECs in the human and mouse model data to gain further insight into the different cell types (Supplementary Fig. S5). Here, we found human-specific regulated terms such as “cardiac contraction” and “regulation of axonogenesis” (Supplementary Fig. S5A) only in ECs but not in the previously analyzed CMs. The genes in these sets showed a distinct regulation only observed in human data (Supplementary Fig. S5B). When we examined the commonly regulated metabolic pathways, we found similar terms and changes in gene expression related to impaired mitochondrial metabolism in ECs as we had previously observed in CMs (Supplementary Fig. S5C). In ECs, we also found similar mouse-specific terms such as “cell morphogenesis” and the “Wnt signaling pathway” but also newly discovered pathways such as “positive regulation of steroid hormone secretion.” Steroid hormones have been shown to coordinate microvascular function in obese mice endothelium [37]. Based on these results, one might speculate that this regulatory function is mouse specific. GSEA analysis for all other cell types can be found in [38]. All source code for this study can be found in the article-specific GitHub repository [39].

## Discussion

The ever-growing number of published single-cell experiments enables scientists to deepen the knowledge about transcriptional changes of individual cell types and species-specific regulatory changes upon disease conditions. A particular combination of single-cell datasets from different species in the same UMAP projection allows the detection of well-conserved or species-specific regulatory networks [40–42].

Therefore, integrating datasets from different species with a well-curated list of orthologs has significant advantages and simplifies comparisons among species.

Here we propose *OrthoIntegrate*, an R-package that enables scientists to integrate single-cell datasets from different species into a shared dimensional space. To generate high-quality and uniquely mapped orthologous lists between different species, we implemented a new pipeline that increases the 1-to-1 assignment of ontologies to improve single-cell integration. Compared to the Ensembl orthologous list (Biomart), our pipeline results in up to 10% more uniquely assigned orthologs between human and mouse. Compared to the other databases OMA and InParanoid, *OrthoIntegrate* contains 8.6% and 9.3% more 1-to-1 orthologs (Fig. 3F).

*OrthoIntegrate* additionally contains functions that use the extended orthologous assignments to streamline the integration of single-cell datasets from humans and mice. Moreover, it is highly adaptable and can be easily customized to support other species.

We demonstrated the usability of combining cross-species single-cell data by using datasets of human and mouse heart failure with reduced ejection fraction.

In order to evaluate the species mixing and the biological conservation of different integration methods, we applied certain metrics from the scib package [32, 43], which were also suggested by Song et al. [32, 33, 43]. The results are summarized in Fig. 2D. We found that most batch correction scores improved by using *OrthoIntegrate*.

For biological conservation scores, we demonstrate that some metrics, like the “cell cycle conservation,” were improved by using *OrthoIntegrate*, which means that the variance caused by different cell cycle states of the cells was conserved via *OrthoIntegrate*. Other parameters, like the normalized mutual information (NMI) score, were reduced. But this score, for example, was strongly influenced by the cell-type labeling [33], which focused only on main cell-type groups in these datasets, regardless of the existence of subpopulation or mixed cell-type population clusters. In other words, subclusters of different cell types were not annotated in detail. Due to the increased numbers of features that are included in *OrthoIntegrate*, the clustering might be more diverged, likely by species-specific noncoding RNAs or other features, which are not included in the other databases. Therefore, the more divergent clustering, due to the increased number of features in *OrthoIntegrate* combined with the broad cell-type labeling, might explain the slightly reduced NMI scores. However, since various publications have shown that long noncoding RNAs have important regulatory roles in the heart [44–46], we think that these additional noncoding RNAs are an important resource to study species-specific responses to different disease conditions, especially in the field of heart failure.

Commonly regulated pathways upon heart failure reflect an evolutionary conserved transcriptomic answer to severe damage in heart cells. One example is the conserved downregulation of critical mitochondrial metabolic pathways, which provide ATP for the heart (Fig. 5 and Fig. 6A, C). As the heart is the most energy-consuming organ, maintaining mitochondrial function plays a critical role, and the decline in energy production limits heart function [47]. We could show that genes important for ATP biosynthesis and electron transport (e.g., *PGAM2, NDUFA1*, and *TMEM126A*) are consistently downregulated in heart failure. *PGAM2* and *NDUFA1* have been described in the context of heart disease in mice [48] and rats [49], respectively, but their role in humans is unknown.

Besides commonly regulated pathways, we found differences between humans and mice upon heart failure. In cardiomyocytes, genes associated with “angiogenesis” were specifically enriched in humans. For example, the *VEGF* receptor *FLT1* was specifically increased in the human samples. *FLT1* primarily mediates *VEGF* signaling in endothelial cells, but its role in cardiomyocytes, besides high expression [50], is less clear [51]. Functionally, *FLT1* was shown to partially mediate *VEGF*-induced cardiomyocyte differentiation [52] and regulate cardiomyocyte contractility in the embryonic zebrafish heart [53]. Cardiomyocyte-specific deletion of *FLT1* was shown to worsen cardiac remodeling and hypertrophy induced by pressure overload [54], suggesting that the specific upregulation in humans may represent a compensatory cardioprotective mechanism that might not be conserved in mice.

A second human CM-specific gene is *MEOX2*, which was assigned to “angiogenesis” because of its role in endothelial fatty acid transport [55]. *MEOX2* plays a critical role in the development of all muscle lineages [56]. In cardiomyocytes, *MEOX2* overexpression blocks proliferation during heart morphogenesis [57]. All of these human CM-specific genes have not been studied in mouse cardiomyocytes, and their human-specific regulation upon heart failure might be of utmost interest for future studies.

Among the pathways specifically enriched in mice, we found predominant expression of genes associated with Wnt signaling. Although most identified genes have not been directly linked to cardiomyocyte-specific functions, Wnt signaling critically regulates cardiac hypertrophy, remodeling, and regeneration [36, 58]. Therefore, these findings and the other identified species-specific pathways deserve more in-depth validation and investigation.

To further demonstrate the functionality of *OrthoIntegrate*, we integrated scRNA-seq data from human [43], mouse [59] and zebrafish [59, 60] brain tissue under an Alzheimer disease condition. Besides the evolutionary distance between these species, we could jointly cluster different cell types via *OrthoIntegrate* (Supplementary Fig. S6A–C) and detect commonly expressed marker genes within these cell clusters (Supplementary Fig. S6D–F).

In summary, our publicly available bioinformatic tool *OrthoIntegrate* simplifies the comparison of scRNA-seq datasets from humans and mice, and thereby we could identify conserved regulatory pathways upon heart failure. Furthermore, we identified cell type–specific differences in both species. Also, we showed pathways such as angiogenesis regulated explicitly in humans, and Wnt signaling pathways specifically regulated in mice.

We anticipate that this study shows the benefits of the joint analysis of scRNA-seq data through *OrthoIntegrate*. Due to the growing number of scRNA-seq datasets, we hope that *OrthoIntegrate* encourages other scientists to perform comparative analysis between different species, thereby increasing knowledge about conserved or species-specific pathway responses in various diseases. This could improve the effective development of novel treatment strategies for heart failure or other diseases.

### Limitations

The main limitation of our ortholog assignment and sample integration pipeline is the dependence on reliable databases for orthologous lists. Another problem with this approach is that it fails to consider the biological functions of the possible orthologs but selects the ortholog with the highest sequence similarity. Second, our biological example has some limitations. While a decent number of healthy controls are available, the number of patients with HFrEF is limited. Knowing the biological heterogeneity of heart failure and comorbidities, variations are expected and the samples may not represent the representative and most common spectrum of heart failure. Finally, although the mouse model used is commonly applied in cardiovascular research, there are significant limitations due to the lack of underlying coronary artery disease and therapeutic pharmacological and interventions as done in humans. The integration of increasingly available published data both from alternative mice models and data derived from human samples will allow a refined comparative analysis in the future.

## Methods

### Study samples

The human heart samples used as controls were provided from the PRJEB39602 (Human Cell Atlas) project published in 2020. The heart tissue was obtained from deceased transplant organ donors who were between 45 and 70 years old and showed unremarkable cardiovascular history. The healthy mice samples (CTRL: n4–n9) were gathered by Vidal et al. (2019) [61] and can be found using the Array Express Data Portal under E-MTAB-7869 (Supplementary Table S1).

Heart samples from patients with HFrEF were gathered for this study from the Frankfurt University Hospital and subsequently processed at the Institute of Cardiovascular Regeneration (Frankfurt am Main, Germany), where the processed mice samples (CTRL: n1–n3, HFrEF: n1–n4) were also gathered and sourced.

Nuclear isolation steps and single-nucleus RNA sequencing library preparation were conducted as described in Nicin et al. [62].

The human heart failure samples as well as the mice control and heart failure samples are published in Array Express with the accession E-MTAB-13264 (Supplementary Table S1).

In order to provide another species and disease condition, we applied *OrthoIntegrat*e on humans, mice, and zebrafish in the Alzheimer disease (AD) condition. Therefore, we gathered scRNA-seq data from the prefrontal cortex (location matched) of human, mouse, and zebrafish via scRead (human and mouse data; disease, *n* = 2; healthy, *n* = 2) [63] and GEO (GSE118577; *n* = 3). The human and mouse samples originate from GSE129308 [43] and GSE143758 (AD) & GSE143758 (Healthy), respectively.

### Single-cell preprocessing

Single-cell RNA-seq results were processed by CellRanger (10x Genomics) version 6.1.1 software. The first step consisted of demultiplexing and processing raw base count files by the implemented *mkfastq* tool. The human raw reads were mapped to the reference genome hg38 (GRCh38-2020) using CellRanger count, whereas the mouse raw reads were mapped to the reference genome mm10 (GRCm38-2020). The secondary data analysis was conducted using the Seurat 4.1.0 package in R. The datasets were first combined into a Seurat object and then subjected to a filtering process. Barcodes with too low (<300) or too high number of genes (>6,000) were sorted out and not considered further in the data analysis. In addition, barcodes with too low (<500) and too high read counts (>15,000) were also sorted out. To further ensure no apoptotic cells or doublets were analyzed, we discarded barcodes with a high percentage of mitochondrial content (>5%). The filtered gene counts were then logarithmized and normalized according to the tutorial for data analysis with Seurat. Baseline characteristics for the samples can be found in Supplementary Table S1.

### Ortholog assignment and sample integration

In order to ensure the integration of single-cell datasets from different species, we coded a function to assign animal model orthologs to the human nomenclature (or vice versa) using gene transfer format (GTF) files provided by Ensembl (GRCh38, GRCm38). In order to detect only well-annotated genes between the species, predicted genes were removed. Afterward, orthologs to the human genes were determined using the R package biomaRt. This assigned the majority of genes in our human GTF file to at least 1 ortholog. If there were several entries of possible orthologs in the Ensembl database, a protein sequence comparison was initiated. Therefore, protein sequences were retrieved from the Uniprot database for the human gene and all possible orthologs in the second species. These sequences were then aligned using the R package Biostrings 2.60.2. The alignment score was calculated based on the Needleman–Wunsch global alignment algorithm [28] with substitution matrices. For nucleotide sequences, the *nucleotideSubstitutionMatrix* function was used to produce a substitution matrix for all IUPAC nucleic acid codes based upon match and mismatch parameters. BLOSUM50 matrix was retrieved from the NCBI Matrix Compendium for the protein sequence. The gene IDs with the highest amino acid sequence similarity between their canonical sequences were assigned. If there were no entries for canonical sequences in Uniprot, the nucleotide sequence similarity comparison was initiated. For this step, the unpredicted mRNA sequences for the gene in the first species and for the possible orthologs in the second species were obtained from the NCBI database and aligned analogously to the previous step. If no unpredicted mRNA was available for an entry, the function retrieved the unpredicted noncoding RNA of the gene. This ensured that noncoding genes without mRNAs could still be assigned correctly. In case both RNA sequences were not retrievable, predicted versions of mRNA and noncoding RNA were retrieved. If all these assignment steps were not successful, the Levenshtein distance was used to compare the ID symbols for possible orthologs, and the ortholog with the lowest Levenshtein distance was selected.

Many long noncoding RNAs are not listed in ortholog databases; therefore, a final lowercase matching step was performed to assign genes like *Malat1* to the human *MALAT1*. With this globally applicable list of orthologs between species, the datasets were now filtered by these and then merged into 1 object using Seurat’s canonical correlation analysis (CCA) integration.

### Clustering, metrics calculation, and annotation

To classify cells into clusters based on their expressed genes, we used the *FindNeighbors* and *FindClusters* (resolution parameter = 0.3) function implemented in Seurat. These clusters are determined by applying the shared nearest neighbors (SNN) clustering algorithm and the UMAP dimension reduction.

Calculations of the silhouette coefficient are based on computing a distance matrix based on the cell embedding matrix for principal component analysis (PCA) performed by Seurat. This distance matrix includes the information of cell–cell distance, which is necessary for calculating the silhouette coefficient with our calculated clusters in the function *silhouette* of the cluster package (version 2.1.4). Additionally, the coefficients of the samples were averaged for each object. For applying the Python scib package, we converted our Seurat objects into Anndata objects using the zellkonverter package (version 1.10.1). We computed graph connectivity, principal component regression comparison, silhouette batch, kBET, LISI, and cell cycle conservation scores for defining the species mixing score. Furthermore, the bioconservation score was calculated by computing the species type LISI, isolated labels F1 score, and the previously mentioned silhouette coefficient. The total score was then calculated by a weighted addition of species mixing score and bioconservation score (0.5 * species mixing score + 0.5 * bioconservation score). We provided the UniprotIDs of the orthologous lists obtained with the tools to be compared to the Orthology Benchmark web service to calculate the Schlicker similarity scores for enzyme classification conservation and GO conservation.

The orthologous lists for OMA, Biomart, and InParanoid were created by following their introductions on their tool descriptions and by using the same GTF files as before (GRCh38, GRCm38).

For the assignment of cell clusters to cell types, we used a reference object that we had previously manually annotated with marker genes from Tombor et al. [64]. Here, the R package SingleR can be used to adopt marker genes that were used for the previous annotation of clusters of the reference object. These were then transferred and compared to marker genes of the cell clusters of our object to be annotated. Thus, a reproducible annotation can be guaranteed with the help of an exactly annotated dataset.

### Differential gene expression analysis and GO analysis

Detection of DEGs for the cell type–specific clusters was performed by the hurdle model of the MAST package (version 1.20.0). Results were filtered by their Bonferroni-adjusted *P* value (*P*-adjusted < 0.05). The totality of DEGs was represented by Sankey plots created with the R package networkD3 (version 0.4). Additionally, bar plots were created using R package ggplot2, representing human DEGs and their regulation in mice. DEGs were also divided according to their species and cell-type assignment and then visualized for DEGs with a positive log2FC and separately in another plot, for DEGs with a negative log2FC. Here, DEGs occurring in both human and mouse for the respective cell type were pooled. Visualization was done in the form of a Circos plot (R package circlize 0.4.14). The gene regulation heatmap was created using the log2FC of all identified genes and a *k*-means clustering (*k* = 40) (R package ComplexHeatmap 2.16.0). Visualization of distinct and similar populations of genes in the analyzed cell types per species was achieved by creating Venn diagrams with the Jvenn webtool.

GSEA was performed using the R package clusterProfiler (version 4.2.2) and the GO Database. GSEA terms were calculated separately for each cell type. The terms were sorted according to the Benjamini–Hochberg adjusted *P* value and evaluated according to their “normalized enrichment distribution,” which gives information about the regulation of the genes in the described pathway. A heatmap was created by clustering the GSEA terms by their similar geneIDs (R package simplifyEnrichment 1.10.0). Additionally, the GSEA results were plotted in dot plots. Specifically, for genes described in the pathway, the standard error of the mean bar plot was created (for their averaged unique molecular identifiers) by using the R package ggplot2. GO analyses were performed using the subsection of genes found in cluster 25 and cluster 28 as input for the webtool Metascape.

## Availability of Source Code and Requirements

Project name: *OrthoIntegrate*

Project homepage: https://github.com/MarianoRuzJurado/OrthoIntegrate [38]

Operating system(s): Platform independent

Programming language: R

Other requirements: certain R-packages (Seurat (>= 4.2.0), ggplot2 (>= 3.3.6), ggpubr (>= 0.4.0), biomaRt (>= 2.52.0), rtracklayer (>= 1.56.1), mygene (>= 1.32.0), UniprotR (>= 2.2.2), RecordLinkage (>= 0.4–12.3), Biostrings (>= 2.64.1), rentrez (>= 1.2.3), stringr (>= 1.4.1), svglite (>= 2.1.0), dplyr (>= 1.1.2), tidyr (>= 1.3.0)

License: GNU GPL

RRID:  **SCR_025029, OrthoIntegrate**

## Supplementary Material

giae011_GIGA-D-23-00222_Original_Submission

giae011_GIGA-D-23-00222_Revision_1

giae011_GIGA-D-23-00222_Revision_2

giae011_Response_to_Reviewer_Comments_Original_Submission

giae011_Response_to_Reviewer_Comments_Revision_1

giae011_Reviewer_1_Report_Original_Submission Ruoyan Li -- 9/3/2023 Reviewed

giae011_Reviewer_2_Report_Original_Submission Yinqi Bai -- 10/16/2023 Reviewed

giae011_Reviewer_2_Report_Revision_1 Yinqi Ba -- 1/26/2024 Reviewed

giae011_Supplemental_Files

## Data Availability

The single nuclei data for humans have been deposited in the Human Cell Atlas database and can be accessed through the HCA Data Portal [65]. The mice sequencing data are available through ArrayExpress under the accession number E-MTAB-7869. All supporting data and materials are available in the *GigaScience* database, GigaDB [66].

## Additional Files

**Supplementary Fig. S1**. Integration process of human/mouse snRNA-seq data. (A) UML-Activity-Flowchart showing ortholog assignment pipeline for human to mouse gene symbols. First, the GTF for humans (GRCh38) is used to get all annotated gene nomenclatures. Then all genes are filtered out, which are only predicted and not clearly detected. This list is now searched for orthologs using the Ensembl database; all 1:1 assignments can be included in our orthologous list. In the case of multiple assignments, all possible replacements are examined according to their protein sequence and an alignment score is calculated according to the global sequence alignment. If there is no protein sequence in the Uniprot database, the alignment score is calculated based on the nucleotide sequence using the NCBI database. Now the gene with the best result is set as an ortholog. All unassigned genes are additionally compared with the GTF file of GRCm38 using a lowercase matching, and if there is a match, they will be added to the ortholog list. If all these approaches for a gene do not result in an ortholog, a Levenshtein distance score is calculated based on their gene names. (B) Single-cell integration pipeline showing steps performed to integrate human and mouse scRNA-seq data in a joined UMAP projection. The scRNA-seq data from our human and mouse samples are first converted into Seurat objects and normalized. After that, clustering takes place and cell types can be determined. Using the orthologous list from our ortholog assignment algorithm, the objects can be subsetted according to the genes found and their nomenclature unified. This is followed by an integration into a single object and a clustering step.

**Supplementary Fig. S2**. Overlapping of human and mouse cells after Seurat integration with a tool-specific orthologous list. (A) UMAPs showing human cells (red) and mice cells (blue) in a common UMAP projection for each tool used for integrating the data. First, UMAP was performed on an object made with an orthologous list of OMA, followed by Biomart and InParanoid. The last UMAP shows the projection for the *OrthoIntegrate* pipeline. (B) Similar to (A), UMAPs are shown to visualize the clustering created with the R package Seurat using the Louvain algorithm. The objects are found in the same order as previously described. (C) Similar to (A) and (B), UMAPs are shown with the cell-type annotation. The order is maintained as in (A) and (B) (same parameters are used for each UMAP; resolution = 0.3).

**Supplementary Fig. S3**. Circos plots of DEGs show specific and similar expressed DEGs. (A) Circos plot showing the 10 most upregulated genes in human HFrEF (log2FC), separated for all cell types. Red line indicates the log2FC for human DEGs, while the blue line indicates the log2FC of the corresponding mouse gene. (B) Circos plot similar to (A) illustrates the 10 most upregulated genes in mice HFrEF samples in comparison to the regulation of these genes in humans.

**Supplementary Fig. S4**. DEG analysis shows population of shared and cell type–specific genes across cell types in humans and mice. (A) Upset plot of human DEGs found in the analyzed cell types. The groups are sorted by their intersection size and plotted on the x-axis of the plot. Additionally, the overlapping groups are visualized by a connected dot plot. The size of the DEGs per cell type is shown on the y-axis. (B) Upset plot of mouse DEG populations similar to previous plot in (A).

**Supplementary Fig. S5**. GSEA analysis shows regulated pathways upon heart failure in human and mouse endothelial cells. (A) Dot plot visualizing the 10 most significant pathways for terms only to be found regulated in humans, commonly regulated, and specific in mice endothelial cells. The size of the dots corresponds to the negative log10 of the Benjamini– Hochberg adjusted *P* value, and the color code represents the normalized enrichment score (NES), with upregulated pathways shown in red and downregulated pathways in blue. The y-axis depicts the description of the identified term. (B) Bar plot with mean values for the amount of unique molecular identifiers (UMIs) in the cells for the shown genes. The genes are identified to be dissimilarly regulated between humans and mice for pathways specifically found in humans. (C) Bar graph similar to (B) with mean values for UMIs in cells for genes downregulated in both species for commonly found terms. (D) Bar graph similar to (B) and (C) with mean values for UMIs in cells for genes that are uniquely found to be regulated in terms specifically identified in mice. *P* values above the certain groups were calculated by the 2-sided Student’s *t*-test.

**Supplementary Fig. S6**. Integration of human, mouse, and zebrafish scRNA-seq prefrontal cortex data of healthy and Alzheimer disease patients. (A) UMAP showing human cells (red), mice cells (blue), and zebrafish cells (green) in a common UMAP projection. (B) UMAP with defined clusters according to Seurat’s clustering. Cells of mouse, human, and zebrafish origin commingled in the astrocyte cluster (green). Excitatory (red) and inhibitory (green) neurons are mostly present in human data. (C) Bar plot showing cell composition of cell types in human (red), mice (blue), and zebrafish (green) samples. Samples were grouped based on their origin into human, mouse, and zebrafish controls from the prefrontal cortex (Healthy) and Alzheimer disease human, mouse, and zebrafish (Alzheimer). Cell types were then analyzed for their composition from the previously mentioned groups and plotted. (D) Dot plot depicting the average expression levels and expression proportions in human samples of the top 15 feature genes for the found cell types. The size of the dot represents the proportion of cells expressing the indicated gene within a cell type, and the color indicates the average expression level of cells. (E) Dot plot depicting the average expression levels and expression proportions in mice samples of the top 15 feature genes for found types. Similar to (D), the size of the dot represents the proportion of cells expressing the indicated gene within a cell type, and the color indicates the average expression level of cells. (F) Dot plot depicting the average expression levels and expression proportions in zebrafish samples of the top 15 feature genes for the found cell types. Similar to (D and E), the size of the dot represents the proportion of cells expressing the indicated gene within a cell type, and the color indicates the average expression level of cells.

**Supplementary Table 1:** Baseline charachteristics of human and mouse samples.

**Supplementary Table 2:** Number of mouse genes before and after ortholog assignement

**Supplementary Table 3:** Human and mouse DEGs upon HFrEF

**Supplementary Table 4:** Human and gene-set-enrichment analysis (GSEA) upon HFrEF

**Supplementary Table 5:** Human and gene-set-enrichment analysis (GSEA) upon HFrEF in endothelial cells

**Supplementary Table 6:** Number of cells per sample and celltype

## Abbreviations

AD: Alzheimer disease; bASW: batch average silhouette width; BCS: bioconservation score; CCA: canonical correlation analysis; CCC: cell cycle conservation; CM: cardiomyocyte; DEG: differentially expressed gene; EC: endothelial cell; FB: fibroblast; GC: graph connectivity; GO: Gene Ontology; GSEA: gene set enrichment analysis; GTF: gene transfer format; HFrEF: heart failure patients with reduced ejection fraction; IC: immune cell; ILF1: isolated labels F1 score; LAD: left anterior descending coronary artery model; lncRNA: long-non coding RNA; mRNA: messenger RNA; NC: neuronal cell; NES: normalized enrichment score; NMI: normalized mutual information; PC: pericyte; PCA: principal component analysis; PCR: principal component regression comparison; SC: silhouette coefficient; scRNA-seq: single-cell RNA sequencing; SnRNA-seq: single-nucleitide RNA sequencing; MC: smooth muscle cell; SMS: species mixing score; SNN: shared nearest neighbor; UMAP: Uniform Manifold Approximation and Projection; UMI: unique molecular identifier.
