# Supplementary material for: Improved integration of single-cell transcriptome data demonstrates common and unique signatures of heart failure in mice and humans
Source: Gigascience. 2024 Apr 4;13:giae011. doi: 10.1093/gigascience/giae011 (PMC10993718; doi:10.1093/gigascience/giae011)
Supplement: giae011_GIGA-D-23-00222_Original_Submission [file giae011_giga-d-23-00222_original_submission.pdf]

GigaScience

Improved integration of single cell transcriptome data demonstrates common and unique signatures of heart failure in mice and humans

--Manuscript Draft--

**Manuscript Number:**	GIGA-D-23-00222
**Full Title:**	Improved integration of single cell transcriptome data demonstrates common and unique signatures of heart failure in mice and humans
**Article Type:**	Research
**Funding Information:**	Deutsche Forschungsgemeinschaft (DFG) (Exc2026/1) — Dr. David John<br>Dr. Rolf M. Schwiete Stiftung (Projekt 08/2018) — Prof. Dr. Stefanie Dimmeler
**Abstract:**	Background<br><br>Cardiovascular research heavily relies on mouse (mus musculus) models to study disease mechanisms and to test novel biomarkers and medications. Yet, applying these results to patients remains a major challenge and often results in non-effective drugs. Therefore, it is an open challenge of translational science to develop models with high similarities and predictive value. This requires a comparison of disease models in mice with diseased tissue derived from humans.<br><br>Results<br><br>To compare the transcriptional signatures at single cell resolution, we implemented an integration pipeline called OrthoIntegrate which uniquely assigns orthologs and therewith merges single cell data (scRNA-SEQ) of different species. The pipeline has been designed to be as easy to use and is fully integrable in the standard Seurat workflow.<br>We applied OrthoIntegrate on scRNA-SEQ from cardiac tissue of heart failure patients with reduced ejection fraction (HFrEF) and scRNA-SEQ from the mice after chronic infarction, which is a commonly used mouse model to mimic HFrEF. We discovered shared and distinct regulatory pathways between human HFrEF patients and the corresponding mouse model. Overall, 54% of genes were commonly regulated including major changes in cardiomyocyte energy metabolism. However, several regulatory pathways, e.g. angiogenesis, were specifically regulated in humans.<br><br>Conclusion<br><br>The demonstration of unique pathways occurring in humans indicate limitations on the comparability between mice models and human HFrEF and show that results from the mice model should be validated carefully. OrthoIntegrate is publicly accessible (https://github.com/MarianoRuzJurado/OrthoIntegrate) and can be used to integrate other large data sets to provide a general comparison of models with patients data.
**Corresponding Author:**	David John, Ph-D<br>Goethe-Universitat Frankfurt am Main<br>Frankfurt am Main, Hessen GERMANY
**Corresponding Author Secondary Information:**	
**Corresponding Author's Institution:**	Goethe-Universitat Frankfurt am Main
**Corresponding Author's Secondary Institution:**	
**First Author:**	Mariano Ruz Jurado
**First Author Secondary Information:**	
**Order of Authors:**	Mariano Ruz Jurado

	Lukas S. Tombor
	Mani Arsalan
	Tomas Holubec
	Fabian Emrich
	Thomas Walther
	Wesley Abplanalp
	Ariane Fischer
	Andreas M. Zeiher
	Marcel H. Schulz
	Stefanie Dimmeler
	David John, Ph-D
**Order of Authors Secondary Information:**	
**Additional Information:**	
**Question**	**Response**
Are you submitting this manuscript to a special series or article collection?	No
**Experimental design and statistics**<br><br>Full details of the experimental design and statistical methods used should be given in the Methods section, as detailed in our Minimum Standards Reporting Checklist. Information essential to interpreting the data presented should be made available in the figure legends.<br><br>Have you included all the information requested in your manuscript?	Yes
**Resources**<br><br>A description of all resources used, including antibodies, cell lines, animals and software tools, with enough information to allow them to be uniquely identified, should be included in the Methods section. Authors are strongly encouraged to cite Research Resource Identifiers (RRIDs) for antibodies, model organisms and tools, where possible.<br><br>Have you included the information requested as detailed in our Minimum	Yes

Powered by Editorial Manager® and ProduXion Manager® from Aries Systems Corporation

Standards Reporting Checklist?	
**Availability of data and materials**<br><br>All datasets and code on which the conclusions of the paper rely must be either included in your submission or deposited in publicly available repositories (where available and ethically appropriate), referencing such data using a unique identifier in the references and in the “Availability of Data and Materials” section of your manuscript.<br><br>Have you have met the above requirement as detailed in our Minimum Standards Reporting Checklist?	Yes

Powered by Editorial Manager® and ProduXion Manager® from Aries Systems Corporation

Main Manuscript
Click here to access/download;Manuscript;GigaScience Submission Final.docx

Improved integration of single cell transcriptome data demonstrates common and unique signatures of heart failure in mice and humans

Mariano Ruz Jurado[1,2,3], Lukas S. Tombor[1,2], Mani Arsalan[4], Tomas Holubec[4], Fabian Emrich[4], Thomas Walther[2,3,4], Wesley Abplanalp[1,2,3], Ariane Fischer[1], Andreas M. Zeiher [1,2,3], Marcel H. Schulz[1,2,3], Stefanie Dimmeler[1,2,3], David John[1,3]

1.) Institute of Cardiovascular Regeneration, Theodor-Stern-Kai 7, 60590 Frankfurt am Main

2.) German Centre for Cardiovascular Research (DZHK), Frankfurt am Main, Germany

3.) Cardio-Pulmonary Institute (CPI), funded by the German Research Foundation (DFG)

4.) Department of Cardiovascular Surgery, Goethe University Hospital, 60590 Frankfurt am Main

Abstract

Background

Cardiovascular research heavily relies on mouse (*mus musculus*) models to study disease mechanisms and to test novel biomarkers and medications. Yet, applying these results to patients remains a major challenge and often results in non-effective drugs. Therefore, it is an open challenge of translational science to develop models with high similarities and predictive value. This requires a comparison of disease models in mice with diseased tissue derived from humans.

Results

To compare the transcriptional signatures at single cell resolution, we implemented an integration pipeline called *OrthoIntegrate* which uniquely assigns orthologs and therewith

merges single cell data (scRNA-SEQ) of different species. The pipeline has been designed to be as easy to use and is fully integrable in the standard Seurat workflow.

We applied *OrthoIntegrate* on scRNA-SEQ from cardiac tissue of heart failure patients with reduced ejection fraction (HFrEF) and scRNA-SEQ from the mice after chronic infarction, which is a commonly used mouse model to mimic HFrEF. We discovered shared and distinct regulatory pathways between human HFrEF patients and the corresponding mouse model. Overall, 54% of genes were commonly regulated including major changes in cardiomyocyte energy metabolism. However, several regulatory pathways, e.g. angiogenesis, were specifically regulated in humans.

Conclusion

The demonstration of unique pathways occurring in humans indicate limitations on the comparability between mice models and human HFrEF and show that results from the mice model should be validated carefully. *OrthoIntegrate* is publicly accessible (https://github.com/MarianoRuzJurado/OrthoIntegrate) and can be used to integrate other large data sets to provide a general comparison of models with patients data.

Keywords

cross-species analysis, cardiovascular disease, heart failure with reduced ejection fraction, coronary artery ligation, single cell integration, cross species integration workflow

Introduction

Animal experiments are a powerful tool to improve our understanding of pathophysiological conditions and to predict responses to new therapeutic approaches (1). However, due to ethical considerations they are controversially discussed (2), and their predictive capacity for toxicity and drug responses is limited (3, 4). Especially mice are commonly used to model

human diseases as they are relatively inexpensive, have short generation times and have large numbers of offspring. Additionally mice have a relatively close physiological and phylogenetic relationship with humans (5), (6). Mice protein-coding genes are on average 85% identical to humans (4) and over 90% of both genomes have regional conserved synteny (7). Due to these advantageous breeding characteristics and their high sequencing conservation to humans, hundreds of different mouse models have been developed to study human diseases (8) like heart failure (9) or even diseases that do not occur naturally in mice like Alzheimer's or Parkinson's disease (10).

To study cardiovascular diseases, which remain the leading cause of morbidity and mortality in the aging society, the ligation of the left anterior descending coronary artery model (LAD) is often used to induce myocardial infarction, which results in ischemic heart failure with reduced ejection fraction (HFrEF) (11, 12). Thereby, the LAD is ligated to mimic the clotted artery as it occurs after infarction. While short term reperfusion then allows to mimic the reopening of the coronary artery by catheter based interventions, often chronic ligation is used to induce heart failure over the course of > 4 weeks. As this method describes a similar decline in heart function, scientists use LAD mouse models to simulate HFrEF and develop and test new therapeutic strategies (13–15). Patients who suffer from HFrEF, are unable to pump sufficient amounts of blood to meet the demands of body organs (16).

To address the comparability of HFrEF in human to mouse models, we used single nuclei RNA sequencing data, enabling us to assess transcriptional regulatory pathways in all cardiac cell populations with high resolution and accuracy (17, 18). In order to analyze scRNA-seq data from various samples, integration pipelines were developed to combine individual cells from different subjects into clusters with similar expression patterns (18, 19). Yet these bioinformatic tools can only integrate datasets from identical species. Several studies developed algorithms to compare mRNA expression patterns across species (20–22). However, a standardized and easy way to compare single cell/nuclei RNA sequencing data sets of human and mouse by directly integrating the data is still missing (18, 23, 24). To

overcome these limitations and the highly increasing demand for comparison of various organisms prompted us to develop a R package called *OrthoIntegrate*. It features a pipeline for integration of single cell datasets and orthologue assignment, allowing the simple integration of data from mouse models and human patients. For the orthologue assignment process, we implemented an algorithm in the workflow that adjusts the different nomenclature between species before the integration takes place, by using the databases of Ensembl, NCBI, and Uniprot. (25–27). Using our newly established pipeline, which is completely compatible with standard seurat workflows, we explored the gene expression patterns in mouse models of HFrEF compared to human samples. While 54% of genes were commonly regulated in both species, we also observed significant differences in differentially expressed genes and regulated pathways in patients with heart diseases than in the corresponding mouse model.

Results

One to one orthologue assignments

To integrate single cell data from different species, we established a table of gene names, which contains one human gene for each mouse gene, by which it will be replaced (one-to-one orthologs).

In order to generate these one-to-one orthologs, we utilized the Needleman-Wunsch algorithm (28) to perform a pairwise global alignment. This calculation determines alignment scores based on differences in the amino acid or nucleotide sequences. In case no orthologues were found, nor a protein- or nucleotide sequence is available for a particular gene, a lowercase matching of the human gene is searched for in the mouse gene database (Suppl. Fig. 1A).

The Ensembl database assigned a total of 21,428 mouse orthologues to our human gene ID symbols. However, only 77% (16,573) of these were uniquely assigned. Through our *OrthoIntegrate* pipeline, we increased the number of assignments to 82% (17,504). Hereby, 714 mouse genes were assigned by protein sequence alignment, 89 through nucleotide

sequence alignment, 42 by using the Levenshtein distance between gene names and 86 using our lowercase matching approach. Most of the latter ones were long non coding RNAs with identical gene names. We then proceeded by filtering the human and mice data by these orthologues in our pipeline and replaced the mice nomenclature by the human nomenclature for the corresponding samples (Suppl. Fig. 1B). In the end, we could assign ~82% of the mice genes to human orthologues (Suppl. Table 2). Replacing mouse gene names with the human orthologue allowed us to integrate the human patient data with the mouse model data into one single cell object (Fig. 1A).

Comparison to other integration methods

We carefully inspected our data to determine species specific distribution by creating UMAP plots of all cells in our integrated object. Figure 1B shows that cells of mouse and human origin commingled in all clusters, which shows a successful integration based on the cell types and not on the species. We additionally compared our *OrthoIntegrate* pipeline to other orthologue databases and tools to assess the advantages of our orthologue assignments. For this purpose, we created the same scRNA-SEQ datasets using the different orthologue lists OMA, Biomart and InParanoid (29–31). Visualization of the integration by UMAP plots shows an integration of human and mouse-derived cells in the individual cell clusters also with the alternative orthologous list (Suppl. Fig. 2). To assess the quality of the clustering, we calculated silhouette coefficients, which measures the quality of the clustering independent from the number of clusters. Integration by *OrthoIntegrate* resulted in the highest silhouette coefficients compared to the other orthologue databases, suggesting an improved clustering (Suppl. Fig. 3A, Suppl. Table 3). Additionally, it is noteworthy to mention that our pipeline achieved by far the most 1:1 protein coding and lncRNA coding orthologous pairs in comparison to the other described methods (Suppl. Fig 3B-C).

Cell type composition in human and mouse upon HFrEF

The absence of species specific clusters in the combined UMAP plot confirms that human and mouse hearts comprise similar cell types and gene expression patterns (Fig. 1B). This is additionally verified by similar cell type specific marker genes in both species in the different cell clusters (Fig. 1D-E). The specific marker genes allowed the annotation of the clusters into cardiomyocytes (CMs), pericytes (PCs), smooth muscle cells (SMCs), fibroblasts (FBs), endothelial cells (ECs), immune cells (ICs), as well as neuronal cells (NCs) (Fig. 1C). In addition, we analyzed how the distribution of cell types was affected by heart failure phenotype. Thereby a 20% decrease in human CM was observed when comparing the control samples with the HFrEF samples (45% -> 25%) (Fig. 1D). However, in mice, there is no difference in the numbers of CMs between the infarcted and control mice (both comprise about ~25% CM) (Fig. 1D). Furthermore, we found differences in the distribution of ECs in the human versus mouse samples. Specifically, we observed a significant increase in the EC population in samples from HFrEF patients (~30 %) compared to healthy hearts (~8%). In contrast, we noticed a decreased EC numbers in mice upon infarction (from 25% in controls to 18% after chronic infarction). Minor changes are also observed in the contributions of other cell types (Fig. 1D).

Differential gene expression between mice and humans

The differentially expressed gene (DEG) analysis, showed strong similarities in the regulated genes upon HFrEF. However, some genes showed differences in their expression patterns. Mainly when the cell types were analyzed individually. Overall, we found a comparable number of DEGs in both species (4141 in humans, 4654 in mice).

The average of commonly regulated genes per cell type (Fig 2A; left side) showed that around 54% of DEGs found in humans were also regulated in mice, with minor differences between cell types. Up-regulated genes showed a generally higher comparability compared to down-regulated genes (Fig. 2B). Only in smooth muscle cells many more human specific DEGs were regulated in opposite directions (Fig. 2B, right upper panel). Averaging the mouse regulated

DEGs (Fig 2A; right side) showed that only about 34% of the cell type specific DEGs in mice were regulated in humans, indicating a more substantial transcriptional effect of the LAD model compared to the human disease.

Figure 2B separately shows the up (top panel) and down (lower panel) regulated genes in humans and their regulation in mice. For the upregulated genes in humans, around 50-70% of the corresponding mouse genes were also upregulated, around 25% were not regulated and only about 5-20% percent were regulated in the opposite direction. Suggesting that overall activation occurs mainly in similar expression pathways across all cell types. In the downregulated genes in humans we observed a strikingly low number of commonly regulated genes in cardiomyocytes. There, only 23.3% of the downregulated genes were also decreased in mice. Most of them were either not regulated (48.2%) or even upregulated in mice (28.5%). The other cell types show a higher percentage of commonly downregulated genes.

We visualized all expression changes in one heatmap to further validate individual gene changes upon HFrEF (Fig. 3 A/B). Thereby, we found that around 30% of the genes show no changes in their expression upon heart failure (Fig. 3A, cluster 1). Most expression changes are consistently observable in all cell types (k = 2-23) and therefore appear as general responses to injury which cannot be attributed to individual cell types. However, the remaining 16 clusters show cell type specific expression patterns (Fig. 3B). For example, in cluster 25 we see a set of genes that show increased expression of genes in human FBs. In addition, we have a set of genes that are negatively regulated in cluster 28 in human ECs. These changes are not detectable in other cell types for these genes and are therefore of utmost interest to follow up on specific gene expression changes in species specific cells. Similar patterns were found by observing commonly regulated genes (Fig. 3C). For humans, the largest number of DEGs were found in all cell types (1087 DEGs). The second largest groups contain DEGs that are found only in the individual cell types (Fig. 3C; Suppl. Fig. 5A). Thus, we identified 687 DEGs specific to human CM and 208 DEGs that can only be found in ECs. If we now determine the distribution of DEGs in mice, one finds larger populations of cell type

specific genes and fewer, which are found in all populations (n = 228). Notably we found far fewer DEGs in the mouse SMCs than in the human samples. However, this could be related to the total number of SMCs in mice, which is far less in mice than in human samples (Fig. 1C & 2A). This could explain the lower number of DEGs found in all cell types. When we excluded SMCs from the common DEG population, we observed a similar number of DEGs in all cell types as in humans previously (Suppl. Fig. 5B).

Further, we analyzed the highest upregulated genes per cell type in humans and mice along with the regulation of that gene in the other species. Hereby, we observed how the genes with the largest changes in human heart failure patients behave in the respective mouse model (Suppl. Fig. 3).

We observed that the expression of the most regulated genes in human cell types is much less regulated in the mouse models. For example, we found *LDB2,* a gene of the LIM-Domain family, in human CMs as highly upregulated (Log2FC = 2.15) (Suppl. Fig. 3A). The LIM-Domain family genes are well known as adapter molecules that allow the assembly of transcriptional regulatory complexes in CM. However, in mice, *LDB2* is only mildly regulated upon HFrEF (Log2FC = 0.38). Other genes such as the VEGF receptor *FLT1,* which is upregulated in human cardiomyocytes, show a downregulation in mice CMs. This demonstrates that some genes have completely different expression patterns in humans and mice. However, some genes share similar regulation in their respective cell types. Thus, we observed that Phosphodiesterase 4D (*PDE4D)* and ADP Ribosylation Factor Like GTPase 15 (*ARL15)* show similar changes in ECs. Among the ten most upregulated genes in the mouse model data, we found three genes that also show a significant increase in their expression in humans (*RBPJ*, *SLC9A9*, *RUNX1*) (Suppl. Fig. 3B). The other genes, however, show little to no change. In contrast, if we investigate the expression changes in ECs, DEGs show an opposite direction in their expression change (*RBPJ*, *PID1*, *SLC9A9*). These differential gene expressions in the cell types suggest that some cell type specific responses may be different between human patients and mouse models.

Pathway enrichment results in cardiomyocytes

To address whether the relatively high number of significantly regulated genes indicate overall changes in pathways and pathological processes or whether the difference relates more to the alternative use of genes with similar functions in mice and humans, we further determined the implications for overall pathways in the individual celltypes. Figure 4 shows a *simplifyEnrichment* heatmap cluster with word clouds of gene ontology terms regulated in human or mouse cardiomyocytes. We generally observe more significantly enriched GSEA terms in humans than in mice (Fig 4). Important pathway terms regarding mitochondrial energy production and the electron chain were enriched in both species. Other terms involving developmental processes are enriched in humans compared to mice.

We also determined cell type specifically regulated pathways upon HFrEF. Therefore, we investigated how the enriched signaling pathways differ between humans and mice in cardiomyocytes. We observed larger differences for pathways that were specifically regulated in humans. Among the most regulated pathways, specifically detected in the human, we found the terms "actin filament organization" and "angiogenesis" (Fig. 5A). Genes associated with these pathways were explicitly upregulated in patients (Fig. 5B). These gene sets are not found among the regulated pathways in mice (Suppl. Table 5). Examples of angiogenesis-related genes, which are specifically induced in human heart failure but not in mouse models, including receptors such as the *VEGF*-receptor FLT1, or transcription factors like the mesenchyme homeobox protein 2 (MEOX2) (Figure 5B). In addition, many GTPase regulatory genes were found specifically increased in humans, including *MCF2L* and *RASGRF2*, which are known to regulate *RAC1*, and *SPATA13*, which enables guanyl-nucleotide exchange factor activity (32, 33). In contrast, we observed that signaling pathways mainly dealing with energy metabolism are commonly regulated in patients with heart disease as well as in mouse models. The genes included in pathways, such as “ATP biosynthetic process”, “mitochondrial ATP synthesis”, “aerobic electron transport chain” and “cellular respiration”, show significant downregulation compared to their corresponding control (Fig. 5C). These data suggest

conservation of disturbed mitochondrial metabolism in both mice and humans upon heart failure.

On the other hand, pathways such as "Wnt signaling pathway", "actin-myosin filament sliding" and “regulation of cell morphogenesis“ are upregulated specifically in the mouse HFrEF model (Fig. 5A). Genes associated with Wnt signaling include *LRP6*, a known inhibitor of cardiomyocyte proliferation (34), and the serine/threonine-protein kinase *MARK2*, which regulates the stability of microtubules through phosphorylation and inactivation of several microtubule-associated proteins (34).

Furthermore, we repeated the GSEA analysis with the identified ECs in the human and mouse model data to gain further insight into the different cell types (Suppl. Fig. 6). Here, we found human-specific regulated terms such as "cardiac contraction" and "regulation of axonogenesis" (Suppl. Fig. 6A) that we only find in ECs but not in the previously analyzed CMs. The genes in these sets show a distinct regulation only observed in human data (Suppl. Fig. 6B). When we examined the commonly regulated metabolic pathways. We found similar terms and changes in gene expression related to impaired mitochondrial metabolism in EC as we had previously observed in CMs (Suppl. Fig. 6C). In ECs, we also found similar mouse-specific terms such as "cell morphogenesis" and the "Wnt signaling pathway", but also newly discovered pathways such as "positive regulation of steroid hormone secretion". Steroid hormones have been shown to coordinate microvascular function in obese mice endothelium (35). Based on these results, one might speculate that this regulatory function is mouse specific.

Discussion

The ever growing number of published single cell experiments enables scientists to deepen the knowledge about transcriptional changes of individual cell types and species specific regulatory changes, upon disease conditions. Particular combination of single cell datasets

from different species in the same UMAP projection allows the detection of well conserved or species specific regulatory networks (36–38).

Therefore, integrating datasets from different species with a well curated list of orthologues, has significant advantages and simplifies comparisons among species.

Here we propose *OrthoIntegrate,* an R-package that enables scientists to integrate single cell datasets from different species into a shared dimensional space. To generate high quality and uniquely mapped orthologous lists between different species, we implemented a new pipeline that increases the ono-to-one assignment of ontologies to improve single cell integration. Compared to the Ensembl orthologous list (Biomart), our pipeline results in up to 10% more uniquely assigned orthologues between human and mouse.

OrthoIntegrate additionally contains functions that use the extended orthologous assignments to streamline the integration of single cell datasets from humans and mice. Moreover, it is highly adaptable and can be easily customized to support other species.

We demonstrated the usability of combining cross-species single cell datasets on a heart failure dataset with a reduced ejection fraction of humans and mice. Hereby, we could show major differences in the cell type expression patterns, which are differentially regulated in humans or mice upon HFrEF. Yet commonly regulated pathways also reflect an evolutionary conserved transcriptomic answer to severe damage in heart cells. One example is the conserved downregulation of critical mitochondrial metabolic pathways, which provide ATP for the heart (Fig. 4, Fig. 5A/C). The heart is the most energy consuming organ, so maintaining mitochondrial function plays a critical role and the decline in energy production limits heart function (39). Here we could show that genes important for ATP biosynthesis and electron transport (e.g. *PGAM2*, *NDUFA1* and *TMEM126A*) are consistently downregulated in heart failure. *Pgam2* overexpression has been shown to reduce heart stress resistance in mice (40). However, its role in humans has not been described so far. Also *NDUFA1*, a subunit of complex I, has been shown to be downregulated by about 50% in LAD rats, by western blot and mass spectrometry (41).

Besides commonly regulated pathways we found interesting differences between humans and mice upon heart failure. In cardiomyocytes, many genes associated with “angiogenesis” were increased in humans but not in mice. For example, the *VEGF* receptor *FLT1* was significantly augmented in the human samples. *FLT1* primarily mediates VEGF signaling in endothelial cells, but its role in cardiomyocytes is less clear (42). *FLT1* protein is well detected in cardiomyocytes in human cardiac tissue (43). Functionally, *FLT1* was shown to partially mediates *VEGF* induced cardiomyocyte differentiation of embryonic stem cells (44) and mediates *VEGF* induced cardiomyocyte calcium signaling and contractility in the embryonic zebrafish heart (45). Cardiomyocyte specific deletion of *Flt-1* was shown to worsen cardiac remodeling and hypertrophy induced by pressure overload (46), suggesting that the upregulation of its expression in humans may represent a compensatory cardioprotective mechanism that might not be conserved in mice.

A second example of genes specifically regulated in human CMs is *MEOX2*, also known as *GAX*, which was assigned the GO term “angiogenesis” because of its role in endothelial fatty acid transport (47). *MEOX2* plays a critical role in development of all muscle lineages (48). In cardiomyocytes, *MEOX2* overexpression blocks proliferation during heart morphogenesis causing proliferating cardiomyocytes to withdraw from the cell cycle (49). In addition, various guanine nucleotide exchange factors and regulators of Rho/Rac signaling pathways were shown to be specifically induced in human cardiomyocytes. While G protein-coupled signaling is well known to control cardiomyocytes (50), the function of the highly induced regulatory genes identified here (e.g. *MCF2L*, *RASGRF2*, and *SPATA13*) have not been studied in cardiomyocytes and their human specific regulation upon heart failure might be of utmost interest for future studies.

Among the pathways specifically enriched in mice we found predominant expression of genes associated with Wnt signaling. Although most identified genes have not been directly linked to cardiomyocyte-specific functions, Wnt signaling critically regulates cardiac hypertrophy,

remodeling and regeneration (34, 51). Therefore, these findings and the other identified species specific pathways deserve more in depth validation and investigation.

In summary, our publicly available bioinformatic tool *OrthoIntegrate* simplifies the comparison of scRNA-SEQ datasets from humans and mice. Thereby we could identify conserved regulatory pathways upon heart failure. Furthermore, we identified cell type specific differences in both species. Also, we showed pathways like angiogenesis regulated explicitly in humans, and Wnt signaling pathways, specifically regulated in mice.

We anticipate that this study shows the benefits of the joint analysis of scRNA-SEQ data through *OrthoIntegrate.* Due to the growing number of scRNA-SEQ datasets, we hope that *OrthoIntegrate* encourages other scientists to perform comparative analysis between different species and thereby increasing knowledge about conserved or species specific pathway responses in various diseases. This could improve the effective development of novel treatment strategies for heart failure or other diseases.

Limitations

The main limitation of our orthologue assignment and sample integration pipeline is the dependence on reliable databases for orthologous lists. Another problem with this approach is that it fails to consider the biological functions of the possible orthologues but selects the orthologue with highest sequence similarity. Second, our biological example has some limitations. While a decent number of healthy controls is available, the number of patients with HFrEF is limited. Knowing the biological heterogeneity of heart failure and comorbidities, variations are expected and the samples may not represent the representative and most common spectrum of heart failure. Finally, although the mouse model used is commonly applied in cardiovascular research, there are significant limitation due to the lack of underlying coronary artery disease and therapeutic pharmacological and interventions as it is done in humans. The integration of increasingly available published data both from alternative mice

models and data derived from human samples will allow a refined comparative analysis in the future.

Methods

Single cell pre-processing

Single-cell RNA-seq results were processed by CellRanger (10x Genomics) version 6.1.1 software. The first step consisted of demultiplexing and processing raw base count files by the implemented *mkfastq* tool. The human raw reads were mapped to the reference genome hg38 (GRCh38-2020) using Cellranger count, whereas the mouse raw reads were mapped to the reference genome mm10 (GRCm38-2020). The secondary data analysis was conducted using the Seurat 4.1.0 package in R. The data sets were first combined into a Seurat object and then subjected to a filtering process. Barcodes with too low (< 300) or too high number of genes (> 6000) were sorted out and not considered further in the data analysis. In addition, barcodes with too low (< 500) and too high read counts (> 15000) were also sorted out. To further ensure no apoptotic cells or doublets were analyzed, we discarded barcodes with a high percentage of mitochondrial content (> 5%). The filtered gene counts were then logarithmized and normalized according to the tutorial for data analysis with Seurat. Baseline characteristics for the samples can be found in Supplement Table 1.

Orthologue assignment and sample integration

In order to ensure the integration of single cell datasets from different species, we coded a function to assign mouse orthologues to the human nomenclature using gene transfer format (GTF) files provided by Ensembl (GRCh38, GRCm38). In order to detect only well annotated genes between the species, predicted genes were removed. Afterwards orthologues to the human genes were determined using the R package biomaRt. This assigned the majority of genes in our human GTF file to at least one orthologue. If there were several entries of possible

orthologues in the Ensembl database, a protein sequence comparison was initiated. Therefore, protein sequences were retrieved from the Uniprot database for the human gene and all possible mouse orthologues. These sequences were then aligned using the R package Biostrings 2.60.2. The alignment score was calculated based on the Needleman-Wunsch global alignment algorithm (28) with substitution matrices for nucleotide sequences or protein sequences. The mouse orthologue with the highest amino acid or nucleotide sequence similarity was assigned to the human orthologue. However, there were still Gene ID symbols which could not be uniquely assigned even using the Uniprot database. In order to be able to assign these as well, a further comparison with usage of the nucleotide sequence is initiated. For this purpose, the sequences for the human gene and for the possible mouse orthologues were obtained from the NCBI database and aligned analogously to the previous step and assigned to an orthologue with the highest alignment score. If all these assignment steps are not successful, the Levenshtein distance was used to compare the human ID symbol and the mouse ID symbols for possible orthologues and the orthologue with the lowest Levenshtein distance was selected.

Many long-non-coding RNAs are not listed in orthologue databases, therefore a final lowercase matching step was performed to assign genes like Malat1 to the human MALAT1. With this globally applicable list of orthologues between species, the datasets were now filtered by these and then merged into one object using Seurat's canonical correlation analysis (CCA) integration.

Clustering, silhouette coefficient and annotation

To classify cells into clusters based on their expressed genes, we used the *FindNeighbors* and *FindClusters* (resolution parameter = 0.3) function implemented in Seurat. These clusters are determined by applying the shared nearest neighbors (SNN) clustering algorithm and the Uniform Manifold Approximation and Projection (UMAP) dimension reduction.

Calculations of the silhouette coefficient are based on computing a distance matrix based on the cell embeddings matrix for principal component analysis (PCA) performed by Seurat. This

distance matrix includes the information of cell-cell distance, which is necessary for calculating the silhouette coefficient with our calculated clusters in the function *silhouette* of the *cluster* package (version 2.1.4). Additionally, the coefficients of the samples were averaged for each object. The orthologous lists for OMA, Biomart and InParanoid were created by following their introductions on their tool descriptions and by using the same GTF files as before (GRCh38, GRCm38).

For the assignment of cell clusters to cell types, we used a reference object that we had previously manually annotated with marker genes from Tombor et al. 2021 (52). Here, the R package SingleR can be used to adopt marker genes that were used for the previous annotation of clusters of the reference object. These are then transferred and compared to marker genes of the cell clusters of our object to be annotated. Thus, a reproducible annotation can be guaranteed with the help of an exactly annotated data set.

Differential gene expression analysis and gene ontology analysis

Detection of differentially regulated genes (DEG) for the cell type specific clusters was performed by the hurdle model of the MAST package (version 1.20.0). Results were filtered by their Bonferroni-adjusted p-value ($p.adj < 0.05$). The totality of DEGs were represented by Sankey plots created with the R package networkD3 (version 0.4). Additionally, bar plots were created using R package ggplot2, representing human DEGs and their regulation in mice. DEGs were also divided according to their species and cell type assignment and then visualized for DEGs with a positive Log2FC and separately in another plot, for DEGs with a negative Log2FC. Here, DEGs occurring in both human and mouse for the respective cell type have been pooled. Visualization was done in the form of a Circos plot (R package circlize 0.4.14). The gene regulation heatmap was created using the log2FC of all identified genes and a k-means clustering ($k = 40$) (R package ComplexHeatmap 2.16.0). Visualization of distinct and similar populations of genes in the analyzed cell types per species was achieved by creating venn diagrams with the Jvenn webtool.

Gene Set Enrichment Analysis (GSEA) was performed using the R package clusterProfiler (version 4.2.2) and the GO Database. GSEA terms were calculated separately for each cell type. The terms were sorted according to the Benjamini-Hochberg adjusted p.value and evaluated according to their “normalized enrichment distribution”, which gives information about the regulation of the genes in the described pathway. A heatmap was created by clustering the GSEA terms by their similar geneIDs. (R package simplifyEnrichment 1.10.0). Additionally, the GSEA results were plotted in dot plots. Specifically, for genes described in the pathway, the standard error of the mean (SEM) bar plot was created (for their averaged UMIs) by using the R package ggplot2.

Acknowledgment

The study was supported by grants from the German Centre for Cardiovascular Research (DZHK) to D.J. and S.D., the German Research Foundation (DFG; Exc2026/1) and the Dr. Rolf M. Schwiete Stiftung, Projekt 08/2018 to S.D.

Code availability

The *OrthoIntegrate* package containing the integration pipeline and the orthologue algorithm are available on Github (github.com/MarianoRuzJurado/OrthoIntegrate). Additionally, codes for R analysis and plots of data presented in this study are available on another GitHub repository (github.com/MarianoRuzJurado/RuzJurado_et_al_2023).

Data availability

The single nuclei data for human have been deposited in the Human Cell Atlas (HLC) database and can be accessed through the HCA Data Portal. The mice sequencing data are available through ArrayExpress under the accession number E-MTAB-7869.

Ethics declaration

The authors declare no competing interests.

References

1. A. C. Ericsson, M. J. Crim, C. L. Franklin, A brief history of animal modeling. *Mo. Med.* **110**, 201–205 (2013).
2. R. J. Wall, M. Shani, Are animal models as good as we think? *Theriogenology* **69**, 2–9 (2008).
3. N. Shanks, R. Greek, J. Greek, Are animal models predictive for humans? *Philos. Ethics Humanit. Med.* **4**, 2 (2009).
4. E. W. Uhl, N. J. Warner, Mouse Models as Predictors of Human Responses: Evolutionary Medicine. *Curr. Pathobiol. Rep.* **3**, 219–223 (2015).
5. , *The Mouse in Biomedical Research: History, Wild Mice, and Genetics* (Elsevier, 2006).
6. C. Riehle, J. Bauersachs, Small animal models of heart failure. *Cardiovasc. Res.* **115**, 1838–1849 (2019).
7. Mouse Genome Sequencing Consortium, *et al.*, Initial sequencing and comparative analysis of the mouse genome. *Nature* **420**, 520–562 (2002).
8. C. J. Bult, *et al.*, Mouse Genome Database (MGD) 2019. *Nucleic Acids Res.* **47**, D801–D806 (2019).
9. N. A. Noll, H. Lal, W. D. Merryman, Mouse Models of Heart Failure with Preserved or Reduced Ejection Fraction. *Am. J. Pathol.* **190**, 1596–1608 (2020).
10. A. Breschi, T. R. Gingeras, R. Guigó, Comparative transcriptomics in human and mouse. *Nat. Rev. Genet.* **18**, 425–440 (2017).
11. N. R. Dayeh, *et al.*, Echocardiographic validation of pulmonary hypertension due to heart failure with reduced ejection fraction in mice. *Sci. Rep.* **8**, 1363 (2018).
12. S. Sawall, *et al.*, In Vivo Quantification of Myocardial Infarction in Mice Using Micro-CT and a Novel Blood Pool Agent. *Contrast Media Mol. Imaging* **2017**, 2617047 (2017).
13. E. Van Craeyveld, F. Jacobs, S. C. Gordts, B. De Geest, Low-density lipoprotein receptor gene transfer in hypercholesterolemic mice improves cardiac function after myocardial infarction. *Gene Ther.* **19**, 860–871 (2012).
14. B. Swynghedauw, Molecular mechanisms of myocardial remodeling. *Physiol. Rev.* **79**, 215–262 (1999).
15. G. Ertl, S. Frantz, Healing after myocardial infarction. *Cardiovasc. Res.* **66**, 22–32

(2005).

16. R. Vigen, T. M. Maddox, L. A. Allen, Aging of the United States population: impact on heart failure. *Curr. Heart Fail. Rep.* **9**, 369–374 (2012).

17. D. Jovic, *et al.*, Single-cell RNA sequencing technologies and applications: A brief overview. *Clin. Transl. Med.* **12**, e694 (2022).

18. T. Stuart, *et al.*, Comprehensive Integration of Single-Cell Data. *Cell* **177**, 1888–1902.e21 (2019).

19. B. Hwang, J. H. Lee, D. Bang, Single-cell RNA sequencing technologies and bioinformatics pipelines. *Exp. Mol. Med.* **50**, 1–14 (2018).

20. Y. Lu, R. Rosenfeld, G. J. Nau, Z. Bar-Joseph, Cross species expression analysis of innate immune response. *J. Comput. Biol.* **17**, 253–268 (2010).

21. E. Kristiansson, *et al.*, A novel method for cross-species gene expression analysis. *BMC Bioinformatics* **14**, 70 (2013).

22. J. Seok, *et al.*, Genomic responses in mouse models poorly mimic human inflammatory diseases. *Proc. Natl. Acad. Sci. U. S. A.* **110**, 3507–3512 (2013).

23. S. Parekh, C. Ziegenhain, B. Vieth, W. Enard, I. Hellmann, zUMIs - A fast and flexible pipeline to process RNA sequencing data with UMIs. *Gigascience* **7** (2018).

24. I. Korsunsky, *et al.*, Fast, sensitive and accurate integration of single-cell data with Harmony. *Nat. Methods* **16**, 1289–1296 (2019).

25. F. Cunningham, *et al.*, Ensembl 2022. *Nucleic Acids Res.* **50**, D988–D995 (2022).

26. E. W. Sayers, *et al.*, Database resources of the national center for biotechnology information. *Nucleic Acids Res.* **50**, D20–D26 (2022).

27. UniProt Consortium, UniProt: the universal protein knowledgebase in 2021. *Nucleic Acids Res.* **49**, D480–D489 (2021).

28. S. B. Needleman, C. D. Wunsch, A general method applicable to the search for similarities in the amino acid sequence of two proteins. *J. Mol. Biol.* **48**, 443–453 (1970).

29. A. M. Altenhoff, *et al.*, OMA orthology in 2021: website overhaul, conserved isoforms, ancestral gene order and more. *Nucleic Acids Res.* **49**, D373–D379 (2021).

30. D. Smedley, *et al.*, BioMart--biological queries made easy. *BMC Genomics* **10**, 22 (2009).

31. K. P. O'Brien, M. Remm, E. L. L. Sonnhammer, Inparanoid: a comprehensive database of eukaryotic orthologs. *Nucleic Acids Res.* **33**, D476–80 (2005).

32. Y. Kawasaki, *et al.*, Identification and characterization of Asef2, a guanine-nucleotide exchange factor specific for Rac1 and Cdc42. *Oncogene* **26**, 7620–7267 (2007).

33. S.-C. Huang, *et al.*, Downregulation of MCF2L Promoted the Ferroptosis of Hepatocellular Carcinoma Cells through PI3K/mTOR Pathway in a RhoA/Rac1 Dependent Manner. *Dis. Markers* **2022**, 6138941 (2022).

34. Y. Wu, *et al.*, LRP6 downregulation promotes cardiomyocyte proliferation and heart

regeneration. *Cell Res.* **31**, 450–462 (2021).

35. L. A. Biwer, B. V. Carvajal, Q. Lu, J. J. Man, I. Z. Jaffe, Mineralocorticoid and Estrogen Receptors in Endothelial Cells Coordinately Regulate Microvascular Function in Obese Female Mice. *Hypertension* **77**, 2117–2126 (2021).

36. S. Balachandran, J. Pozojevic, V. K. A. Sreenivasan, M. Spielmann, Comparative single-cell analysis of the adult heart and coronary vasculature. *Mamm. Genome* (2022) https:/doi.org/10.1007/s00335-022-09968-7.

37. A. Butler, P. Hoffman, P. Smibert, E. Papalexi, R. Satija, Integrating single-cell transcriptomic data across different conditions, technologies, and species. *Nat. Biotechnol.* **36**, 411–420 (2018).

38. M. Baron, *et al.*, A Single-Cell Transcriptomic Map of the Human and Mouse Pancreas Reveals Inter- and Intra-cell Population Structure. *Cell Syst* **3**, 346–360.e4 (2016).

39. J. M. Huss, D. P. Kelly, Mitochondrial energy metabolism in heart failure: a question of balance. *J. Clin. Invest.* **115**, 547–555 (2005).

40. J. Okuda, *et al.*, Persistent overexpression of phosphoglycerate mutase, a glycolytic enzyme, modifies energy metabolism and reduces stress resistance of heart in mice. *PLoS One* **8**, e72173 (2013).

41. T. Liu, *et al.*, Mitochondrial proteome remodeling in ischemic heart failure. *Life Sci.* **101**, 27–36 (2014).

42. S. Kurotsu, *et al.*, Distinct expression patterns of Flk1 and Flt1 in the coronary vascular system during development and after myocardial infarction. *Biochem. Biophys. Res. Commun.* **495**, 884–891 (2018).

43. M. Karlsson, *et al.*, A single-cell type transcriptomics map of human tissues. *Sci Adv* **7** (2021).

44. Y. Chen, *et al.*, Vascular endothelial growth factor promotes cardiomyocyte differentiation of embryonic stem cells. *Am. J. Physiol. Heart Circ. Physiol.* **291**, H1653–8 (2006).

45. W. Rottbauer, *et al.*, VEGF-PLCgamma1 pathway controls cardiac contractility in the embryonic heart. *Genes Dev.* **19**, 1624–1634 (2005).

46. L. Mei, *et al.*, Increased cardiac remodeling in cardiac-specific Flt-1 receptor knockout mice with pressure overload. *Cell Tissue Res.* **362**, 389–398 (2015).

47. G. Coppiello, *et al.*, Meox2/Tcf15 heterodimers program the heart capillary endothelium for cardiac fatty acid uptake. *Circulation* **131**, 815–826 (2015).

48. H. A. Skopicki, *et al.*, Embryonic expression of the Gax homeodomain protein in cardiac, smooth, and skeletal muscle. *Circ. Res.* **80**, 452–462 (1997).

49. S. A. Fisher, E. Siwik, D. Branellec, K. Walsh, M. Watanabe, Forced expression of the homeodomain protein Gax inhibits cardiomyocyte proliferation and perturbs heart morphogenesis. *Development* **124**, 4405–4413 (1997).

50. J. H. Brown, D. P. Del Re, M. A. Sussman, The Rac and Rho hall of fame: a decade of hypertrophic signaling hits. *Circ. Res.* **98**, 730–742 (2006).

51. M. W. Bergmann, WNT signaling in adult cardiac hypertrophy and remodeling: lessons learned from cardiac development. *Circ. Res.* **107**, 1198–1208 (2010).

52. L. S. Tombor, *et al.*, Single cell sequencing reveals endothelial plasticity with transient mesenchymal activation after myocardial infarction. *Nat. Commun.* **12**, 681 (2021).

Figure Legends

Fig.1: Integrated human and mouse snRNA-SEQ data of healthy and heart failure samples.

(A) Use case diagram of *OrthoIntegrate*: Shown are the steps that are run by the user within their standard Seurat workflow. First the Import function is used to create Seurat objects from scRNA-seq data, second orthologues are searched by the BuildOrtholog function and the third step creates an integrated object with uniform nomenclature by using the IntegrateObjects. (B) UMAP showing human cells (red) and mice cells (blue) in a common UMAP projection. In addition, cell types for the cell clusters can be seen. (C) UMAP with defined clusters according to Seurat's clustering, divided by species. Cells of mouse and human origin commingled in all clusters. There are no clusters formed that originated from only one of the two species. The cells were identified as cardiomyocytes (red), fibroblasts (yellow), endothelial cells (green), pericytes (turquoise), immune cells (blue), smooth muscle cells (purple) and neuronal cells (pink). (D) Bar plot showing cell composition of cell types in human (red) and mice (blue) samples. Samples were grouped based on their origin into human controls from the left ventricle (Human-CTRLlv), human HFrEF (Human-HFrEF), mouse controls (Mice-CTRL), and mouse HFrEF model (Mice-HFrEF). Cell types were then analyzed for their composition from the previously mentioned groups and plotted. P-values above the certain groups were calculated by two-sided Student's t-test. (E) Dot plot depicting the average expression levels and expression proportions in human samples of the top ten feature genes for the found cell types. The size of the dot represents the proportion of cells expressing the indicated gene within a cell type, and the color indicates the average expression level of cells. (F) Dot plot depicting the average expression levels and expression proportions in mice samples of the top ten feature genes for the found cell types. Similar to (E) the size of the dot represents the proportion of cells expressing the indicated gene within a cell type, and the color indicates the average expression level of cells.

Fig.2: DEG analysis shows similar and different expressed DEGs.

(A) Sankey plot illustrating the distribution of differentially regulated genes (DEG) in the corresponding cell types. The width of the paths illustrates the number of DEGs that are either human specific (yellow), detected in both species (light green) or mouse-specific (dark green). DEG analysis was performed for each cell type individually. Neuronal cells were omitted from all further analyses due to their insufficient number of cells in the mouse data. (B) Bar graph of up (top) and down (bottom) regulated genes in humans, along with the expression in mice. The panels show genes that are either commonly regulated (left), regulated in humans and not regulated in mice (middle) and regulated in opposite directions.

Fig.3: DEG analysis shows similar and different populations of regulation in gene expression patterns upon heart failure in humans and mice. (A) Heatmap of log2FC values (Control vs HFrEF) for all genes and all cell types. The y-axis describes all genes (16.545) clustered by a k-means algorithm (k = 40). The x-axis shows the species and the additional clustering into the different cell types. Positive log2FCs are represented by a red color, while negative scores give a blue color. (B) Close-up of the 24-40 k-means clusters of log2FCs of genes in which most cell type-specific differences are observed. (C) Venn Diagrams of all identified DEGs in human (top) and mouse (bottom) (log2FC > 0.1 and p-adjusted < 0.05).

Fig.4: GSEA analysis reveals more regulated pathways in heart failure in human cardiomyocytes than in mice, with the terms found sharing many keywords. Heatmap clustering significant GSEA results (p.adj < 0.25) of DEGs found in human and mouse cardiomyocytes by similar GeneIDs in the pathways. Bar graphs are shown on the left y-axis representing the number of pathways found in the respective cluster for the given species and condition. In addition, the adjusted p-value is color-coded from 1 (green) to the smallest p-value found ~0.025 (red). On the right side of the y-axis keywords describing the found

pathways in that cluster are shown, where the size of the word represents its frequency in the terms (larger = most, smaller = less).

Fig.5: Common and distinct regulated pathways found in human and mouse cardiomyocytes.

(A) Dot plot visualizing the ten most significant pathways for terms only to be found regulated in humans, commonly regulated and specific in mice. The size of the dots corresponds to the negative log10 of the Benjamini Hochberg adjusted p-value and the color-code represents the normalized enrichment score (NES), with upregulated pathways shown in red and downregulated pathways in blue. The y-axis depicts the description of the identified term.

(B) Bar plot with mean values for the amount of unique molecular identifiers (UMIs) in the cells for the shown genes. The genes are identified to be dissimilarly regulated between humans and mice for pathways specifically found in humans. (C) Bar graph similar to (B) with mean values for UMIs in cells for genes downregulated in both species for commonly found terms. (D) Bar graph similar to (B) and (C) with mean values for UMIs in cells for genes which are uniquely found to be regulated in terms specifically identified in mice. P-values above the certain groups were calculated by two-sided Student's t-test.

Click here to access/download;Figure;Fig_1.pdf

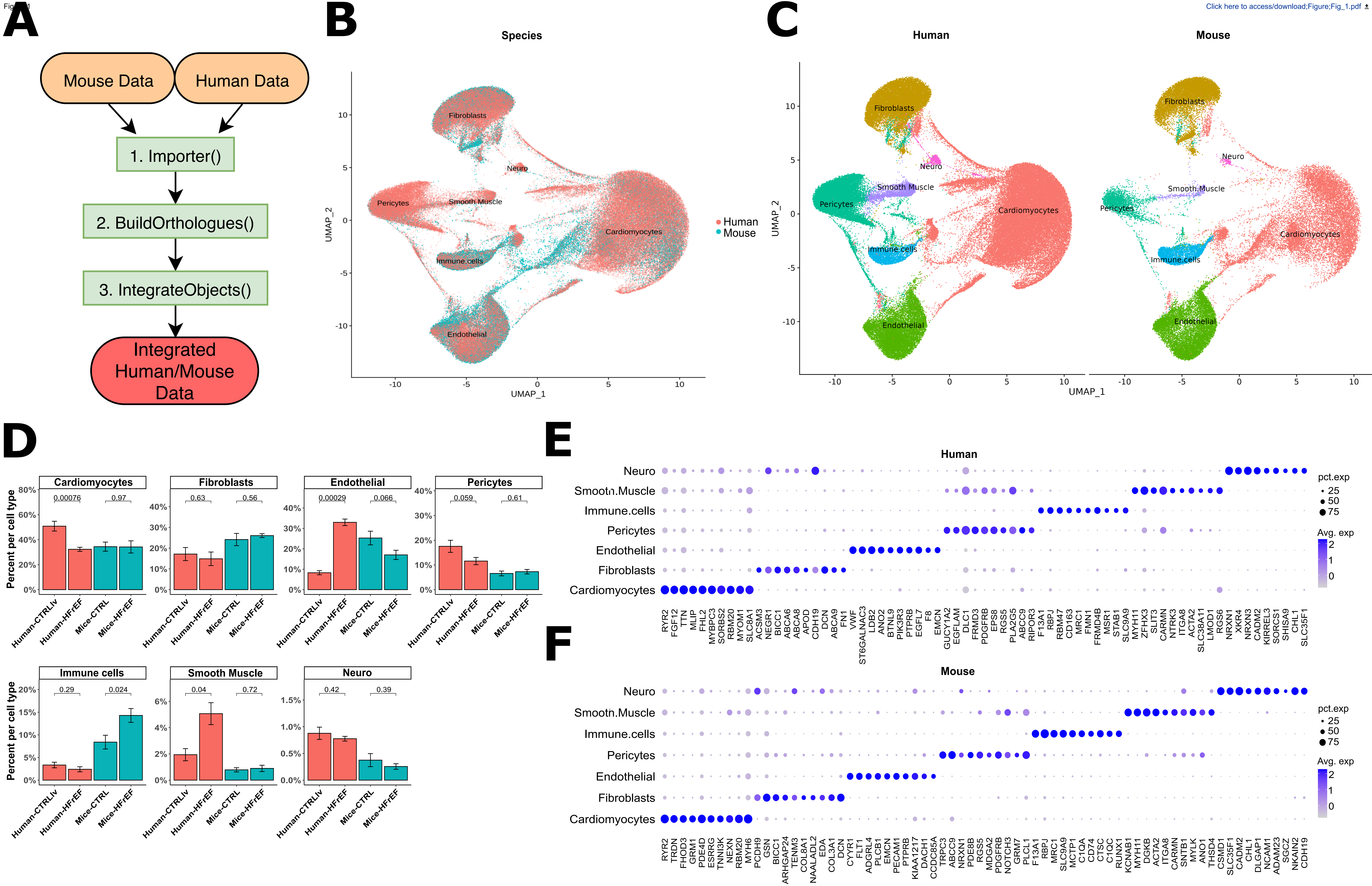

Click here to access/download;Figure;Fig_2_new.pdf

Figure 2

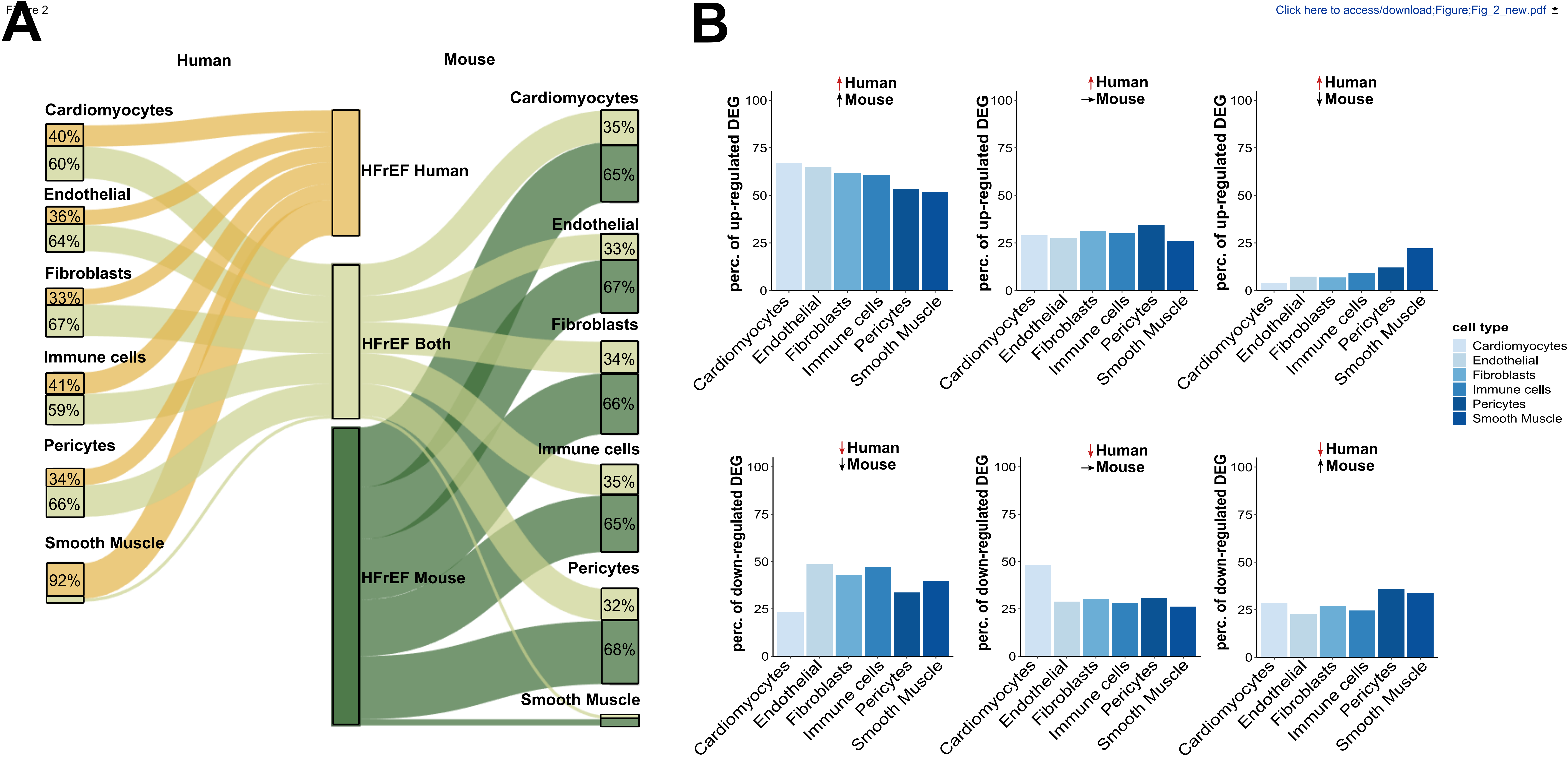

Figure 3

Click here to access/download;Figure;Fig_3_new.pdf

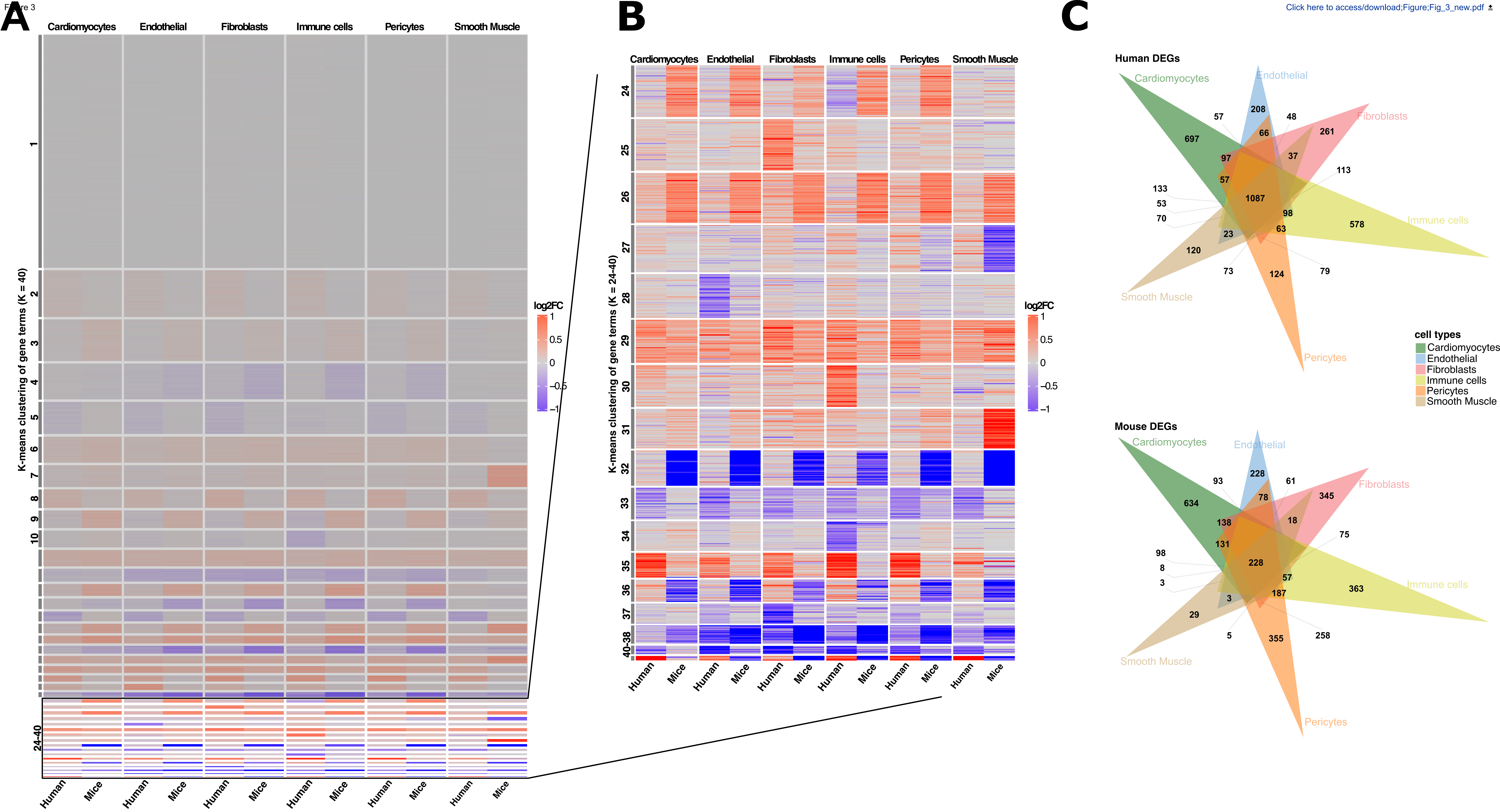

Figure 4

Click here to access/download;Figure;Fig_4.pdf

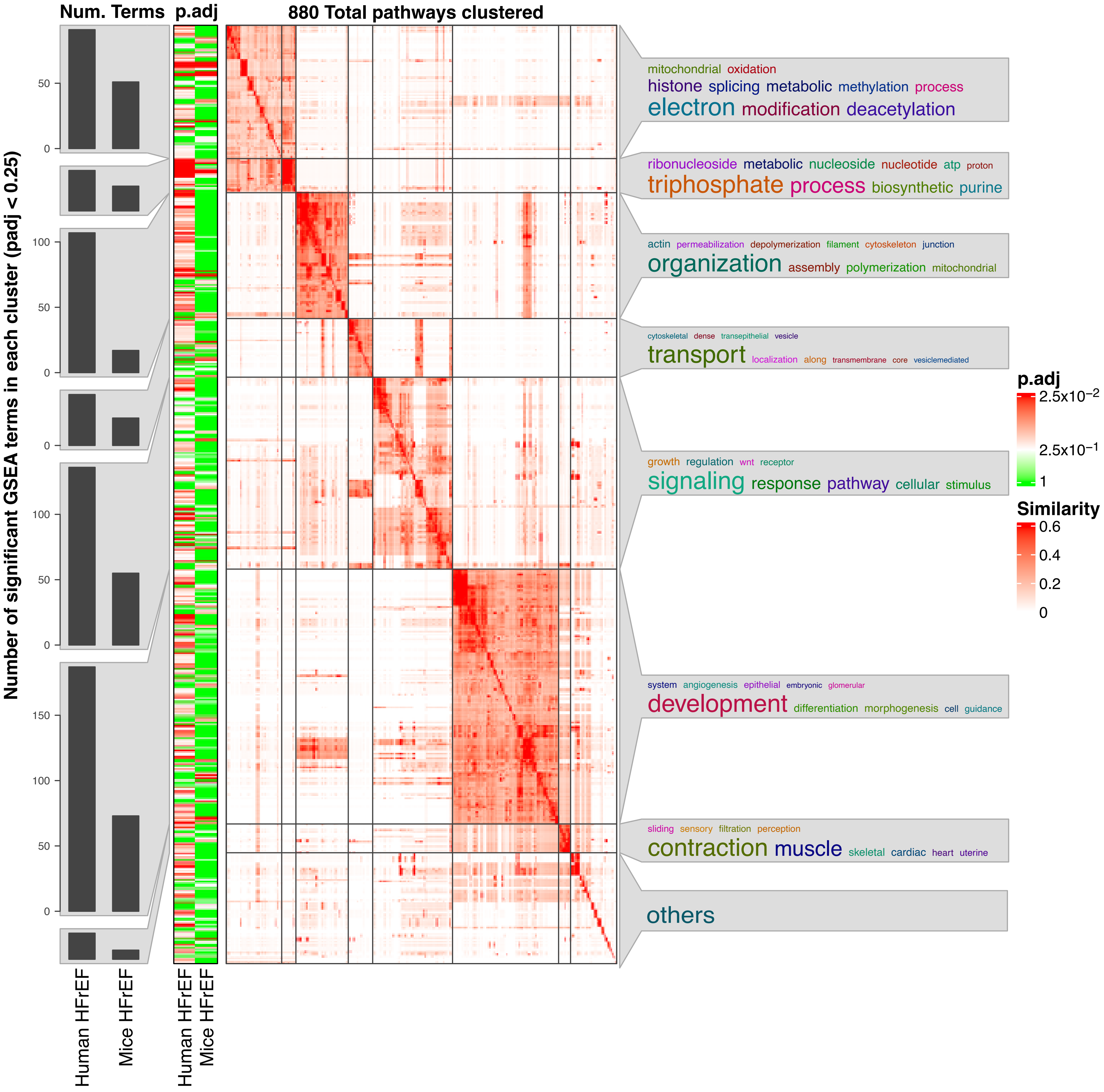

Figure 5

Click here to access/download;Figure;Fig_5.pdf

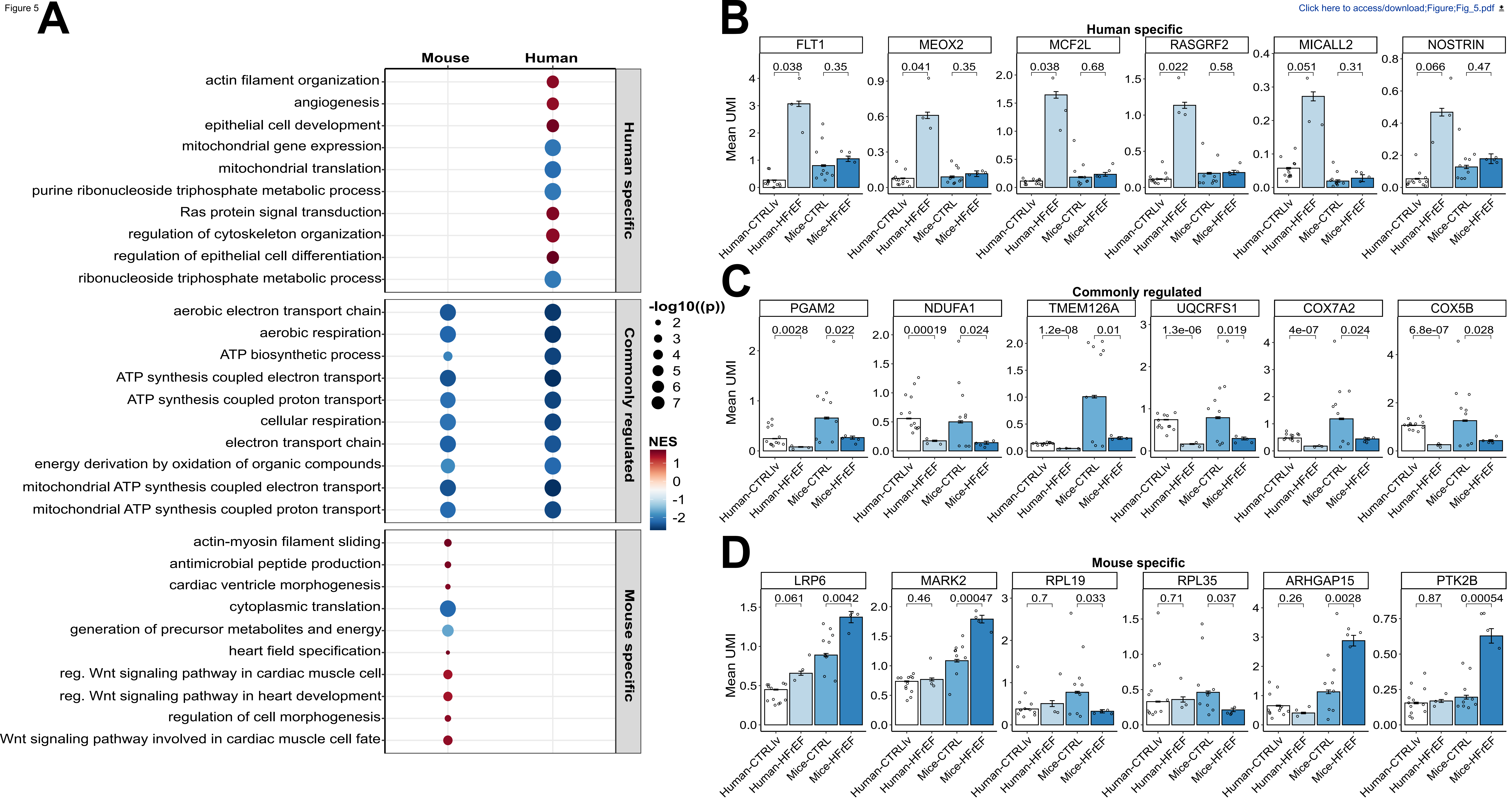

Supplementary Figure 1

Click here to access/download
Supplementary Material
Supp_Fig_1_pipeline.pdf

Supplementary Figure 2

Click here to access/download
Supplementary Material
Supp_Fig_2_UMAPs_species.pdf

Supplementary Figure 3

Click here to access/download
Supplementary Material
Supp_Fig_3_circos.pdf

Supplementary Figure 4

Click here to access/download

Supplementary Material

Supp_Fig_4_Silhouette.pdf

Supplementary Figure 5

Click here to access/download
Supplementary Material
Supp_Fig_5_Upset_DEG.pdf

Supplementary Figure 6

Click here to access/download
Supplementary Material
Supp_Fig_6_GSEA_Endothelial.pdf

Supplementary Table 1

Click here to access/download
Supplementary Material
Supp_1_PAPER_baseline_charac_Human_Mice.xlsx

Supplementary Table 2

Click here to access/download
Supplementary Material
Supp_2_OrthologueAssignment_stats_mice.xlsx

Supplementary Table 3

Click here to access/download

Supplementary Material

Supp_3_silhouette DataFrame per Sample.xlsx

Supplementary Table 4

Click here to access/download
Supplementary Material
Supp_4_Human Mice DEG analysis HFrEF CTRL.xlsx

Supplementary Table 5

Click here to access/download
Supplementary Material
Supp_5_Human Mice HFrEF GSEA CM.xlsx

Supplementary Table 6

Click here to access/download

Supplementary Material

Supp_6_Human Mice HFrEF GSEA EC.xlsx.xlsx

Click here to access/download
Supplementary Material
Supp_1_baseline_charac_Human_Mice.xlsx
