# Supplementary material for: Improved integration of single-cell transcriptome data demonstrates common and unique signatures of heart failure in mice and humans
Source: Gigascience. 2024 Apr 4;13:giae011. doi: 10.1093/gigascience/giae011 (PMC10993718; doi:10.1093/gigascience/giae011)
Supplement: giae011_GIGA-D-23-00222_Revision_1 [file giae011_giga-d-23-00222_revision_1.pdf]

GigaScience

Improved integration of single cell transcriptome data demonstrates common and unique signatures of heart failure in mice and humans

--Manuscript Draft--

**Manuscript Number:**	GIGA-D-23-00222R1
**Full Title:**	Improved integration of single cell transcriptome data demonstrates common and unique signatures of heart failure in mice and humans
**Article Type:**	Research
**Funding Information:**	Deutsche Forschungsgemeinschaft (DFG) (Exc2026/1) — Dr. David John<br>Dr. Rolf M. Schwiete Stiftung (Projekt 08/2018) — Prof. Dr. Stefanie Dimmeler
**Abstract:**	Background<br><br>Cardiovascular research heavily relies on mouse (mus musculus) models to study disease mechanisms and to test novel biomarkers and medications. Yet, applying these results to patients remains a major challenge and often results in non-effective drugs. Therefore, it is an open challenge of translational science to develop models with high similarities and predictive value. This requires a comparison of disease models in mice with diseased tissue derived from humans.<br><br>Results<br><br>To compare the transcriptional signatures at single cell resolution, we implemented an integration pipeline called OrthoIntegrate which uniquely assigns orthologs and therewith merges single cell data (scRNA-SEQ) of different species. The pipeline has been designed to be as easy to use and is fully integrable in the standard Seurat workflow.<br>We applied OrthoIntegrate on scRNA-SEQ from cardiac tissue of heart failure patients with reduced ejection fraction (HFrEF) and scRNA-SEQ from the mice after chronic infarction, which is a commonly used mouse model to mimic HFrEF. We discovered shared and distinct regulatory pathways between human HFrEF patients and the corresponding mouse model. Overall, 54% of genes were commonly regulated including major changes in cardiomyocyte energy metabolism. However, several regulatory pathways, e.g. angiogenesis, were specifically regulated in humans.<br><br>Conclusion<br><br>The demonstration of unique pathways occurring in humans indicate limitations on the comparability between mice models and human HFrEF and show that results from the mice model should be validated carefully. OrthoIntegrate is publicly accessible (https://github.com/MarianoRuzJurado/OrthoIntegrate) and can be used to integrate other large data sets to provide a general comparison of models with patients data.
**Corresponding Author:**	David John, Ph-D<br>Goethe-Universitat Frankfurt am Main<br>Frankfurt am Main, Hessen GERMANY
**Corresponding Author Secondary Information:**	
**Corresponding Author's Institution:**	Goethe-Universitat Frankfurt am Main
**Corresponding Author's Secondary Institution:**	
**First Author:**	Mariano Ruz Jurado
**First Author Secondary Information:**	
**Order of Authors:**	Mariano Ruz Jurado

Powered by Editorial Manager® and ProduXion Manager® from Aries Systems Corporation

	Lukas S. Tombor
	Mani Arsalan
	Tomas Holubec
	Fabian Emrich
	Thomas Walther
	Wesley Abplanalp
	Ariane Fischer
	Andreas M. Zeiher
	Marcel H. Schulz
	Stefanie Dimmeler
	David John, Ph-D
**Order of Authors Secondary Information:**	
**Response to Reviewers:**	Response to the reviewers

In general, we think that the review process strongly improved the paper quality and therefore we would like to thank the reviewers for their remarks and suggestions. Te answers to the reviewer questions and remarks can be found below the comment, marked in green.

Reviewer1 :

A few points need to be addressed before publishing

1. The authors utilized the Needleman-Wunsch algorithm to generate one-to-one orthologs between human genes and mouse genes. What is the advantage of using this algorithm compared to other algorithms i.e., SAMap uses BLAST?

We appreciate the reviewer's comment. In our study, we focused on aligning sequences with multiple orthologues listed for one human orthologue in the ensemble database. To ensure accurate comparisons, we opted for global alignments since the reference and query sequences have similar lengths. From a small gene set of potential homologues, our aim was to select the orthologue with the highest overall agreement with the reference sequence. Global alignments were chosen because they are less likely to show false homology [1].
On the other hand, local alignments, as employed in SaMap (Blast), have advantages in identifying homologues in species without well-annotated genomes or between evolutionarily distant species [2]. Their purpose is to pinpoint similar sequencing regions within larger sequences. However, they cannot consider the overall sequence conservation of sequences with similar lengths [1].
SaMap is specifically designed for defining homologs between species with higher evolutionary distances, but it comes with computational expenses [3]. Our study focused on comparing well-annotated species with high evolutionary conservation, and we achieved the best results with the global alignment strategy used in OrthoIntegrate. However in order to provide functionality for more distant species we added a parameter to select between global and local alignments during the assignment of one-to-many orthologues. The function BuildOrthologues() now provides an argument alignment_type, which can be set to local or global, and thereby switching between the Smith-Waterman and the Needleman-Wunsch algorithm.

2. The authors have shown the application of OrthoIntegrate in the context of heart failure between mice and humans. Could the authors include at least one more example of using OrthoIntegrate in other disease conditions or between other species to show the versatility of OrthoIntegrate?

We thank the reviewer for this reasonable comment. As suggested, we applied OrthoIntegrate to another species and another disease condition. Therefore, we obtained single cell data from human mouse and zebrafish for healthy and Alzheimer's conditions and integrated it with Ortho Integrate. The results are summarized in Suppl.

Fig. 6 and an additional paragraph was added to the discussion.

“To further demonstrate the functionality of OrthoIntegrate, we integrated scRNA-SEQ data from human [41], mouse [54] and zebrafish [54,55] brain tissue under alzheimer condition. Besides the evolutionary distance between these species, we could jointly cluster different cell types via OrthoIntegrate (Suppl. Fig 6 A-C) and detect commonly expressed marker genes within these cell clusters (Suppl. Fig 6 D-F).”

3. To assess the quality of clustering after integration, the authors calculated silhouette coefficients/scores and found that integration by OrthoIntegrate resulted in an improved clustering performance. Could the authors include more benchmarking metrics to assess the performance of OrthoIntegrate compared to other methods? The authors could consider metrics like the species mixing score used by BENGAL (Song et al., 2022, biorxiv; https://github.com/Functional-Genomics/BENGAL)

We would like to thank the reviewer for drawing our attention to the Bengal Paper [3] . We applied all their benchmarking metrics to our pipeline as well. The results can be seen in Figure 2D. Additionally we added the following paragraph to the discussion section.

“We demonstrated the usability of combining cross-species single cell data by using data sets of human and mouse heart failure with reduced ejection fraction.
In order to evaluate the species mixing and the biological conservation of different integration methods, we applied certain metrics from the scib package [4,5], which were also suggested by Song et.al [3–5]. The results are summarized in Figure 2D. We found that most batch correction scores improve by using OrthoIntegrate.
For biological conservation scores, we demonstrate that some metrics, like the “cell cycle conservation” are improved by using OrthoIntegrate. Which means that the variance caused by different cell cycle states of the cells is conserved via OrthoIntegrate. Other parameters like the NMI-score are reduced. But this score for example is strongly influenced by the cell type labeling [3], which was focused only on main cell type groups in these datasets, regardless of the existence of subpopulation or mixed cell type population clusters. In other words, subclusters of different cell types were not annotated in detail. Due to the increased numbers of features that are included in OrthoIntegrate, the clustering might be more diverged, likely by species specific non-coding RNAs or other features, which are not included in the other databases. Therefore, the more divergent clustering, due the increased number of features in OrthoIntegrate combined with the broad cell type labeling might explain the slightly reduced NMI scores. “

4. Miscalling of figures: silhouette coefficients are shown in Supp_Fig_4 rather than Suppl_Fig_3.

We changed the text accordingly and added Supp_Fig_4 to the main manuscript as Fig 2.

5. Some information on the used datasets in the manuscript has been shown in supplementary table 1, but it's still a bit confusing, for example, where the mouse and human HFrEF datasets come from. I am not exactly sure, but I presume HFrEF datasets are from E-MTAB-13264? This information should be described more explicitly in the method section.

We changed the text accordingly and added detailed information regarding the origin of the samples. Additionally, we added a paragraph to describe the source of the Alzheimer datasets.

Reviewer2:

- [ ] 1. Ortholog identification has long been a critical and essential step for many comparative, evolutionary, and functional genomic analyses. To evaluate the performance of an orthology inference method, there are some gold standards

Powered by Editorial Manager® and ProduXion Manager® from Aries Systems Corporation

available for benchmark testing, such as the Quest Orthology Benchmark Service (https://orthology.benchmarkservice.org). Whether OrthoIntegrate outperforms other methods should be comprehensively benchmarked on diverse datasets and metrics, rather than relying solely on the silhouette coefficient score from a heart single-cell RNA sequencing (scRNA-seq) dataset.

According to the reviewer suggestions, we incorporated the Quest Orthology Benchmark Service and tested the 4 ortholog databases. We could show that OrthoIntegrate has the second highest Gene Ontology Conservation score and the second highest Enzyme Classification score. The highest score in these tests was achieved with InPara.
We tried to perform other tests as well. But since we are only comparing two species and these tests require multi species evolutionary trees, we could not perform all tests from the Quest Orthology Benchmark Service. The results from the Orthology Benchmark Service were incorporated in Fig. 2E.

- [ ] 2. According to the authors' integration pipeline, both human and mouse scRNA-seq data are individually clustered to assign cell type labels and are then further integrated with orthologous genes for clustering to assign new labels. How do the labels for each cell and each cell type change before and after the integration approach? Does cell type assignment become more reasonable after the integration? The authors should demonstrate that the selection of orthologous genes for clustering improves the accuracy of cell type assignment. The silhouette coefficient score is not a direct metric for assessing accuracy, as it can be influenced by biological factors. For example, in Supplementary Table 3, the silhouette scores of mouse-HFrEF samples generated by Paranoid and OMA are consistently higher than those by OrthoIntegrate, which is opposite to the control groups and human-HFrEF samples.

In order to assign cell type, we manually applied previously established marker genes (Tombor et al. 2021) to assign the human clusters accordingly. Afterwards we used singleR, to transfer cell labels from the human to the mouse samples, based on published marker genes.
Furthermore, we applied all benchmarking metrics from the BENGAL paper [3] to our pipeline in order to validate celltype assignment.

We added the following paragraph to the discussion:

“We demonstrated the usability of combining cross-species single cell data by using data sets of human and mouse heart failure with reduced ejection fraction.
In order to evaluate the species mixing and the biological conservation of different integration methods, we applied certain metrics from the scib package [4,5], which were also suggested by Song et.al [3–5]. The results are summarized in Figure 3D. We found that most batch correction scores improve by using OrthoIntegrate.
For biological conservation scores, we demonstrate that some metrics, like the “cell cycle conservation” are improved by using OrthoIntegrate. Which means that the variance caused by different cell cycle states of the cells is conserved via OrthoIntegrate. Other parameters like the NMI-score are reduced. But this score for example is strongly influenced by the cell type labeling [3], which was focused only on main cell type groups in these datasets, regardless of the existence of subpopulation or mixed cell type population clusters. In other words, subclusters of different cell types were not annotated in detail. Due to the increased numbers of features that are included in OrthoIntegrate, the clustering might be more diverged, likely by species specific non-coding RNAs or other features, which are not included in the other databases. Therefore, the more divergent clustering, due the increased number of features in OrthoIntegrate combined with the broad cell type labeling might explain the slightly reduced NMI scores. “

- [ ] 3. The data analysis needs to be expanded further if there are findings with potential biological significance. For example, the authors mentioned, 'In cluster 25, we observe a group of genes showing increased expression in human FBs, and we also identify a set of genes that are negatively regulated in cluster 28 in human ECs.' However, there is no functional analysis, such as GO or KEGG pathway enrichment analysis, conducted to interpret the data and validate these findings.

We thank the reviewer for the valuable input. To expand the biological significance of the data analysis, we performed gene set enrichment analysis for genes that are either enriched in humans, mice or genes that are commonly regulated, in all other cell types. Furthermore, we plotted enriched genes for all other cell types. As all these analysis would exceed the scope of the supplementary figure, we uploaded them to the paper specific github account (https://github.com/MarianoRuzJurado/RuzJurado_et_al_2023/tree/main/Expanded_Analysis_Figures). Additionally, we performed GoTerm analysis on the genes that were regulated in the fibroblast cluster 25 and endothelial cell cluster 28. The results were incorporated in Figure 4B and 4C and the text was extended accordingly.

- [ ] 4. The discussion section is confusing. The authors should clarify whether the paper is primarily focused on research methods or data analysis. If it is a data analysis paper, the authors should conduct additional investigations to include further data analysis. If it is a research method paper, the authors should extend the discussion to relate to the algorithm itself.

According to the reviewers suggestions, we restructured the discussion section and added additional paragraphs regarding the data analysis and the benchmarking of OrthoIntegrate. Additionally we have strengthened the research part by incorporating the benchmarking results into the main part (Fig 2) of the paper.

Minor comments:

MinorThings:
- [ ] 1. The cell number for each sample and each clustered cell type is critical for assessing the reliability of the results; however, this information is not provided in the paper.

We thank the reviewer for this remark and added all QC statistics to the Supplementary Table 6.

- [ ] 2. As the mouse model is generated through chronic infarction, it raises the question of why very few T/B cell markers are found in immune cells in Figure 1F. Is it possible that these cell types are not adequately captured in the mouse samples? In data integration analysis, the audience may be more interested in understanding how species-specific cell types perform, particularly when, for instance, only macrophages are the dominant immune cells found in human samples.

We thank the reviewer for this comment. In single nuclei sequencing of isolated hearts, almost no T-cells or B-cells are found (Litviňuková et al. 2020). Also the heart cell Atlas by Litviňuková et.al.; Nature 2020 showed mainly Myeloid cells (see https://www.heartcellatlas.org/v2/global/). When we subsetted the immune cell cluster in our dataset, we also could not detect any t- or b-cell clusters (see Reviewer Fig.1 in the GitHub Repository [https://github.com/MarianoRuzJurado/RuzJurado_et_al_2023/blob/15e208c336b668402aa201cfc45d6433c1479ea6/Reviewer%20Figures/ReviewerFigure1.pdf]). However, for scientist interest in mouse or human specific immune cell responses through chronic inflammation, we provide detailed analysis of immune cells in our github repository (https://github.com/MarianoRuzJurado/RuzJurado_et_al_2023/tree/main/Expanded_Analysis_Figures/Immune%20cells). Also the other celltype are analyzed there in detail.

.- [ ] 3. On page 5, clarify "latter ones" in the sentence "Most of the latter ones were long non-coding RNAs with identical gene names."

We clarified the text in this paragraph and changed the sentence to:
"Most of the 86 matches found by lowercasing were long non coding RNAs with identical gene names"

Powered by Editorial Manager® and ProduXion Manager® from Aries Systems Corporation

	- [ ] 4. On page 5, correct the reference to Supplementary Figure 4A instead of Supplementary Figure 3A and Supplementary Table 3.<br><br>We changed the text accordingly and added Supp_Fig_4 to the main manuscript as Fig 2.<br><br><br>- [ ] 5. On page 16, replace "regulated genes" with "differentially expressed genes (DEGs)" to accurately represent what the authors referred.<br><br>We changed the text accordingly.<br><br>References<br>1. Brudno M, Malde S, Poliakov A, Do CB, Couronne O, Dubchak I, et al.. Glocal alignment: finding rearrangements during alignment. Bioinformatics. 19 Suppl 1:i54–622003;<br>2. Tanay A, Sebé-Pedrós A. Evolutionary cell type mapping with single-cell genomics. Trends Genet. 37:919–322021;<br>3. Song Y, Miao Z, Brazma A, Papatheodorou I. Benchmarking strategies for cross-species integration of single-cell RNA sequencing data. Nat Commun. 14:64952023;<br>4. Otero-Garcia M, Xue Y-Q, Shakouri T, Deng Y, Morabito S, Allison T, et al.. Single-soma transcriptomics of tangle-bearing neurons in Alzheimer's disease reveals the signatures of tau-associated synaptic dysfunction. bioRxiv.<br>5. Luecken MD, Büttner M, Chaichoompu K, Danese A, Interlandi M, Mueller MF, et al.. Benchmarking atlas-level data integration in single-cell genomics. Nat Methods. 19:41–502022;
**Additional Information:**	
**Question**	**Response**
Are you submitting this manuscript to a special series or article collection?	No
**Experimental design and statistics**<br><br>Full details of the experimental design and statistical methods used should be given in the Methods section, as detailed in our Minimum Standards Reporting Checklist. Information essential to interpreting the data presented should be made available in the figure legends.<br><br>Have you included all the information requested in your manuscript?	Yes
**Resources**<br><br>A description of all resources used, including antibodies, cell lines, animals and software tools, with enough information to allow them to be uniquely identified, should be included in the Methods section. Authors are strongly	Yes

encouraged to cite Research Resource Identifiers (RRIDs) for antibodies, model organisms and tools, where possible.<br><br>Have you included the information requested as detailed in our Minimum Standards Reporting Checklist?	
**Availability of data and materials**<br><br>All datasets and code on which the conclusions of the paper rely must be either included in your submission or deposited in publicly available repositories (where available and ethically appropriate), referencing such data using a unique identifier in the references and in the "Availability of Data and Materials" section of your manuscript.<br><br>Have you have met the above requirement as detailed in our Minimum Standards Reporting Checklist?	Yes

Manuscript Click here to access/download;Manuscript;Gigascience Revision Paper.docx

Improved integration of single cell transcriptome data demonstrates common and unique signatures of heart failure in mice and humans

Mariano Ruz Jurado[1,2,3], Lukas S. Tombor[1,2], Mani Arsalan[4], Tomas Holubec[4], Fabian Emrich[4], Thomas Walther[2,3,4], Wesley Abplanalp[1,2,3], Ariane Fischer[1], Andreas M. Zeiher [1,2,3], Marcel H. Schulz[1,2,3], Stefanie Dimmeler[1,2,3], David John[1,3]

1.) Institute of Cardiovascular Regeneration, Theodor-Stern-Kai 7, 60590 Frankfurt am Main

2.) German Centre for Cardiovascular Research (DZHK), Frankfurt am Main, Germany

3.) Cardio-Pulmonary Institute (CPI), funded by the German Research Foundation (DFG)

4.) Department of Cardiovascular Surgery, Goethe University Hospital, 60590 Frankfurt am Main

Abstract

Background

Cardiovascular research heavily relies on mouse (*mus musculus*) models to study disease mechanisms and to test novel biomarkers and medications. Yet, applying these results to patients remains a major challenge and often results in non-effective drugs. Therefore, it is an open challenge of translational science to develop models with high similarities and predictive value. This requires a comparison of disease models in mice with diseased tissue derived from humans.

Results

To compare the transcriptional signatures at single cell resolution, we implemented an integration pipeline called *OrthoIntegrate* which uniquely assigns orthologues and therewith

merges single cell data (scRNA-SEQ) of different species. The pipeline has been designed to be as easy to use and is fully integrable in the standard Seurat workflow.

We applied *OrthoIntegrate* on scRNA-SEQ from cardiac tissue of heart failure patients with reduced ejection fraction (HFrEF) and scRNA-SEQ from the mice after chronic infarction, which is a commonly used mouse model to mimic HFrEF. We discovered shared and distinct regulatory pathways between human HFrEF patients and the corresponding mouse model. Overall, 54% of genes were commonly regulated including major changes in cardiomyocyte energy metabolism. However, several regulatory pathways, e.g. angiogenesis, were specifically regulated in humans.

Conclusion

The demonstration of unique pathways occurring in humans indicate limitations on the comparability between mice models and human HFrEF and show that results from the mice model should be validated carefully. *OrthoIntegrate* is publicly accessible (https://github.com/MarianoRuzJurado/OrthoIntegrate) and can be used to integrate other large data sets to provide a general comparison of models with patients data.

Keywords

cross-species analysis, cardiovascular disease, heart failure with reduced ejection fraction, coronary artery ligation, single cell integration, cross species integration workflow

Introduction

Animal experiments are a powerful tool to improve our understanding of pathophysiological conditions and to predict responses to new therapeutic approaches [1]. However, due to ethical considerations they are controversially discussed [2], and their predictive capacity for toxicity and drug responses is limited [3,4]. Especially mice are commonly used to model

human diseases as they are relatively inexpensive, have short generation times and have large numbers of offspring. Additionally mice have a relatively close physiological and phylogenetic relationship with humans [5], [6]. Mice protein-coding genes are on average 85% identical to humans [4] and over 90% of both genomes have regional conserved synteny [7]. Due to these advantageous breeding characteristics and their high sequencing conservation to humans, hundreds of different mouse models have been developed to study human diseases [8] like heart failure [9] or even diseases that do not occur naturally in mice like Alzheimer's or Parkinson's disease [10].

To study cardiovascular diseases, which remain the leading cause of morbidity and mortality in the aging society, the ligation of the left anterior descending coronary artery model (LAD) is often used to induce myocardial infarction, which results in ischemic heart failure with reduced ejection fraction (HFrEF) [11,12]. Thereby, the LAD is ligated to mimic the clotted artery as it occurs after infarction. While short term reperfusion then allows to mimic the reopening of the coronary artery by catheter based interventions, often chronic ligation is used to induce heart failure over the course of > 4 weeks. As this method describes a similar decline in heart function, scientists use LAD mouse models to simulate HFrEF and develop and test new therapeutic strategies [13–15]. Patients who suffer from HFrEF, are unable to pump sufficient amounts of blood to meet the demands of body organs [16].

To address the comparability of HFrEF in human to mouse models, we used single nuclei RNA sequencing data, enabling us to assess transcriptional regulatory pathways in all cardiac cell populations with high resolution and accuracy [17,18]. In order to analyze scRNA-seq data from various samples, integration pipelines were developed to combine individual cells from different subjects into clusters with similar expression patterns [18,19]. Yet these bioinformatic tools can only integrate datasets from identical species. Several studies developed algorithms to compare mRNA expression patterns across species [20–22]. However, a standardized and easy way to compare single cell/nuclei RNA sequencing data sets of human and mouse by directly integrating the data is still missing [18,23,24]. To overcome these limitations and the

highly increasing demand for comparison of various organisms prompted us to develop a R package called *OrthoIntegrate*. It features a pipeline for integration of single cell datasets and orthologue assignment, allowing the simple integration of data from animal models and human patients. For the orthologue assignment process, we implemented an algorithm in the workflow that adjusts the different nomenclature between species before the integration takes place, by using the databases of Ensembl, NCBI, and Uniprot. [25–27]. Using our newly established pipeline, which is completely compatible with standard seurat workflows, we explored the gene expression patterns in mouse models of HFrEF compared to human samples. While 54% of genes were commonly regulated in both species, we also observed significant differences in differentially expressed genes and regulated pathways in patients with heart diseases than in the corresponding mouse model.

Results

One to one orthologue assignments

To integrate single cell data from different species, we established a table of gene names, which contains one human gene for each mouse gene, by which it will be replaced (one-to-one orthologues). We performed the same approach for generating a table of gene names between human and zebrafish genes.

In order to generate these one-to-one orthologues, we utilized the Needleman-Wunsch algorithm [28] to perform a pairwise global alignment between possible orthologues retrieved by Ensembl database. This calculation determines alignment scores based on differences in the amino acid or nucleotide sequences. In case no orthologues were found, nor a protein- or nucleotide sequence is available for a particular gene, a lowercase matching of the human gene is searched for in the mouse gene database (Suppl. Fig. 1A).

The Ensembl database assigned a total of 21,428 mouse orthologues to our human gene ID symbols. However, only 77% (16,573) of these were uniquely assigned. Through our *OrthoIntegrate* pipeline, we increased the number of assignments to 82% (17,504). Hereby,

714 mouse genes were assigned by protein sequence alignment, 89 through nucleotide sequence alignment, 42 by using the Levenshtein distance between gene names and 86 using our lowercase matching approach. Most of the 86 matches found by lowercasing were long non coding RNAs with identical gene names. We then proceeded by filtering the human and mice data by these orthologues in our pipeline and replaced the mice nomenclature by the human nomenclature for the corresponding samples (Suppl. Fig. 1B). In the end, we could assign ~82% of the mice genes to human orthologues (Suppl. Table 2). Replacing mouse gene names with the human orthologue allowed us to integrate the human patient data with the mouse model data into one single cell object (Fig. 1A). Moreover, we aimed to underscore the versatility of OrthoIntegrate. Therefore, we integrated and clustered scRNA-seq datasets related to Alzheimer's disease from human, mouse and zebrafish with *OrthoIntegrate* pipeline (Suppl. Fig. 6). We successfully created clusters representing excitatory and inhibitory neurons, as well as astrocytes, in the three species (Suppl. Fig. 6A-B). Given the focus and the size of the human study and data most of the excitatory neurons found were of human origin, but we showed that excitatory neurons found in mice were also assigned to the same clusters and showed comparable marker genes (Suppl. Fig. 6A-D). Similar results were obtained for inhibitory neurons and astrocytes proving a successful integration of all three datasets (Suppl. Fig. 6C-F).

Cell type composition in human and mouse upon HFrEF

After demonstrating the practicality of the integrated dataset, the biological differences of the human mouse datasets were analyzed. The absence of species specific clusters in the combined UMAP plot confirms that human and mouse hearts comprise similar cell types and gene expression patterns (Fig. 1B). This is additionally verified by similar cell type specific marker genes in both species in the different cell clusters (Fig. 1E-F). The specific marker genes allowed the annotation of the clusters into cardiomyocytes (CMs), pericytes (PCs), smooth muscle cells (SMCs), fibroblasts (FBs), endothelial cells (ECs), immune cells (ICs), as well as neuronal cells (NCs) (Fig. 1C). In addition, we analyzed how the distribution of cell

types was affected by the heart failure phenotype. Thereby a 20% decrease in human CM was observed when comparing the control samples with the HFrEF samples (45% -> 25%) (Fig. 1D). However, in mice, there is no difference in the numbers of CMs between the infarcted and control mice (both comprise about ~25% CM) (Fig. 1D). Furthermore, we found differences in the distribution of ECs in the human versus mouse samples. Specifically, we observed a significant increase in the EC population in samples from HFrEF patients (~30 %) compared to healthy hearts (~8%). In contrast, we noticed decreased EC numbers in mice upon infarction (from 25% in controls to 18% after chronic infarction). Minor changes are also observed in the contributions of other cell types (Fig. 1D).

Comparison to other integration methods

We carefully inspected our data to determine species specific distribution by creating UMAP plots of all cells in our integrated object. Figure 1B shows that cells of mouse and human origin commingled in all clusters, which indicates a successful integration based on the cell types and not on the species. We additionally compared our *OrthoIntegrate* pipeline to other orthologue databases and tools to assess the advantages of our orthologue assignments. For this purpose, we created the same scRNA-SEQ datasets using the different orthologue lists OMA, Biomart and InParanoid [29–31]. Visualization of the integration by UMAP plots shows an integration of human and mouse-derived cells in the individual cell clusters also with the alternative orthologous list (Suppl. Fig. 2A-C). However, besides the visual impression, quantitative metrics were used to assess the quality of the clustering, we calculated silhouette coefficients, which measure the quality of the clustering independent from the number of clusters. Integration by *OrthoIntegrate* resulted in the highest silhouette coefficients compared to the other orthologue databases, suggesting an improved clustering (Fig. 2A). Additionally, it is noteworthy to mention that our pipeline achieved by far the most 1:1 protein coding and lncRNA coding orthologous pairs in comparison to the other described methods (Fig. 2B-C). To further determine the clustering quality after integration, we computed supplementary

metrics recommended by the single-cell integration benchmark scib package [32] and the Orthology Benchmark Service. We also calculated the Species Mixing Score and Bio Conservation Score, following the guidelines of the BENGAL pipeline (Fig. 2D) [32,33]. Remarkably, our method not only achieved the highest number of uniquely mapped orthologous pairs but also demonstrated high performance across individual metrics in comparison with alternative tools (Fig. 2D-F).

Differential gene expression between mice and humans

The differentially expressed gene (DEG) analysis, showed strong similarities in the regulated genes upon HFrEF. However, some genes showed differences in their expression patterns. Mainly when the cell types were analyzed individually. Overall, we found a comparable number of DEGs in both species (4141 in humans, 4654 in mice).

The average of commonly regulated genes per cell type (Fig 3A; left side) showed that around 54% of DEGs found in humans were also regulated in mice, with minor differences between cell types. Upregulated genes showed a generally higher comparability compared to downregulated genes (Fig. 3B). Only in smooth muscle cells many more human specific DEGs were regulated in opposite directions (Fig. 3B, right upper panel). Averaging the mouse regulated DEGs (Fig 3A; right side) showed that only about 34% of the cell type specific DEGs in mice were regulated in humans, indicating a more substantial transcriptional effect of the LAD model compared to the human disease.

Figure 3B separately shows the up (top panel) and down (lower panel) regulated genes in humans and their regulation in mice. For the upregulated genes in humans, around 50-70% of the corresponding mouse genes were also upregulated, around 25% were not regulated and only about 5-20% percent were regulated in the opposite direction. Suggesting that overall activation occurs mainly in similar expression pathways across all cell types. In the downregulated genes in humans, we observed a strikingly low number of commonly regulated genes in cardiomyocytes. There, only 23.3% of the downregulated genes were also decreased

in mice. Most of them were either not regulated (48.2%) or even upregulated in mice (28.5%). The other cell types show a higher percentage of commonly downregulated genes.

We visualized all expression changes in one heatmap to further validate individual gene changes upon HFrEF (Fig. 4 A/B). Thereby, we found that around 30% of the genes show no changes in their expression upon heart failure (Fig. 4A, cluster 1). Most expression changes are consistently observable in all cell types (cluster 2-23) and therefore appear as general responses to injury which cannot be attributed to individual cell types. However, the remaining 16 clusters show cell type specific expression patterns (Fig. 4B). For example, cluster 25 holds a set of genes that show increased expression of genes in human FBs. Whereas cluster 28 in human ECs contains many genes that are down regulated. These changes are not detectable in other cell types for these genes and are therefore of utmost interest to follow up on specific gene expression changes in species specific cells. Similar patterns were found by observing commonly regulated genes (Fig. 4C). For humans, the largest number of DEGs were found in all cell types (1087 DEGs). The second largest groups contain DEGs that are found only in the individual cell types (Fig. 4C; Suppl. Fig. 4A). Thus, we identified 687 DEGs specific to human CM and 208 DEGs that can only be found in ECs. If we now determine the distribution of DEGs in mice, one finds larger populations of cell type specific genes and fewer, which are found in all populations (n = 228). Notably we found far fewer DEGs in the mouse SMCs than in the human samples. However, this could be related to the total number of SMCs in mice, which is far less in mice than in human samples (Fig. 1C & 3A). This could explain the lower number of DEGs found in all cell types. When we excluded SMCs from the common DEG population, we observed a similar number of DEGs in all cell types as in humans previously (Suppl. Fig. 4B).

Further, we analyzed the highest upregulated genes per cell type in humans and mice along with the regulation of that gene in the other species. Hereby, we observed how the genes with the largest changes in human heart failure patients behave in the respective mouse model (Suppl. Fig. 3).

We observed that the expression of the most regulated genes in human cell types show comparably less regulation in the mouse models. For example, we found *LDB2,* a gene of the LIM-Domain family, in human CMs as highly upregulated (Log2FC = 2.15) (Suppl. Fig. 3A). The LIM-Domain family genes are well known as adapter molecules that allow the assembly of transcriptional regulatory complexes in CM. However, in mice, *LDB2* is only mildly regulated upon HFrEF (Log2FC = 0.38). Other genes such as the VEGF receptor *FLT1,* which is upregulated in human cardiomyocytes, show a downregulation in mice CMs. This demonstrates that some genes have completely different expression patterns in humans and mice. However, some genes share similar regulation in their respective cell types. Thus, we observed that Phosphodiesterase 4D (*PDE4D)* and ADP Ribosylation Factor Like GTPase 15 (*ARL15)* show similar changes in ECs. Among the ten most upregulated genes in the mouse model data, we found three genes that also show a significant increase in their expression in humans (*RBPJ*, *SLC9A9*, *RUNX1*) (Suppl. Fig. 3B). The other genes, however, show little to no change. In contrast, if we investigate the expression changes in ECs, DEGs show an opposite direction in their expression change (*RBPJ*, *PID1*, *SLC9A9*). These differential gene expressions in the cell types suggest that some cell type specific responses may be different between human patients and mouse models.

Pathway enrichment results in cardiomyocytes

To address whether the relatively high number of significantly regulated genes indicate overall changes in pathways and pathological processes or whether the differences relate more to the alternative use of genes with similar functions in mice and humans, we further determined the implications for overall pathways in the individual cell types. Figure 4 shows a *simplifyEnrichment* heatmap cluster with word clouds of gene ontology terms regulated in human or mouse cardiomyocytes. We generally observe more significantly enriched GSEA terms in humans than in mice (Fig. 5A). Important pathway terms regarding mitochondrial energy production and the electron chain were enriched in both species. Other terms involving developmental processes are enriched in humans compared to mice. Additionally, we

investigated the set of genes found in cluster 25 and cluster 28 in more detail (Fig. 4B; Fig. 5B-C). Gene Ontology (GO) analysis on subsections of genes found in these clusters revealed a change in pathways associated with cell adhesion and extracellular processes (Fig. 5B). The second subsection of cluster 28 was associated with terms regarding cell differentiation processes, like “epithelial cell differentiation” or “angiogenesis” (Fig. 5C).

We identified cell type specifically regulated pathways upon HFrEF. Therefore, we investigated how the enriched signaling pathways differ between humans and mice in cardiomyocytes. We observed larger differences for pathways that were specifically regulated in humans. Among the most regulated pathways, specifically detected in the human, we found the terms "actin filament organization" and "angiogenesis" (Fig. 6A). Genes associated with these pathways were explicitly upregulated in patients (Fig. 6B). These gene sets are not found among the regulated pathways in mice (Suppl. Table 4). Examples of angiogenesis-related genes, which are specifically induced in human heart failure but not in mouse models, including receptors such as the *VEGF*-receptor FLT1, or transcription factors like the mesenchyme homeobox protein 2 (MEOX2) (Fig. 6B). In addition, many GTPase regulatory genes were found specifically increased in humans, including *MCF2L* and *RASGRF2*, which are known to regulate *RAC1*, and *SPATA13*, which enables guanyl-nucleotide exchange factor activity [34,35]. In contrast, we observed that signaling pathways mainly dealing with energy metabolism are commonly regulated in patients with heart disease as well as in mouse models. The genes included in pathways, such as “ATP biosynthetic process”, “mitochondrial ATP synthesis”, “aerobic electron transport chain” and “cellular respiration”, show significant downregulation compared to their corresponding control (Fig. 6C). These data suggest conservation of disturbed mitochondrial metabolism in both mice and humans upon heart failure.

On the other hand, pathways such as "Wnt signaling pathway", "actin-myosin filament sliding" and “regulation of cell morphogenesis“ are upregulated specifically in the mouse HFrEF model (Fig. 6A). Genes associated with Wnt signaling include *LRP6*, a known inhibitor of cardiomyocyte proliferation [36], and the serine/threonine-protein kinase *MARK2*, which

regulates the stability of microtubules through phosphorylation and inactivation of several microtubule-associated proteins [36].

Furthermore, we repeated the GSEA analysis with the identified ECs in the human and mouse model data to gain further insight into the different cell types (Suppl. Fig. 5). Here, we found human-specific regulated terms such as "cardiac contraction" and "regulation of axonogenesis" (Suppl. Fig. 5A) that we only find in ECs but not in the previously analyzed CMs. The genes in these sets show a distinct regulation only observed in human data (Suppl. Fig. 5B). When we examined the commonly regulated metabolic pathways. We found similar terms and changes in gene expression related to impaired mitochondrial metabolism in EC as we had previously observed in CMs (Suppl. Fig. 5C). In ECs, we also found similar mouse-specific terms such as "cell morphogenesis" and the "Wnt signaling pathway", but also newly discovered pathways such as "positive regulation of steroid hormone secretion". Steroid hormones have been shown to coordinate microvascular function in obese mice endothelium [37]. Based on these results, one might speculate that this regulatory function is mouse specific. GSEA analysis for all other cell types can be found under https://github.com/MarianoRuzJurado/RuzJurado_et_al_2023/tree/main/Expanded_Analysis_Figures/. All Source code for this study can be found in the paper specific Github repository (https://github.com/MarianoRuzJurado/RuzJurado_et_al_2023).

Discussion

The ever growing number of published single cell experiments enables scientists to deepen the knowledge about transcriptional changes of individual cell types and species specific regulatory changes, upon disease conditions. Particular combination of single cell datasets from different species in the same UMAP projection allows the detection of well conserved or species specific regulatory networks [38–40].

Therefore, integrating datasets from different species with a well curated list of orthologues, has significant advantages and simplifies comparisons among species.

Here we propose *OrthoIntegrate,* an R-package that enables scientists to integrate single cell datasets from different species into a shared dimensional space. To generate high quality and uniquely mapped orthologous lists between different species, we implemented a new pipeline that increases the ono-to-one assignment of ontologies to improve single cell integration. Compared to the Ensembl orthologous list (Biomart), our pipeline results in up to 10% more uniquely assigned orthologues between human and mouse. Compared to the other databases OMA and InParanoid, OrthoIntegrate contained 8.6% and 9.3% more one-to-one orthologues (Fig. 3F).

OrthoIntegrate additionally contains functions that use the extended orthologous assignments to streamline the integration of single cell datasets from humans and mice. Moreover, it is highly adaptable and can be easily customized to support other species.

We demonstrated the usability of combining cross-species single cell data by using data sets of human and mouse heart failure with reduced ejection fraction.

In order to evaluate the species mixing and the biological conservation of different integration methods, we applied certain metrics from the scib package [32,41], which were also suggested by Song et.al [32,33,41]. The results are summarized in Figure 2D. We found that most batch correction scores improve by using OrthoIntegrate.

For biological conservation scores, we demonstrate that some metrics, like the “cell cycle conservation” are improved by using OrthoIntegrate. Which means that the variance caused by different cell cycle states of the cells is conserved via OrthoIntegrate. Other parameters like the NMI-score are reduced. But this score for example is strongly influenced by the cell type labeling [33], which was focused only on main cell type groups in these datasets, regardless of the existence of subpopulation or mixed cell type population clusters. In other words, subclusters of different cell types were not annotated in detail. Due to the increased numbers of features that are included in OrthoIntegrate, the clustering might be more diverged, likely by species specific non-coding RNAs or other features, which are not included in the other databases. Therefore, the more divergent clustering, due the increased number of features in

OrthoIntegrate combined with the broad cell type labeling might explain the slightly reduced NMI scores.

Further examination of DEGs in the heart failure dataset , showed major differences in the cell type expression patterns, which are differentially regulated in humans or mice upon HFrEF. Yet commonly regulated pathways also reflect an evolutionary conserved transcriptomic answer to severe damage in heart cells. One example is the conserved downregulation of critical mitochondrial metabolic pathways, which provide ATP for the heart (Fig. 5, Fig. 6A/C). The heart is the most energy consuming organ, so maintaining mitochondrial function plays [42]. Here we could show that genes important for ATP biosynthesis and electron transport (e.g. *PGAM2*, *NDUFA1* and *TMEM126A*) are consistently downregulated in heart failure. *Pgam2* overexpression has been shown to reduce heart stress resistance in mice [43]. However, its role in humans has not been described so far. Also *NDUFA1*,has been shown to be downregulated by about 50% in LAD rats, by western blot and mass spectrometry [44].

Besides commonly regulated pathways, we found differences between humans and mice upon heart failure. In cardiomyocytes, many genes associated with "angiogenesis" were increased in humans but not in mice. For example, the *VEGF* receptor *FLT1* was significantly augmented in the human samples. *FLT1* primarily mediates *VEGF* signaling in endothelial cells, but its role in cardiomyocytes is less clear [45], besides high expression in human cardiomyocytes [46]. Functionally, *FLT1* was shown to partially mediate *VEGF* induced cardiomyocyte differentiation [47] and mediate cardiomyocyte calcium signaling and contractility in the embryonic zebrafish heart [48]. Cardiomyocyte specific deletion of *FLT1* was shown to worsen cardiac remodeling and hypertrophy induced by pressure overload [49], suggesting that the upregulation of its expression in humans may represent a compensatory cardioprotective mechanism that might not be conserved in mice.

A second human CM specific gene is *MEOX2*, which was assigned to "angiogenesis" because of its role in endothelial fatty acid transport [50] critical role in development of all muscle

lineages [51]. In cardiomyocytes, *MEOX2* overexpression blocks proliferation during heart morphogenesis causing proliferating cardiomyocytes to withdraw from the cell cycle [52]. These human CM specific genes have not been studied in mouse cardiomyocytes and their human specific regulation upon heart failure might be of utmost interest for future studies.

Among the pathways specifically enriched in mice we found predominant expression of genes associated with Wnt signaling. Although most identified genes have not been directly linked to cardiomyocyte-specific functions, Wnt signaling critically regulates cardiac hypertrophy, remodeling and regeneration [36,53]. Therefore, these findings and the other identified species specific pathways deserve more in depth validation and investigation.

To further demonstrate the functionality of OrthoIntegrate, we integrated scRNA-SEQ data from human [41], mouse [54] and zebrafish [54,55] brain tissue under Alzheimer's condition. Besides the evolutionary distance between these species, we could jointly cluster different cell types via OrthoIntegrate (Suppl. Fig 6 A-C) and detect commonly expressed marker genes within these cell clusters (Suppl. Fig 6 D-F).

In summary, our publicly available bioinformatic tool *OrthoIntegrate* simplifies the comparison of scRNA-SEQ datasets from humans and mice. Thereby we could identify conserved regulatory pathways upon heart failure. Furthermore, we identified cell type specific differences in both species. Also, we showed pathways like angiogenesis regulated explicitly in humans, and Wnt signaling pathways, specifically regulated in mice.

We anticipate that this study shows the benefits of the joint analysis of scRNA-SEQ data through *OrthoIntegrate.* Due to the growing number of scRNA-SEQ datasets, we hope that *OrthoIntegrate* encourages other scientists to perform comparative analysis between different species and thereby increasing knowledge about conserved or species specific pathway responses in various diseases. This could improve the effective development of novel treatment strategies for heart failure or other diseases.

Limitations

The main limitation of our orthologue assignment and sample integration pipeline is the dependence on reliable databases for orthologous lists. Another problem with this approach is that it fails to consider the biological functions of the possible orthologues but selects the orthologue with highest sequence similarity. Second, our biological example has some limitations. While a decent number of healthy controls is available, the number of patients with HFrEF is limited. Knowing the biological heterogeneity of heart failure and comorbidities, variations are expected and the samples may not represent the representative and most common spectrum of heart failure. Finally, although the mouse model used is commonly applied in cardiovascular research, there are significant limitation due to the lack of underlying coronary artery disease and therapeutic pharmacological and interventions as it is done in humans. The integration of increasingly available published data both from alternative mice models and data derived from human samples will allow a refined comparative analysis in the future.

Methods

Single cell pre-processing

Single-cell RNA-seq results were processed by CellRanger (10x Genomics) version 6.1.1 software. The first step consisted of demultiplexing and processing raw base count files by the implemented *mkfastq* tool. The human raw reads were mapped to the reference genome hg38 (GRCh38-2020) using Cellranger count, whereas the mouse raw reads were mapped to the reference genome mm10 (GRCm38-2020). The secondary data analysis was conducted

using the Seurat 4.1.0 package in R. The data sets were first combined into a Seurat object and then subjected to a filtering process. Barcodes with too low (< 300) or too high number of genes (> 6000) were sorted out and not considered further in the data analysis. In addition, barcodes with too low (< 500) and too high read counts (> 15000) were also sorted out. To further ensure no apoptotic cells or doublets were analyzed, we discarded barcodes with a high percentage of mitochondrial content (> 5%). The filtered gene counts were then logarithmized and normalized according to the tutorial for data analysis with Seurat. Baseline characteristics for the samples can be found in Supplement Table 1.

Study samples

The human heart samples used as controls were provided from the PRJEB39602 (Human Cell Atlas) project published in 2020. The heart tissue was obtained from deceased transplant organ donors who were between 45 and 70 years old and showed unremarkable cardiovascular history. Heart samples from patients with heart failure with reduced ejection fraction (HFrEF) were provided from the Frankfurt University Hospital and subsequently processed at the Institute of Cardiovascular Regeneration (Frankfurt am Main, Germany), where the processed mice samples (CTRL: n1-n3, HFrEF: n1-n4) were also gathered and sourced. The human heart failure samples as well as the mice control and heart failure samples are published in Array Express with the accession E-MTAB-13264. The remaining healthy mice samples (CTRL: n4-n9) were gathered by Vidal et al. (2019) and can be found using the Array Express Data Portal under E-MTAB-7869 (Supplement Table 1).

In order to provide another species and disease condition, we applied OrthoIntegrate on human, mice and zebrafish in Alzheimer disease (AD) condition. Therefore we gathered scRNA-SEQ data from the prefrontal cortex (location matched) of human and mouse and zebrafish via scRead (human and mouse data, disease n=2; healthy n=2) [56] and GEO

(GSE118577; n=3). The human and mouse samples originate from GSE129308 [41] and GSE143758 (AD) & GSE143758 (Healthy), respectively.

Orthologue assignment and sample integration

In order to ensure the integration of single cell datasets from different species, we coded a function to assign animal model orthologues to the human nomenclature (or vice versa) using gene transfer format (GTF) files provided by Ensembl (GRCh38, GRCm38). In order to detect only well annotated genes between the species, predicted genes were removed. Afterwards orthologues to the human genes were determined using the R package biomaRt. This assigned the majority of genes in our human GTF file to at least one orthologue. If there were several entries of possible orthologues in the Ensembl database, a protein sequence comparison was initiated. Therefore, protein sequences were retrieved from the Uniprot database for the human gene and all possible orthologues in the second species. These sequences were then aligned using the R package Biostrings 2.60.2. The alignment score was calculated based on the Needleman-Wunsch global alignment algorithm [28] with substitution matrices. For nucleotide sequences, the *nucleotideSubstitutionMatrix* function was used to produce a substitution matrix for all IUPAC nucleic acid codes based upon match and mismatch parameters. BLOSUM50 matrix was retrieved from the NCBI Matrix Compendium for the protein sequence. The gene IDs with the highest amino acid sequence similarity between their canonical sequences were assigned. If there are no entries for canonical sequences in Uniprot, the nucleotide sequence similarity comparison is initiated. For this step, the unpredicted mRNA sequences for the gene in the first species and for the possible orthologues in the second species were obtained from the NCBI database and aligned analogously to the previous step. If no unpredicted mRNA is available for an entry, the function retrieves the unpredicted non coding RNA of the gene. This ensures that non coding genes without mRNAs can still be assigned correctly. In case both RNA sequences are not retrievable, predicted versions of mRNA and non coding RNA are retrieved. If all these

assignment steps are not successful, the Levenshtein distance was used to compare the ID symbols for possible orthologues and the orthologue with the lowest Levenshtein distance was selected.

Many long-non-coding RNAs are not listed in orthologue databases, therefore a final lowercase matching step was performed to assign genes like *Malat1* to the human *MALAT1*. With this globally applicable list of orthologues between species, the datasets were now filtered by these and then merged into one object using Seurat's canonical correlation analysis (CCA) integration.

Clustering, metrics calculation and annotation

To classify cells into clusters based on their expressed genes, we used the *FindNeighbors* and *FindClusters* (resolution parameter = 0.3) function implemented in Seurat. These clusters are determined by applying the shared nearest neighbors (SNN) clustering algorithm and the Uniform Manifold Approximation and Projection (UMAP) dimension reduction.

Calculations of the silhouette coefficient are based on computing a distance matrix based on the cell embeddings matrix for principal component analysis (PCA) performed by Seurat. This distance matrix includes the information of cell-cell distance, which is necessary for calculating the silhouette coefficient with our calculated clusters in the function *silhouette* of the cluster package (version 2.1.4). Additionally, the coefficients of the samples were averaged for each object. For applying the python scib package we converted our Seurat objects into Anndata objects using the zellkonverter package (version 1.10.1). We computed graph connectivity, principal component regression comparison, silhouette batch, kBET, LISI and cell cycle conservation scores for defining the species mixing score. Furthermore, the bio conservation score was calculated by computing the species type LISI, isolated labels F1 score, as well as the previously mentioned silhouette coefficient. The total score was then calculated by a weighted addition of species mixing score and bio conservation score (0.5 * species mixing score + 0.5 * bio conservation score). We provided the UniprotIDs of the orthologous lists

obtained with the tools to be compared to the Orthology Benchmark web service to calculate the Schlicker similarity scores for enzyme classification conservation and gene ontology conservation.

The orthologous lists for OMA, Biomart and InParanoid were created by following their introductions on their tool descriptions and by using the same GTF files as before (GRCh38, GRCm38).

For the assignment of cell clusters to cell types, we used a reference object that we had previously manually annotated with marker genes from Tombor et al. 2021 [57]. Here, the R package SingleR can be used to adopt marker genes that were used for the previous annotation of clusters of the reference object. These are then transferred and compared to marker genes of the cell clusters of our object to be annotated. Thus, a reproducible annotation can be guaranteed with the help of an exactly annotated data set.

Differential gene expression analysis and gene ontology analysis

Detection of differentially expressed genes (DEGs) for the cell type specific clusters was performed by the hurdle model of the MAST package (version 1.20.0). Results were filtered by their Bonferroni-adjusted p-value ($p.adj < 0.05$). The totality of DEGs were represented by Sankey plots created with the R package networkD3 (version 0.4). Additionally, bar plots were created using R package ggplot2, representing human DEGs and their regulation in mice. DEGs were also divided according to their species and cell type assignment and then visualized for DEGs with a positive Log2FC and separately in another plot, for DEGs with a negative Log2FC. Here, DEGs occurring in both human and mouse for the respective cell type have been pooled. Visualization was done in the form of a Circos plot (R package circlize 0.4.14). The gene regulation heatmap was created using the log2FC of all identified genes and a k-means clustering (k = 40) (R package ComplexHeatmap 2.16.0). Visualization of distinct and similar populations of genes in the analyzed cell types per species was achieved by creating venn diagrams with the Jvenn webtool.

Gene Set Enrichment Analysis (GSEA) was performed using the R package clusterProfiler (version 4.2.2) and the GO Database. GSEA terms were calculated separately for each cell type. The terms were sorted according to the Benjamini-Hochberg adjusted p.value and evaluated according to their “normalized enrichment distribution”, which gives information about the regulation of the genes in the described pathway. A heatmap was created by clustering the GSEA terms by their similar geneIDs. (R package simplifyEnrichment 1.10.0). Additionally, the GSEA results were plotted in dot plots. Specifically, for genes described in the pathway, the standard error of the mean (SEM) bar plot was created (for their averaged UMIs) by using the R package ggplot2. Gene Ontology (GO) analyses were performed using the subsection of genes found in cluster 25 and cluster 28 as input for the webtool Metascape.

Acknowledgment

The study was supported by grants from the German Centre for Cardiovascular Research (DZHK) to D.J. and S.D., the German Research Foundation (DFG; Exc2026/1) and the Dr. Rolf M. Schwiete Stiftung, Projekt 08/2018 to S.D.

Code availability

The *OrthoIntegrate* package containing the integration pipeline and the orthologue algorithm are available on Github (github.com/MarianoRuzJurado/OrthoIntegrate). Additionally, codes for R and Python analysis and plots of data presented in this study are available on another GitHub repository (github.com/MarianoRuzJurado/RuzJurado_et_al_2023).

Data availability

The single nuclei data for humans have been deposited in the Human Cell Atlas (HLC) database and can be accessed through the HCA Data Portal. The mice sequencing data are available through ArrayExpress under the accession number E-MTAB-7869.

Ethics declaration

The authors declare no competing interests.

References

1. Ericsson AC, Crim MJ, Franklin CL. A brief history of animal modeling. *Mo Med*. 110:201–52013;

2. Wall RJ, Shani M. Are animal models as good as we think? *Theriogenology*. 69:2–92008;

3. Shanks N, Greek R, Greek J. Are animal models predictive for humans? *Philos Ethics Humanit Med*. 4:22009;

4. Uhl EW, Warner NJ. Mouse Models as Predictors of Human Responses: Evolutionary Medicine. *Curr Pathobiol Rep*. 3:219–232015;

5. . The Mouse in Biomedical Research: History, Wild Mice, and Genetics. Elsevier;

6. Riehle C, Bauersachs J. Small animal models of heart failure. *Cardiovasc Res*. 115:1838–492019;

7. Mouse Genome Sequencing Consortium, Waterston RH, Lindblad-Toh K, Birney E, Rogers J, Abril JF, et al.. Initial sequencing and comparative analysis of the mouse genome. *Nature*. 420:520–622002;

8. Bult CJ, Blake JA, Smith CL, Kadin JA, Richardson JE, Mouse Genome Database Group. Mouse Genome Database (MGD) 2019. *Nucleic Acids Res*. 47:D801–62019;

9. Noll NA, Lal H, Merryman WD. Mouse Models of Heart Failure with Preserved or Reduced Ejection Fraction. *Am J Pathol*. 190:1596–6082020;

10. Breschi A, Gingeras TR, Guigó R. Comparative transcriptomics in human and mouse. *Nat Rev Genet*. 18:425–402017;

11. Dayeh NR, Tardif J-C, Shi Y, Tanguay M, Ledoux J, Dupuis J. Echocardiographic validation of pulmonary hypertension due to heart failure with reduced ejection fraction in mice. *Sci Rep*. 8:13632018;

12. Sawall S, Franke D, Kirchherr A, Beckendorf J, Kuntz J, Maier J, et al.. In Vivo Quantification of Myocardial Infarction in Mice Using Micro-CT and a Novel Blood Pool

Agent. *Contrast Media Mol Imaging*. 2017:26170472017;

13. Van Craeyveld E, Jacobs F, Gordts SC, De Geest B. Low-density lipoprotein receptor gene transfer in hypercholesterolemic mice improves cardiac function after myocardial infarction. *Gene Ther*. 19:860–712012;

14. Swynghedauw B. Molecular mechanisms of myocardial remodeling. *Physiol Rev*. 79:215–621999;

15. Ertl G, Frantz S. Healing after myocardial infarction. *Cardiovasc Res*. 66:22–322005;

16. Vigen R, Maddox TM, Allen LA. Aging of the United States population: impact on heart failure. *Curr Heart Fail Rep*. 9:369–742012;

17. Jovic D, Liang X, Zeng H, Lin L, Xu F, Luo Y. Single-cell RNA sequencing technologies and applications: A brief overview. *Clin Transl Med*. 12:e6942022;

18. Stuart T, Butler A, Hoffman P, Hafemeister C, Papalexi E, Mauck WM 3rd, et al.. Comprehensive Integration of Single-Cell Data. *Cell*. 177:1888–902.e212019;

19. Hwang B, Lee JH, Bang D. Single-cell RNA sequencing technologies and bioinformatics pipelines. *Exp Mol Med*. 50:1–142018;

20. Lu Y, Rosenfeld R, Nau GJ, Bar-Joseph Z. Cross species expression analysis of innate immune response. *J Comput Biol*. 17:253–682010;

21. Kristiansson E, Österlund T, Gunnarsson L, Arne G, Larsson DGJ, Nerman O. A novel method for cross-species gene expression analysis. *BMC Bioinformatics*. 14:702013;

22. Seok J, Warren HS, Cuenca AG, Mindrinos MN, Baker HV, Xu W, et al.. Genomic responses in mouse models poorly mimic human inflammatory diseases. *Proc Natl Acad Sci U S A*. 110:3507–122013;

23. Parekh S, Ziegenhain C, Vieth B, Enard W, Hellmann I. zUMIs - A fast and flexible pipeline to process RNA sequencing data with UMIs. *Gigascience*. 2018; doi: 10.1093/gigascience/giy059.

24. Korsunsky I, Millard N, Fan J, Slowikowski K, Zhang F, Wei K, et al.. Fast, sensitive and accurate integration of single-cell data with Harmony. *Nat Methods*. Nature Publishing Group; 16:1289–962019;

25. Cunningham F, Allen JE, Allen J, Alvarez-Jarreta J, Amode MR, Armean IM, et al.. Ensembl 2022. *Nucleic Acids Res*. 50:D988–952022;

26. Sayers EW, Bolton EE, Brister JR, Canese K, Chan J, Comeau DC, et al.. Database resources of the national center for biotechnology information. *Nucleic Acids Res*. 50:D20–62022;

27. UniProt Consortium. UniProt: the universal protein knowledgebase in 2021. *Nucleic Acids Res*. 49:D480–92021;

28. Needleman SB, Wunsch CD. A general method applicable to the search for similarities in the amino acid sequence of two proteins. *J Mol Biol*. 48:443–531970;

29. Altenhoff AM, Train C-M, Gilbert KJ, Mediratta I, Mendes de Farias T, Moi D, et al.. OMA orthology in 2021: website overhaul, conserved isoforms, ancestral gene order and more. *Nucleic Acids Res*. 49:D373–92021;

30. Smedley D, Haider S, Ballester B, Holland R, London D, Thorisson G, et al.. BioMart--biological queries made easy. *BMC Genomics*. 10:222009;

31. O’Brien KP, Remm M, Sonnhammer ELL. Inparanoid: a comprehensive database of eukaryotic orthologs. *Nucleic Acids Res*. 33:D476–802005;

32. Luecken MD, Büttner M, Chaichoompu K, Danese A, Interlandi M, Mueller MF, et al.. Benchmarking atlas-level data integration in single-cell genomics. *Nat Methods*. 19:41–502022;

33. Song Y, Miao Z, Brazma A, Papatheodorou I. Benchmarking strategies for cross-species integration of single-cell RNA sequencing data. *Nat Commun*. 14:64952023;

34. Kawasaki Y, Sagara M, Shibata Y, Shirouzu M, Yokoyama S, Akiyama T. Identification and characterization of Asef2, a guanine-nucleotide exchange factor specific for Rac1 and Cdc42. *Oncogene*. 26:7620–72672007;

35. Huang S-C, Chen Y-M, Hu Y-Y, Shi Y-J, Xiao Q-W, Li Z, et al.. Downregulation of MCF2L Promoted the Ferroptosis of Hepatocellular Carcinoma Cells through PI3K/mTOR Pathway in a RhoA/Rac1 Dependent Manner. *Dis Markers*. 2022:61389412022;

36. Wu Y, Zhou L, Liu H, Duan R, Zhou H, Zhang F, et al.. LRP6 downregulation promotes cardiomyocyte proliferation and heart regeneration. *Cell Res*. 31:450–622021;

37. Biwer LA, Carvajal BV, Lu Q, Man JJ, Jaffe IZ. Mineralocorticoid and Estrogen Receptors in Endothelial Cells Coordinately Regulate Microvascular Function in Obese Female Mice. *Hypertension*. 77:2117–262021;

38. Balachandran S, Pozojevic J, Sreenivasan VKA, Spielmann M. Comparative single-cell analysis of the adult heart and coronary vasculature. *Mamm Genome*. 2022; doi: 10.1007/s00335-022-09968-7.

39. Butler A, Hoffman P, Smibert P, Papalexi E, Satija R. Integrating single-cell transcriptomic data across different conditions, technologies, and species. *Nat Biotechnol*. 36:411–202018;

40. Baron M, Veres A, Wolock SL, Faust AL, Gaujoux R, Vetere A, et al.. A Single-Cell Transcriptomic Map of the Human and Mouse Pancreas Reveals Inter- and Intra-cell Population Structure. *Cell Syst*. 3:346–60.e42016;

41. Otero-Garcia M, Xue Y-Q, Shakouri T, Deng Y, Morabito S, Allison T, et al.. Single-soma transcriptomics of tangle-bearing neurons in Alzheimer’s disease reveals the signatures of tau-associated synaptic dysfunction. bioRxiv.

42. Huss JM, Kelly DP. Mitochondrial energy metabolism in heart failure: a question of balance. *J Clin Invest*. 115:547–552005;

43. Okuda J, Niizuma S, Shioi T, Kato T, Inuzuka Y, Kawashima T, et al.. Persistent overexpression of phosphoglycerate mutase, a glycolytic enzyme, modifies energy metabolism and reduces stress resistance of heart in mice. *PLoS One*. 8:e721732013;

44. Liu T, Chen L, Kim E, Tran D, Phinney BS, Knowlton AA. Mitochondrial proteome remodeling in ischemic heart failure. *Life Sci*. 101:27–362014;

45. Kurotsu S, Osakabe R, Isomi M, Tamura F, Sadahiro T, Muraoka N, et al.. Distinct expression patterns of Flk1 and Flt1 in the coronary vascular system during development and after myocardial infarction. *Biochem Biophys Res Commun*. 495:884–912018;

46. Karlsson M, Zhang C, Méar L, Zhong W, Digre A, Katona B, et al.. A single-cell type transcriptomics map of human tissues. *Sci Adv*. 2021; doi: 10.1126/sciadv.abh2169.

47. Chen Y, Amende I, Hampton TG, Yang Y, Ke Q, Min J-Y, et al.. Vascular endothelial growth factor promotes cardiomyocyte differentiation of embryonic stem cells. *Am J Physiol Heart Circ Physiol*. 291:H1653–82006;

48. Rottbauer W, Just S, Wessels G, Trano N, Most P, Katus HA, et al.. VEGF-PLCgamma1 pathway controls cardiac contractility in the embryonic heart. *Genes Dev*. 19:1624–342005;

49. Mei L, Huang Y, Lin J, Chu M, Hu C, Zhou N, et al.. Increased cardiac remodeling in cardiac-specific Flt-1 receptor knockout mice with pressure overload. *Cell Tissue Res*. 362:389–982015;

50. Coppiello G, Collantes M, Sirerol-Piquer MS, Vandenwijngaert S, Schoors S, Swinnen M, et al.. Meox2/Tcf15 heterodimers program the heart capillary endothelium for cardiac fatty acid uptake. *Circulation*. 131:815–262015;

51. Skopicki HA, Lyons GE, Schatteman G, Smith RC, Andrés V, Schirm S, et al.. Embryonic expression of the Gax homeodomain protein in cardiac, smooth, and skeletal muscle. *Circ Res*. 80:452–621997;

52. Fisher SA, Siwik E, Branellec D, Walsh K, Watanabe M. Forced expression of the homeodomain protein Gax inhibits cardiomyocyte proliferation and perturbs heart morphogenesis. *Development*. 124:4405–131997;

53. Bergmann MW. WNT signaling in adult cardiac hypertrophy and remodeling: lessons learned from cardiac development. *Circ Res*. 107:1198–2082010;

54. Habib N, McCabe C, Medina S, Varshavsky M, Kitsberg D, Dvir-Szternfeld R, et al.. Disease-associated astrocytes in Alzheimer’s disease and aging. *Nat Neurosci*. 23:701–62020;

55. Cosacak MI, Bhattarai P, Reinhardt S, Petzold A, Dahl A, Zhang Y, et al.. Single-Cell Transcriptomics Analyses of Neural Stem Cell Heterogeneity and Contextual Plasticity in a Zebrafish Brain Model of Amyloid Toxicity. *Cell Rep*. 27:1307–18.e32019;

56. Jiang J, Wang C, Qi R, Fu H, Ma Q. scREAD: A Single-Cell RNA-Seq Database for Alzheimer’s Disease. *iScience*. 23:1017692020;

57. Tombor LS, John D, Glaser SF, Luxán G, Forte E, Furtado M, et al.. Single cell sequencing reveals endothelial plasticity with transient mesenchymal activation after myocardial infarction. *Nat Commun*. 12:6812021;

Figure Legends

Fig.1: Integrated human and mouse snRNA-SEQ data of healthy and heart failure samples.

(A) Use case diagram of *OrthoIntegrate*: Shown are the steps that are run by the user within their standard Seurat workflow. First the Import function is used to create Seurat objects from scRNA-seq data, second orthologues are searched by the BuildOrtholog function and the third step creates an integrated object with uniform nomenclature by using the IntegrateObjects. (B) UMAP showing human cells (red) and mice cells (blue) in a common UMAP projection. In addition, cell types for the cell clusters can be seen. (C) UMAP with defined clusters according to Seurat's clustering, divided by species. Cells of mouse and human origin commingled in all clusters. There are no clusters formed that originated from only one of the two species. The cells were identified as cardiomyocytes (red), fibroblasts (yellow), endothelial cells (green), pericytes (turquoise), immune cells (blue), smooth muscle cells (purple) and neuronal cells (pink). (D) Bar plot showing cell composition of cell types in human (red) and mice (blue) samples. Samples were grouped based on their origin into human controls from the left ventricle (Human-CTRLlv), human HFrEF (Human-HFrEF), mouse controls (Mice-CTRL), and mouse HFrEF model (Mice-HFrEF). Cell types were then analyzed for their composition from the previously mentioned groups and plotted. P-values above the certain groups were calculated by two-sided Student's t-test. (E) Dot plot depicting the average expression levels and expression proportions in human samples of the top ten feature genes for the found cell types. The size of the dot represents the proportion of cells expressing the indicated gene within a cell type, and the color indicates the average expression level of cells. (F) Dot plot depicting the average expression levels and expression proportions in mice samples of the top ten feature genes for the found cell types. Similar to (E) the size of the dot represents the proportion of cells expressing the indicated gene within a cell type, and the color indicates the average expression level of cells.

Fig. 2: Comparison of snRA-Seq data integration with orthologues from *OrthoIntegrate* and other orthologue databases. (A) Box plot showing the average silhouette coefficient for clusterings based on different databases and tools. The dark blue box stands for the silhouette coefficient of the clustering made with an orthologous list using the tool OMA (Orthologous matrix). It is followed by the results for biomaRt (light blue), InParanoid (green) and the pipeline *OrthoIntegrate* (yellow). On the y-axis you can see the value of the silhouette coefficient. Additionally, each silhouette coefficient was calculated for each sample and depicted as a circle in their species specific color. (B) Bar plot with number of orthologues found which codes for a protein (C) and bar plot with number of orthologues found which codes for lncRNA. On the x-axis the used tool is depicted. (D) Table showing results of different metric calculations to comprehend batch correction and biological conservation of clusterings based on orthologous lists of OMA, biomaRt, InParanoid and *OrthoIntegrate* (GC = graph connectivity, PCR = principal component regression comparison, bASW = batch average silhouette width, CCC = cell cycle conservation, NMI = normalized mutual information, ILF1 = isolated labels F1 score, SC = silhouette coefficient, SMS = species mixing score, BCS = bio conservation score). The color code represents low and high values and is scaled per column (low = green, brown; high = blue, yellow). (E) Schlicker similarity scores calculated for OMA (red), Biomart (green), InParanoid (blue) and OrthoIntegrate (purple) in terms of enzyme classification conservation (left) and gene ontology conservation (right). (F) Venn Diagram highlighting the numbers of uniquely found orthologues between human and mouse per tool and their overlap between each other (blue = OMA, red = biomart, green = biomart, yellow = *OrthoIntegrate*).

Fig.3: Similarities and differences revealed by DEG analysis

(A) Sankey plot illustrating the distribution of differentially regulated genes (DEG) in the corresponding cell types. The width of the paths illustrates the number of DEGs that are either human specific (yellow), detected in both species (light green) or mouse-specific (dark green). DEG analysis was performed for each cell type individually. Neuronal cells were omitted from all further analyses due to their insufficient number of cells in the mouse data. (B) Bar graph

of up (top) and down (bottom) regulated genes in humans, along with the expression in mice. The panels show genes that are either commonly regulated (left), regulated in humans and not regulated in mice (middle) and regulated in opposite directions.

Fig.4: DEG analysis shows similar and different populations of regulation in gene expression patterns upon heart failure in humans and mice. (A) Heatmap of log2FC values (Control vs HFrEF) for all genes and all cell types. The y-axis describes all genes (16.545) clustered by a k-means algorithm (k = 40). The x-axis shows the species and the additional clustering into the different cell types. Positive log2FCs are represented by a red color, while negative scores give a blue color. (B) Close-up of the 24-40 k-means clusters of log2FCs of genes in which most cell type-specific differences are observed. (C) Venn Diagrams of all identified DEGs in human (top) and mouse (bottom) (log2FC > 0.1 and p-adjusted < 0.05).

Fig.5: GSEA analysis reveals more regulated pathways in heart failure in human cardiomyocytes than in mice, with the terms found sharing many keywords. (A) Heatmap clustering significant GSEA results (p.adj < 0.25) of DEGs found in human and mouse cardiomyocytes by similar GeneIDs in the pathways. Bar graphs are shown on the left y-axis representing the number of pathways found in the respective cluster for the given species and condition. In addition, the adjusted p-value is color-coded from 1 (green) to the smallest p-value found ~0.025 (red). On the right side of the y-axis keywords describing the found pathways in that cluster are shown, where the size of the word represents its frequency in the terms (larger = most, smaller = less). (B) Bar graph showing the first 20 GO terms found by analyzing genes in cluster 25. Terms were sorted by their logarithmized and Bonferroni-adjusted p-values resulting in high significant pathways depicted first (p.adj < 0.05). (C) Bar graph similar to (B) with terms found in a subsection of genes in cluster 28.

Fig.6: Common and distinct regulated pathways found in human and mouse cardiomyocytes.

(A) Dot plot visualizing the ten most significant pathways for terms only to be found regulated in humans, commonly regulated and specific in mice. The size of the dots corresponds to the negative log10 of the Benjamini Hochberg adjusted p-value and the color-code represents the normalized enrichment score (NES), with upregulated pathways shown in red and downregulated pathways in blue. The y-axis depicts the description of the identified term.

(B) Bar plot with mean values for the amount of unique molecular identifiers (UMIs) in the cells for the shown genes. The genes are identified to be dissimilarly regulated between humans and mice for pathways specifically found in humans. (C) Bar graph similar to (B) with mean values for UMIs in cells for genes downregulated in both species for commonly found terms. (D) Bar graph similar to (B) and (C) with mean values for UMIs in cells for genes which are uniquely found to be regulated in terms specifically identified in mice. P-values above the certain groups were calculated by two-sided Student's t-test.

Supplement Fig.1: Integration process of human/mouse snRNA-SEQ data.

(A) UML-Activity-Flowchart showing orthologue assignment pipeline for human to mouse gene symbols. First, the Gene transfer format file (GTF) for humans (GRCh38) is used to get all annotated gene nomenclatures. Then all genes are filtered out which are only predicted and not clearly detected. This list is now searched for orthologues using the Ensembl database; all 1:1 assignments can be included in our orthologous list. In the case of multiple assignments, all possible replacements are examined according to their protein sequence and an alignment score is calculated according to the global sequence alignment. If there is no protein sequence in the Uniprot database, the alignment score is calculated based on the nucleotide sequence using the NCBI database. Now the gene with the best result is set as an orthologue. All unassigned genes are additionally compared with the GTF file of GRCm38 using a lowercase matching and if there is a match, they will be added to the orthologue list. If all these approaches for a gene do not result in an orthologue, a Levenshtein distance score

is calculated based on their gene names. (B) Single cell integration pipeline showing steps performed to integrate human and mouse scRNA-SEQ data in a joined UMAP projection. The scRNA-SEQ data from our human and mouse samples are first converted into Seurat objects and normalized. After that, clustering takes place and cell types can be determined. Using the orthologous list from our orthologue assignment algorithm, the objects can be subsetted according to the genes found and their nomenclature unified. This is followed by an integration into a single object and a clustering step.

Supplement Fig. 2: Overlapping of human and mouse cells after Seurat integration with tool specific orthologous list. (A) UMAPs showing human cells (red) and mice cells (blue) in a common UMAP projection for each tool used for integrating the data. First UMAP was performed on an object made with an orthologous list of OMA, followed by Biomart and InParanoid. The last UMAP shows the projection for the *OrthoIntegrate* pipeline. (B) Similar to (A), UMAPs are shown to visualize the clustering created with the R package Seurat using the Louvain algorithm. The objects are found in the same order as previously described. (C) Similar to (A) and (B), UMAPs are shown with the cell type annotation. The order is maintained as in (A) and (B) (same parameters are used for each UMAP; resolution = 0.3).

Supplement Fig. 3: Circos plots of DEGs show specific and similar expressed DEGs.
(A) Circos plot showing the ten most upregulated genes in human HFrEF (log2FC), separated for all cell types. Red line indicates the log2FC for human DEGs, while the blue line indicates the log2FC of the corresponding mouse gene. (B) Circos plot similar to (A) illustrates the ten most upregulated genes in mice HFrEF samples in comparison to the regulation of these genes in humans.

Supplement Fig. 4: DEG analysis shows population of shared and cell type-specific genes across cell types in humans and mice.

(A) Upset plot of Human DEGs found in the analyzed cell types. The groups are sorted by their intersection size and plotted on the x-axis of the plot. Additionally the overlapping groups are visualized by a connected dot plot. The size of the DEGs per cell type are shown on the y-axis.

(B) Upset plot of Mouse DEG populations similar to previous plot in (A).

Supplement Fig. 5: GSEA analysis shows regulated pathways upon heart failure in human and mouse endothelial cells.

(A) Dot plot visualizing the ten most significant pathways for terms only to be found regulated in humans, commonly regulated and specific in mice endothelial cells. The size of the dots corresponds to the negative log10 of the Benjamini Hochberg adjusted p-value and the color-code represents the normalized enrichment score (NES), with upregulated pathways shown in red and downregulated pathways in blue. The y-axis depicts the description of the identified term.

(B) Bar plot with mean values for the amount of unique molecular identifiers (UMIs) in the cells for the shown genes. The genes are identified to be dissimilarly regulated between humans and mice for pathways specifically found in humans. (C) Bar graph similar to (B) with mean values for UMIs in cells for genes downregulated in both species for commonly found terms. (D) Bar graph similar to (B) and (C) with mean values for UMIs in cells for genes which are uniquely found to be regulated in terms specifically identified in mice. P-values above the certain groups were calculated by two-sided Student's t-test.

Supplement Fig. 6: Integration of human, mouse and zebrafish scRNA-SEQ prefrontal cortex data of healthy and Alzheimer patients.

(A) UMAP showing human cells (red), mice cells (blue) and zebrafish cells (green) in a common UMAP projection. (B) UMAP with defined clusters according to Seurat's clustering.

Cells of mouse, human and zebrafish origin commingled in the astrocyte cluster (green). Excitatory (red) and inhibitory (green) neurons are mostly present in human data. (C) Bar plot showing cell composition of cell types in human (red), mice (blue) and zebrafish (green) samples. Samples were grouped based on their origin into human, mouse and zebrafish controls from the prefrontal cortex (Healthy) and Alzheimer disease human, mouse and zebrafish (Alzheimer). Cell types were then analyzed for their composition from the previously mentioned groups and plotted. (D) Dot plot depicting the average expression levels and expression proportions in human samples of the top fifteen feature genes for the found cell types. The size of the dot represents the proportion of cells expressing the indicated gene within a cell type, and the color indicates the average expression level of cells. (E) Dot plot depicting the average expression levels and expression proportions in mice samples of the top fifteen feature genes for the found cell types. Similar to (D) the size of the dot represents the proportion of cells expressing the indicated gene within a cell type, and the color indicates the average expression level of cells. (F) Dot plot depicting the average expression levels and expression proportions in zebrafish samples of the top fifteen feature genes for the found cell types. Similar to (D and E) the size of the dot represents the proportion of cells expressing the indicated gene within a cell type, and the color indicates the average expression level of cells.

Figure 1 Click here to access/download;Figure;Fig_1.pdf

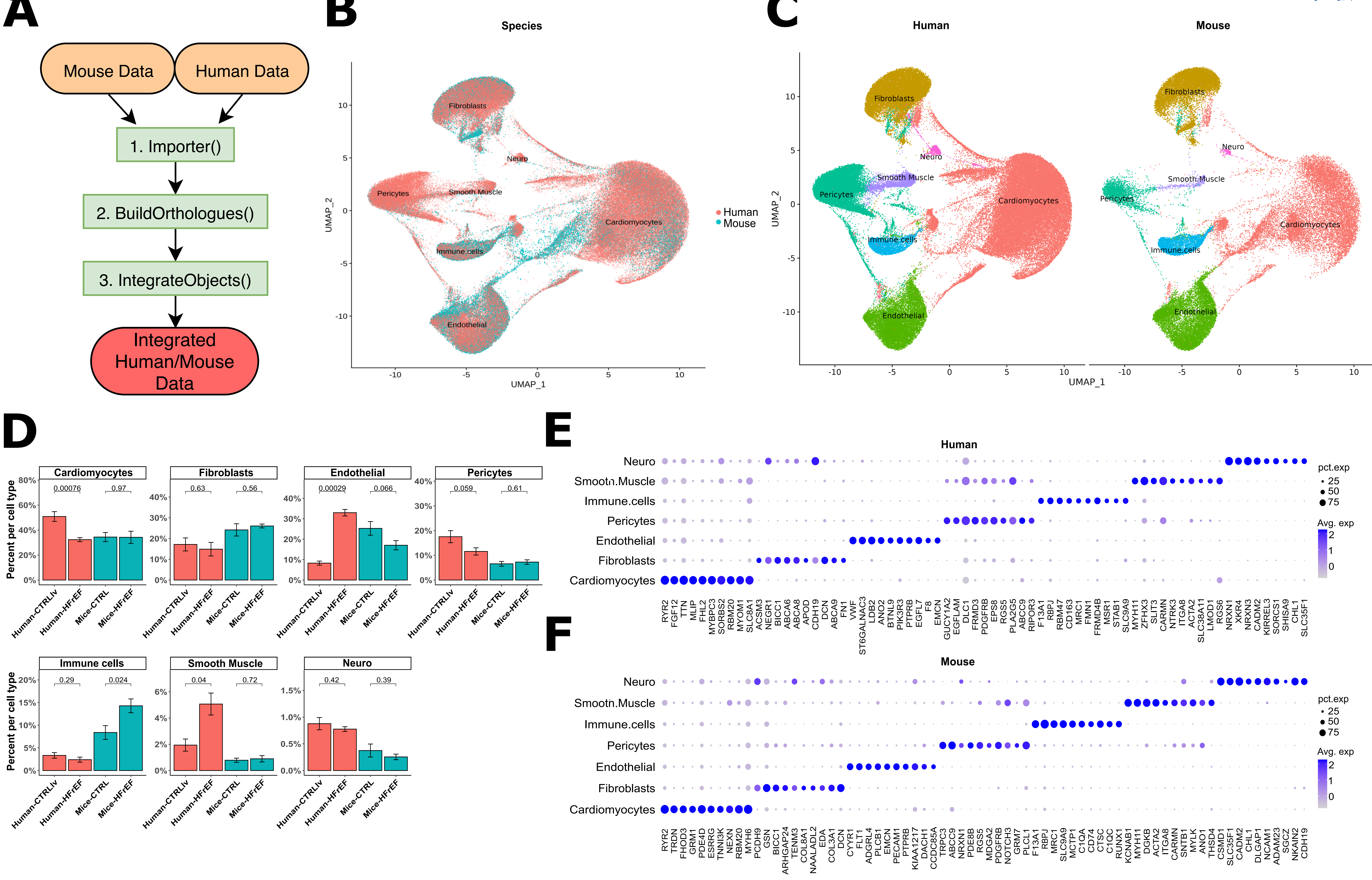

Click here to access/download;Figure;Fig_2_metric.pdf

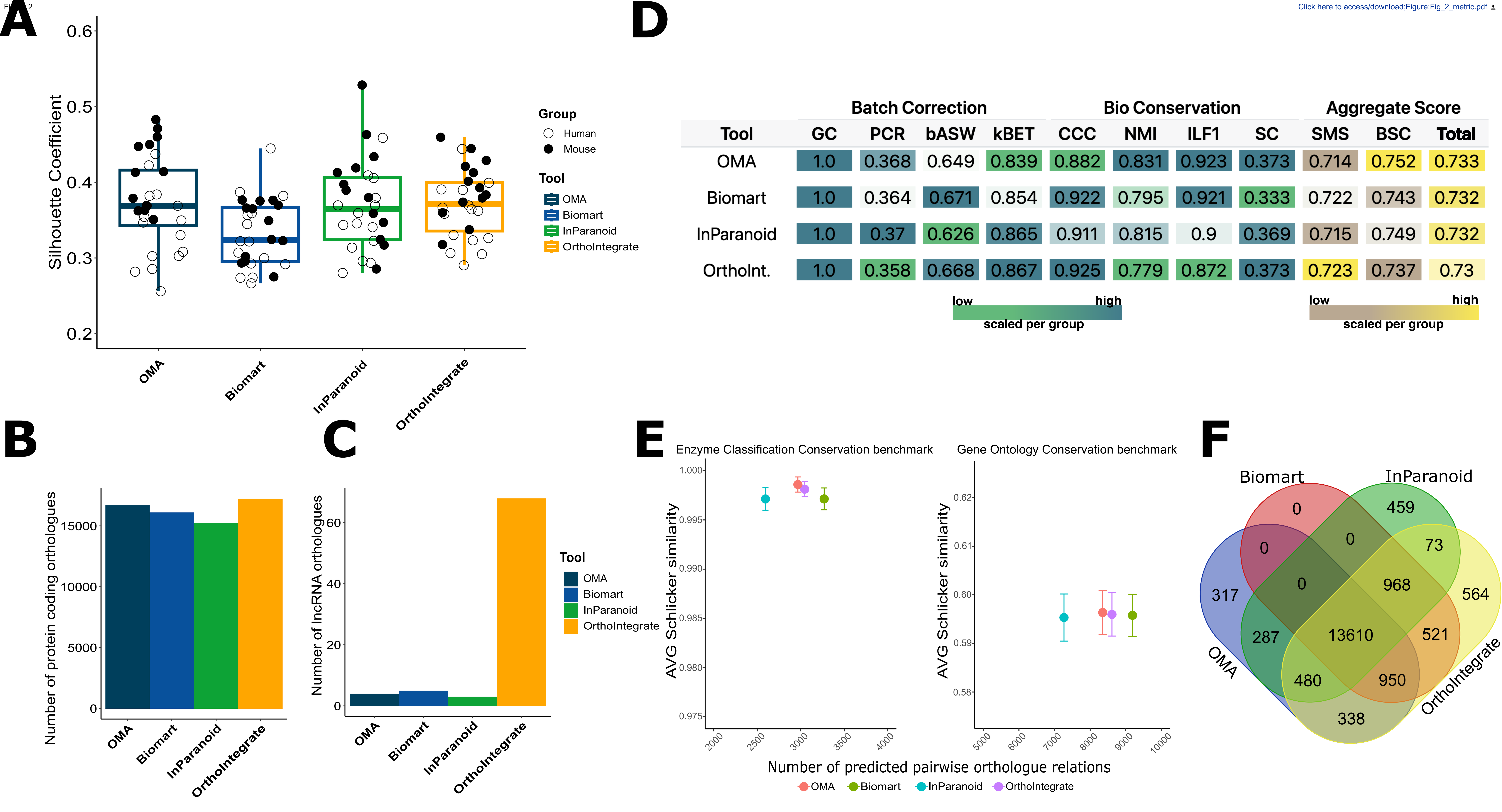

Figure 3

Click here to access/download;Figure;Fig_3.pdf

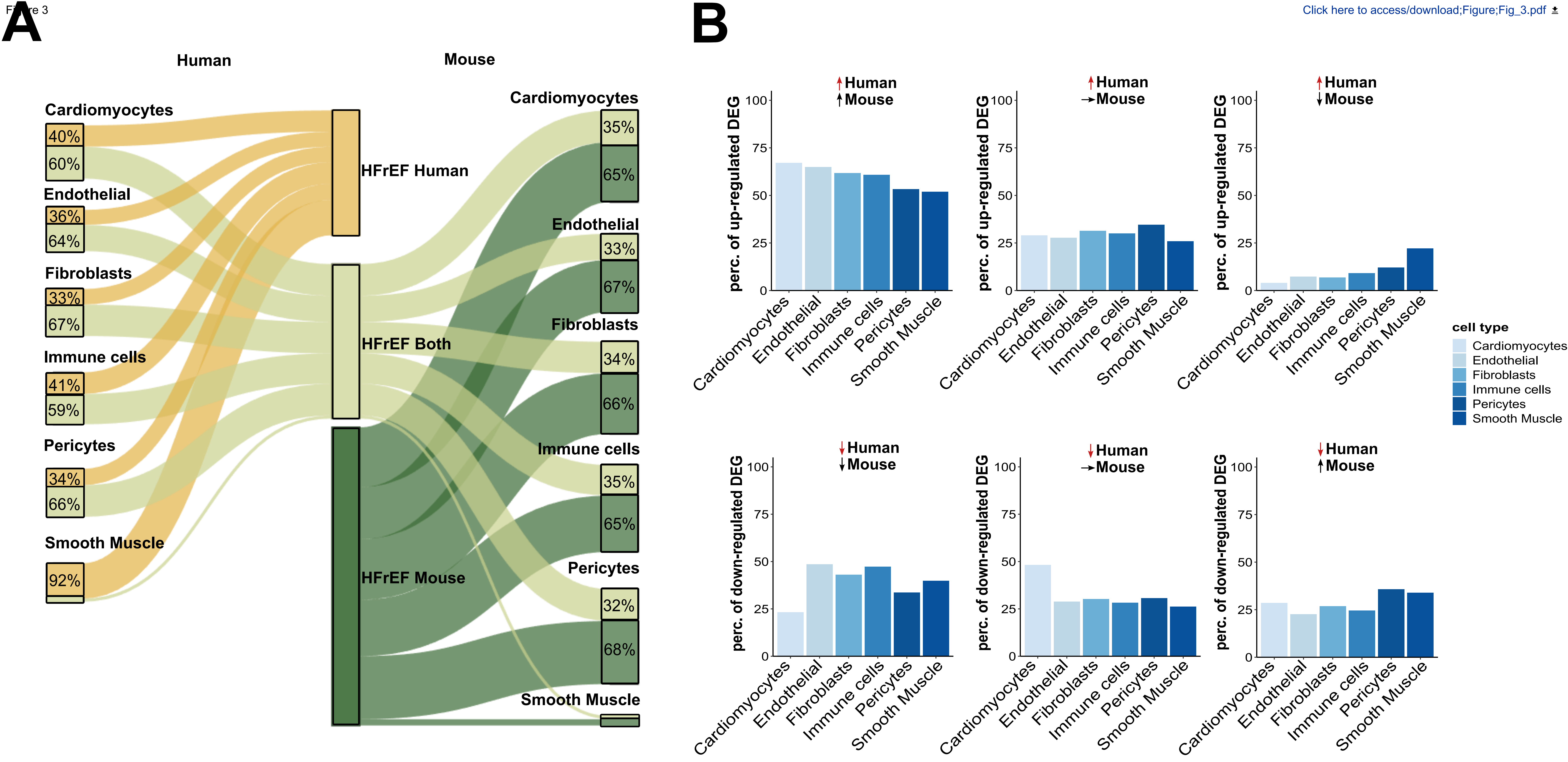

Figure 4

Click here to access/download;Figure;Fig_4_new.pdf

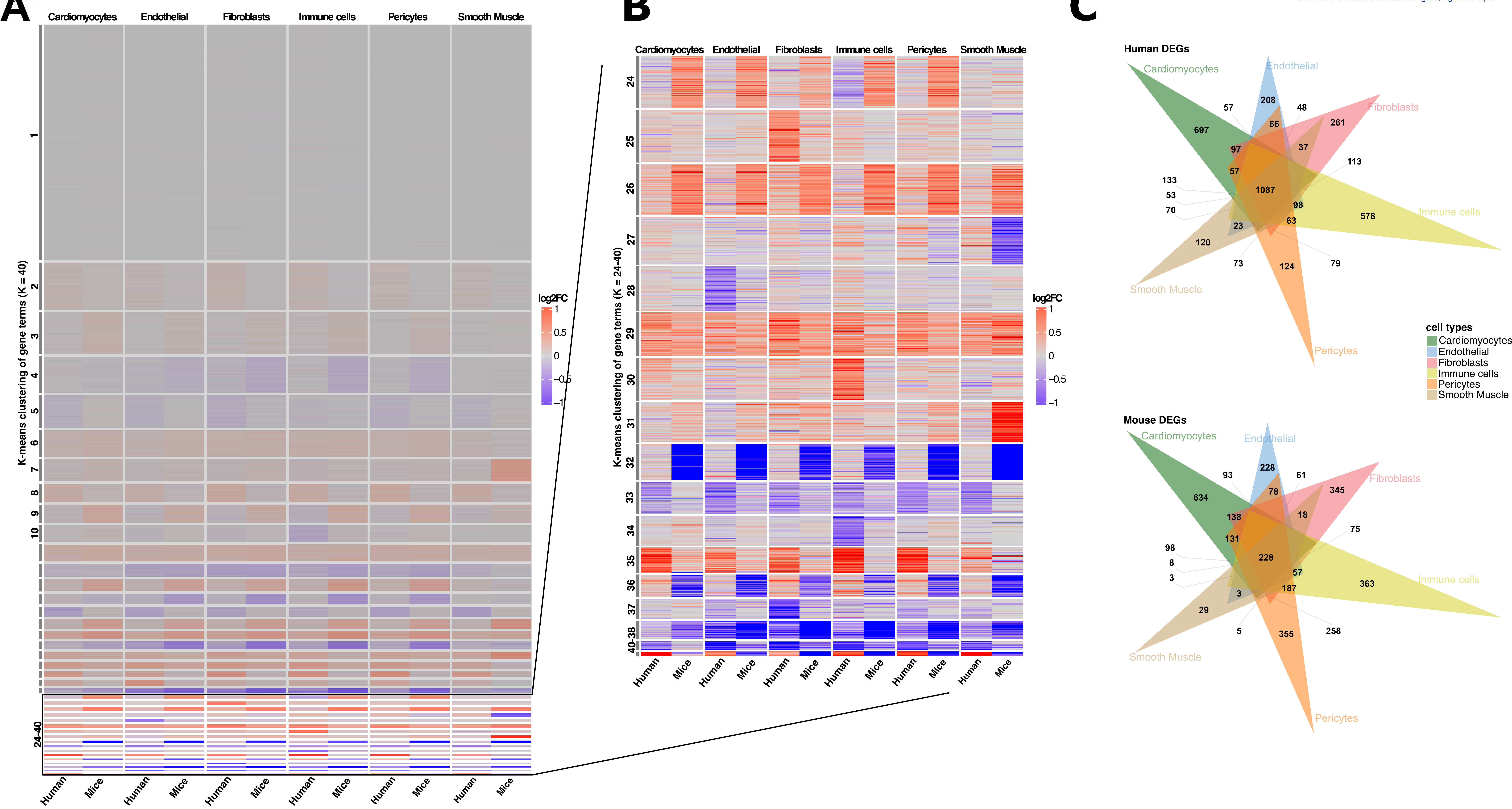

Figure 5

Click here to access/download;Figure;Fig_5.pdf

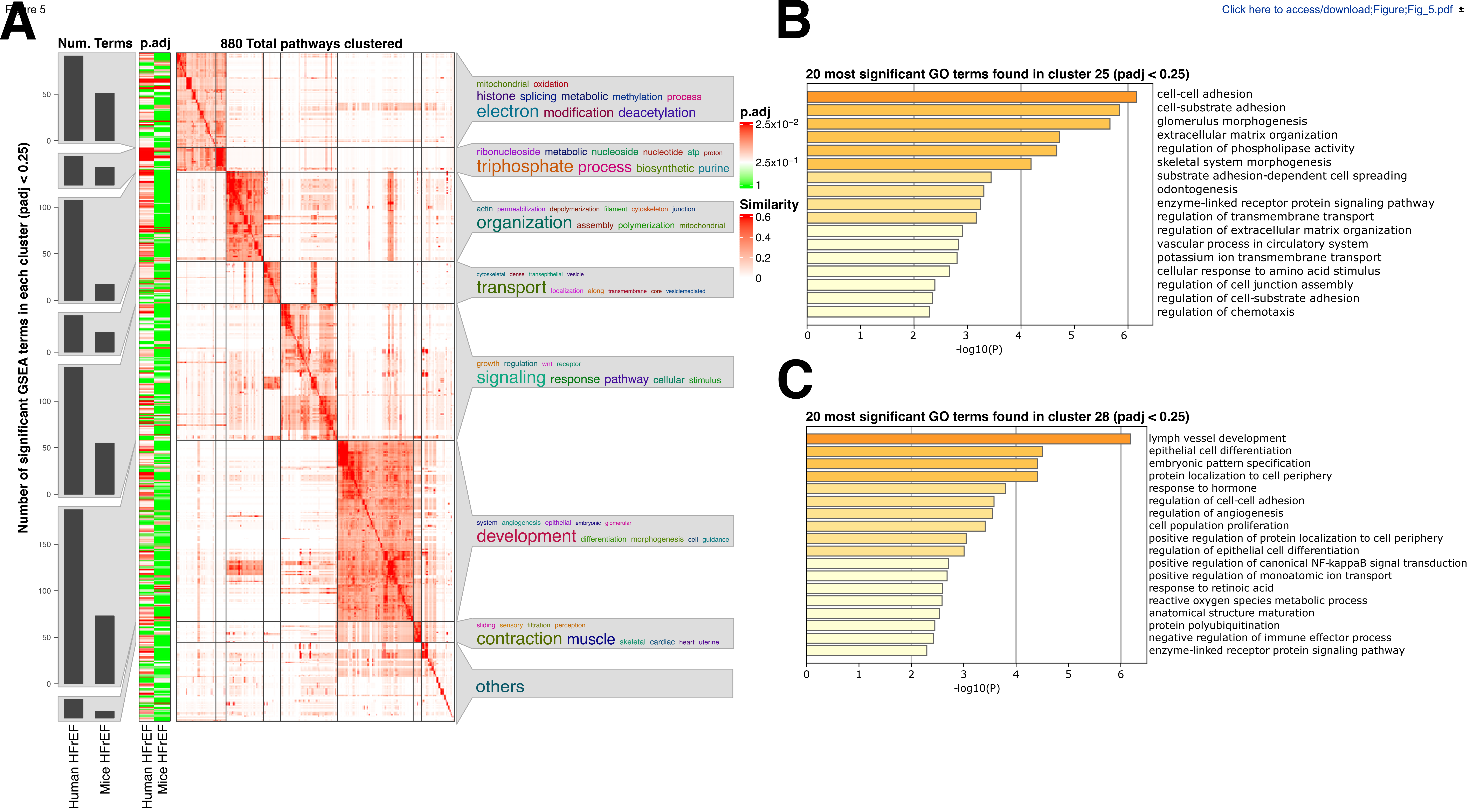

Figure 6

Click here to access/download;Figure;Fig_6.pdf

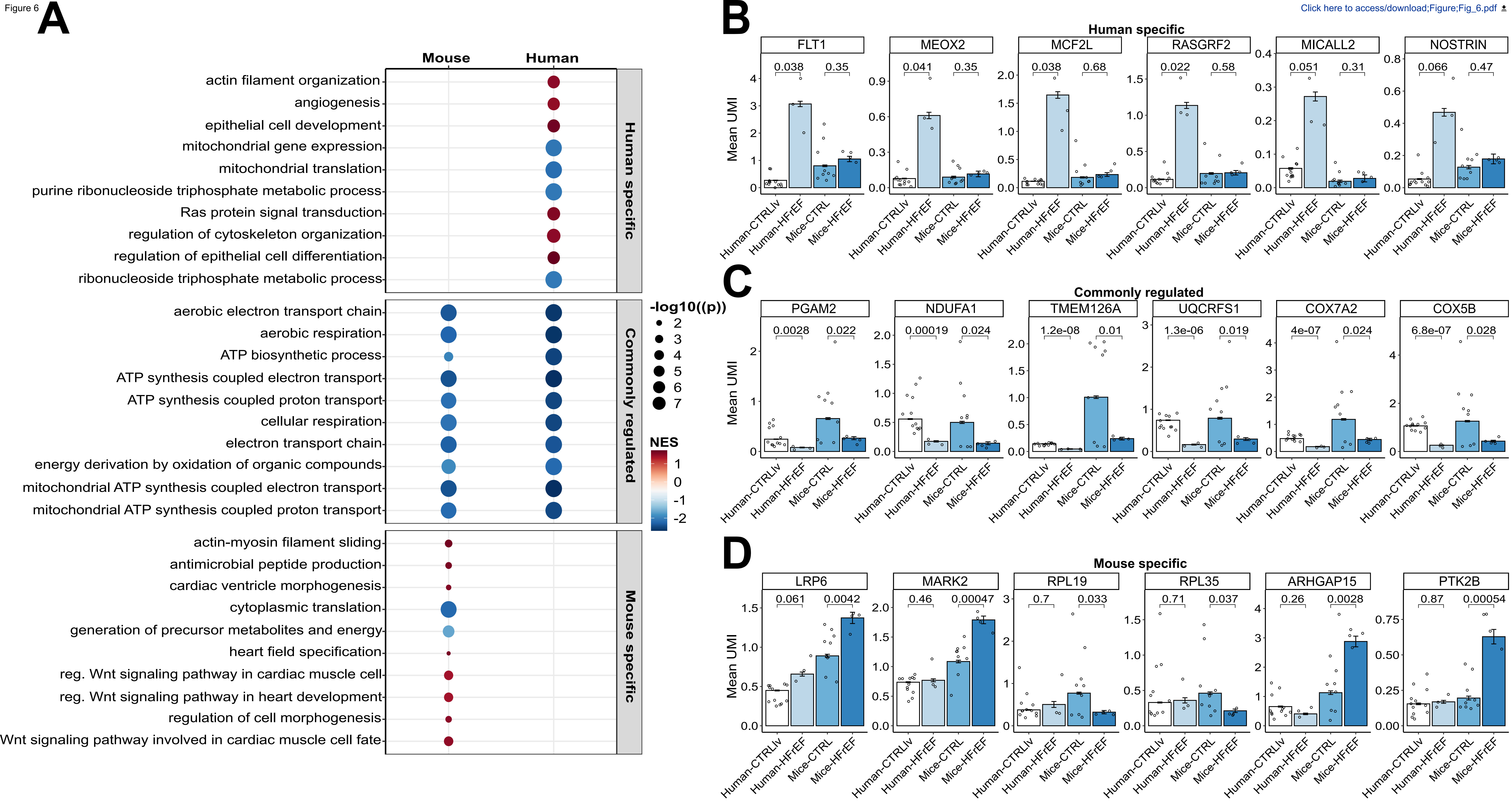

Supplementary Figure 1

Click here to access/download
Supplementary Material
Supp_Fig_1_pipeline.pdf

Supplementary Figure 2

Click here to access/download
Supplementary Material
Supp_Fig_2_UMAPs_comb.pdf

Supplementary Figure 3

Click here to access/download
Supplementary Material
Supp_Fig_3_circos.pdf

Supplementary Figure 4

Click here to access/download
Supplementary Material
Supp_Fig_4_Upset_DEG.pdf

Supplementary Figure 5

Click here to access/download
Supplementary Material
Supp_Fig_5_GSEA_Endothelial.pdf

Supplementary Figure 6

Click here to access/download
Supplementary Material
Supp_Fig_6_Hu_Mi_Ze.pdf

Supplementary Table 1

Click here to access/download
Supplementary Material
Supp_1_PAPER_baseline_charac_Human_Mice.xlsx

Supplementary Table 2

Click here to access/download
Supplementary Material
Supp_2_OrthologueAssignment_stats_mice.xlsx

Supplementary Table 3

Click here to access/download

Supplementary Material

Supp_3_Human Mice DEG analysis HFrEF CTRL.xlsx

Supplementary Table 4

Click here to access/download
Supplementary Material
Supp_4_Human Mice HFrEF GSEA CM.xlsx

Supplementary Table 5

Click here to access/download
Supplementary Material
Supp_5_Human Mice HFrEF GSEA EC.xlsx.xlsx

Supplementary Table 6

Click here to access/download
Supplementary Material
Supp_6_cell_numbers.xlsx

Personal Cover
Click here to access/download;Personal Cover;0.Cover-letter_Gigascience.docx

Fachbereich Medizin der
Johann Wolfgang Goethe-Universität
Frankfurt am Main

Institute of Cardiovascular Regeneration
Goethe-University Frankfurt am Main

University of Frankfurt ·Theodor-Stern-Kai 7 · 60590 Frankfurt

Dr. Edmunds
Editor in Chief
Gigascience

Institute of Cardiovascular Regeneration
Head of Bioinformatic Department:
Dr. David John
Tel.: +49 (0) 69 63 01-87952
E-Mail: john@med.uni-frankfurt.de
Director:
Prof. Dr. Stefanie Dimmeler
Tel.: +49 (0) 69 63 01-66 67
E-Mail: dimmeler@em.uni-frankfurt.de

Date: 17. January 2024

Submission of manuscript

Dear Dr. Edmunds,

Please find enclosed the revised manuscript entitled "**Improved integration of single cell transcriptome data demonstrates common and unique signatures of heart failure in mice and humans**", which we like resubmit to Gigascience for your consideration as a Research Article.

For the revision, we addressed all comments the reviewers addressed in their revision. As requested by the reviewers we provided an **integration of another species and another disease condition**. Therefore, we used published data from Alzheimers disease from human, mouse and zebrafish. We could successfully integrate single cell data of diverge species with the help of OrthoIntegrate. Additionally, we performed **extended benchmarking with other Ortholog database**. As requested, provided detailed benchmarking metrics from the BENGAL paper and performed all reasonable tests from the Orthology Benchmark Service. Detailed gene expression analysis for all celltypes have been added to the GitHub repository and the whole manuscript was restructured to strengthened the research part.

The review process strongly improved the paper quality and therefore we would like to thank the reviewers for their remarks and suggestions.

We believe that our manuscript provides an advanced approach to test the comparability of disease models in mice versus humans, or other species. These insight might lead to the improvement of animal models to test novel therapeutic interventions and in addition may **provide novel insights into so far unexplored human specific disease signatures**.

We hope that the revised manuscript might be considered for external reviewing and be of interest for the readers of Gigascience.

Thank you very much for your generous consideration.

Yours sincerely,

D. John

Dr. David John
