# Supplementary figures and images for: Improved integration of single-cell transcriptome data demonstrates common and unique signatures of heart failure in mice and humans
Source: Gigascience. 2024 Apr 4;13:giae011. doi: 10.1093/gigascience/giae011 (PMC10993718; doi:10.1093/gigascience/giae011)

**A**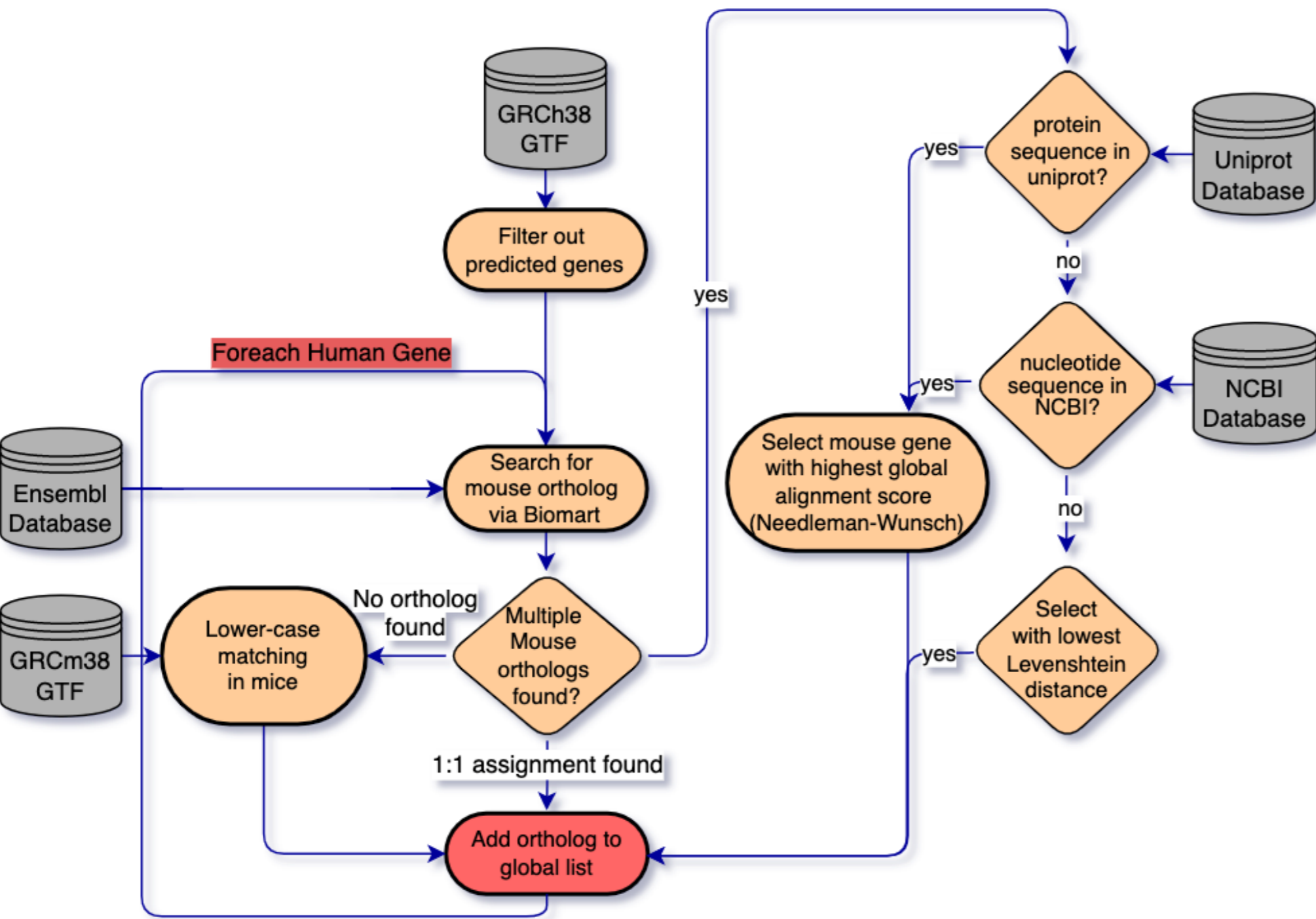**B**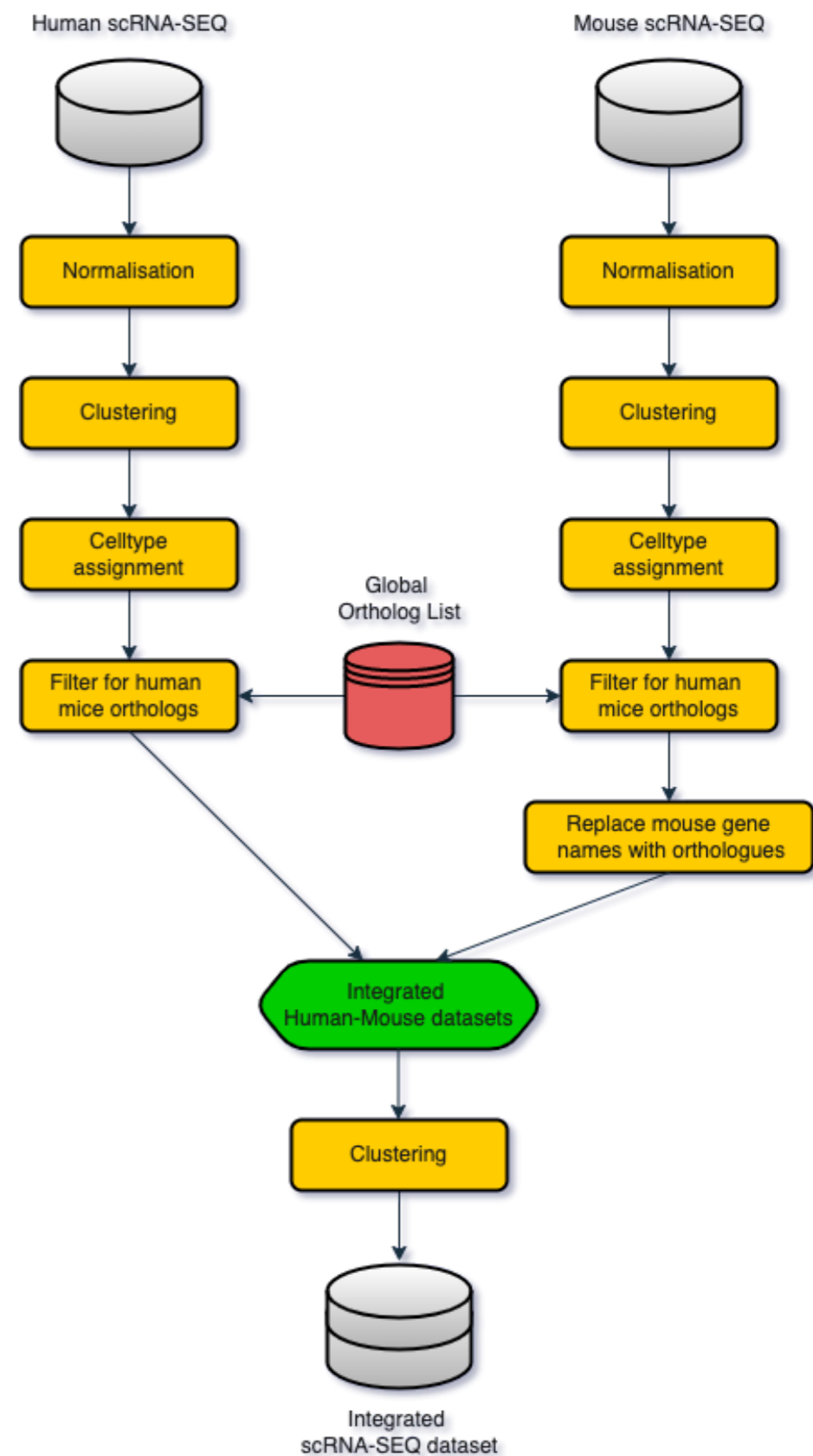

Supplement: giae011_Supplemental_Files [file giae011_supplemental_files.zip › Supp_Fig_1_pipeline.pdf]

**A****Species labeled clustering**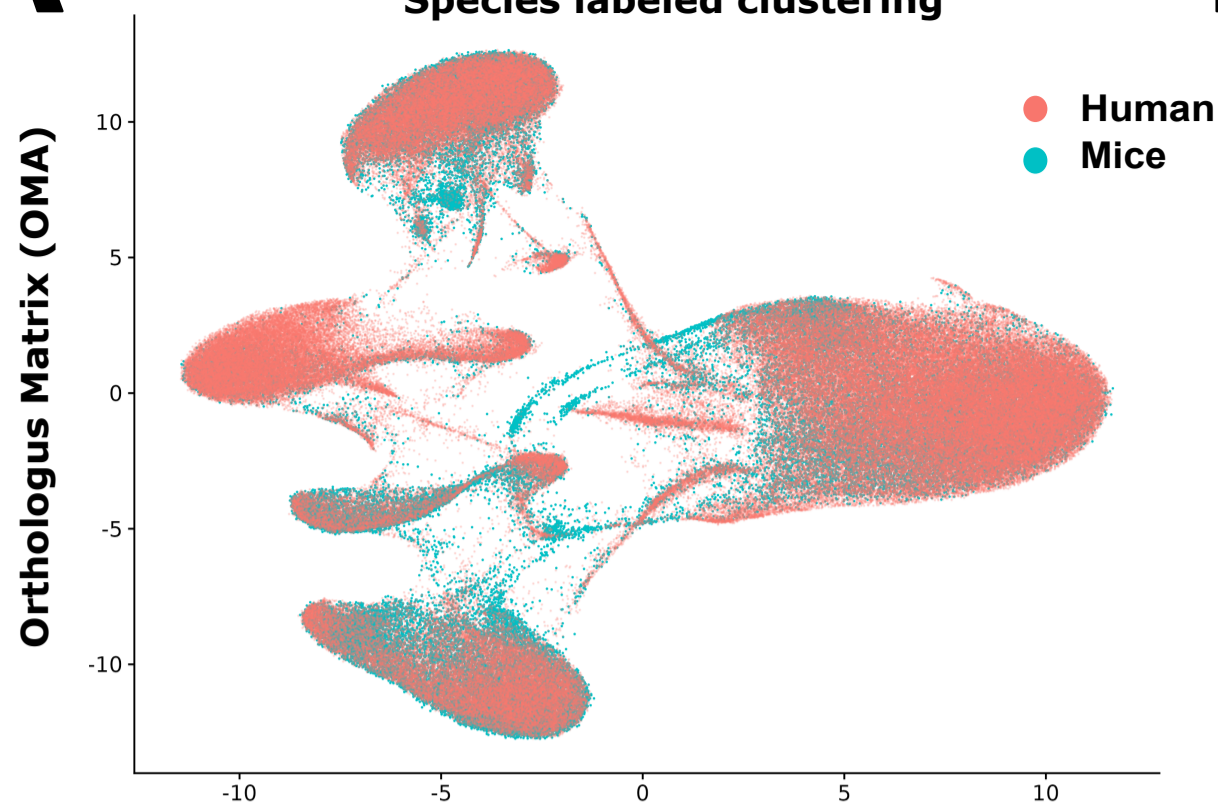**B****Seurat labeled clustering**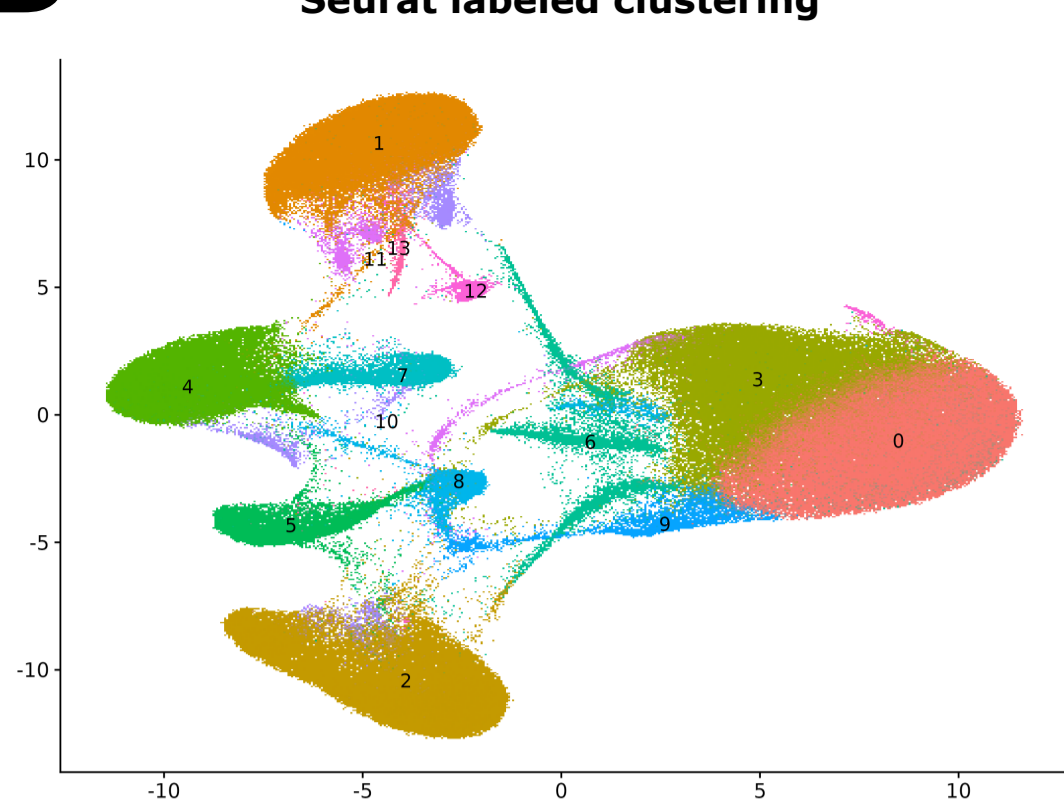**C****Cell labeled clustering**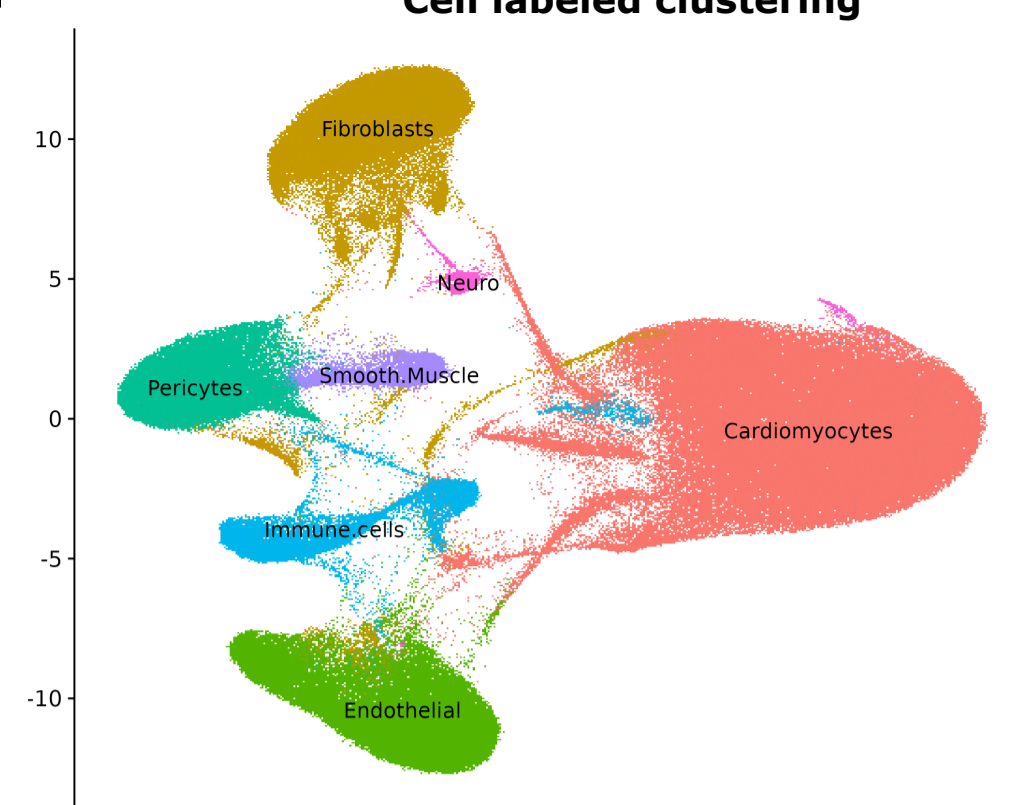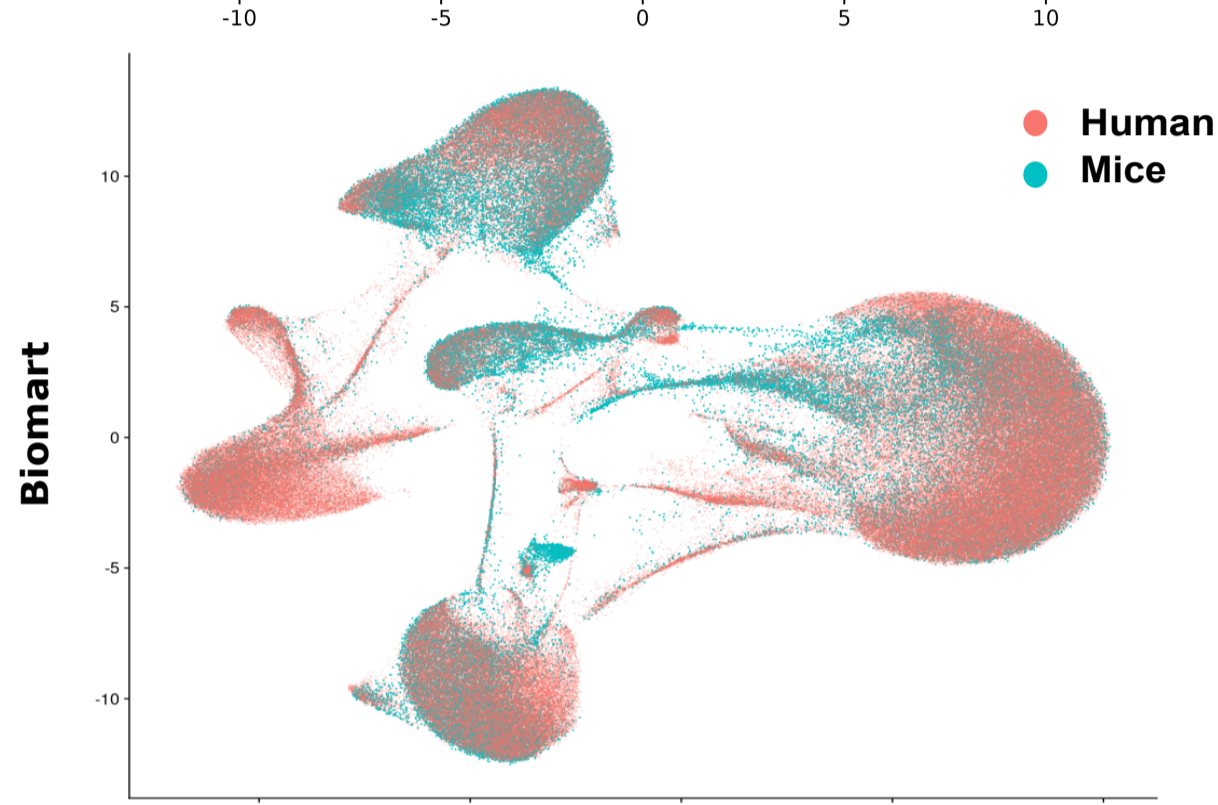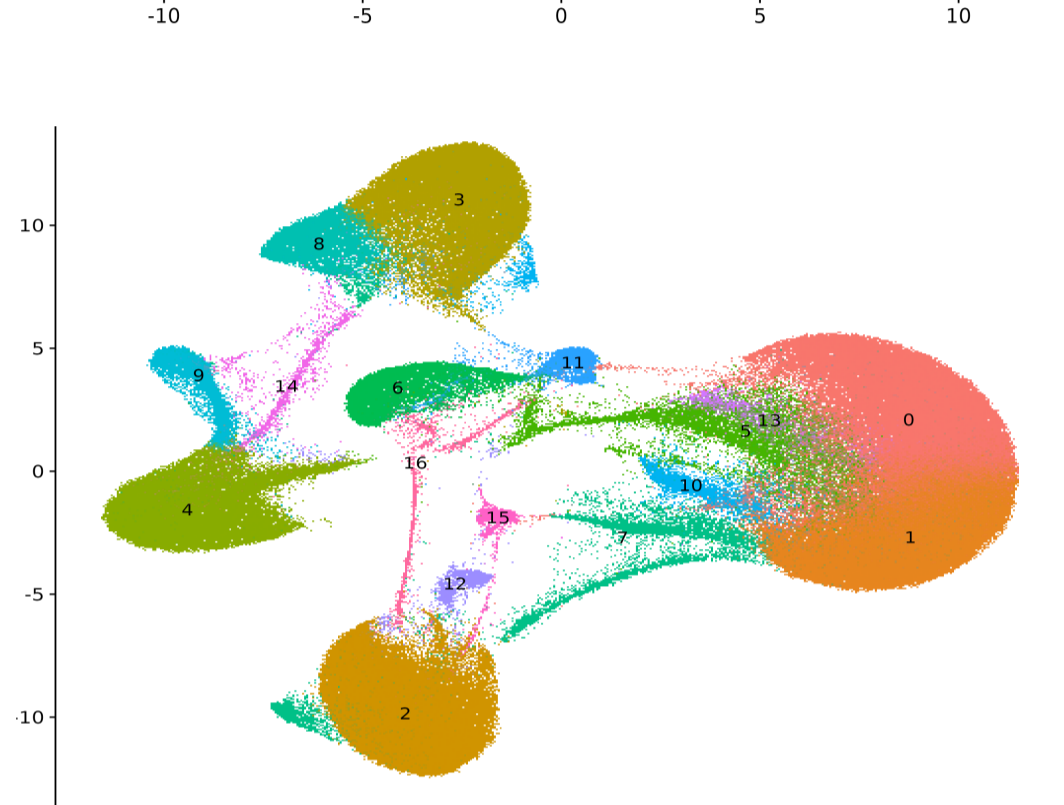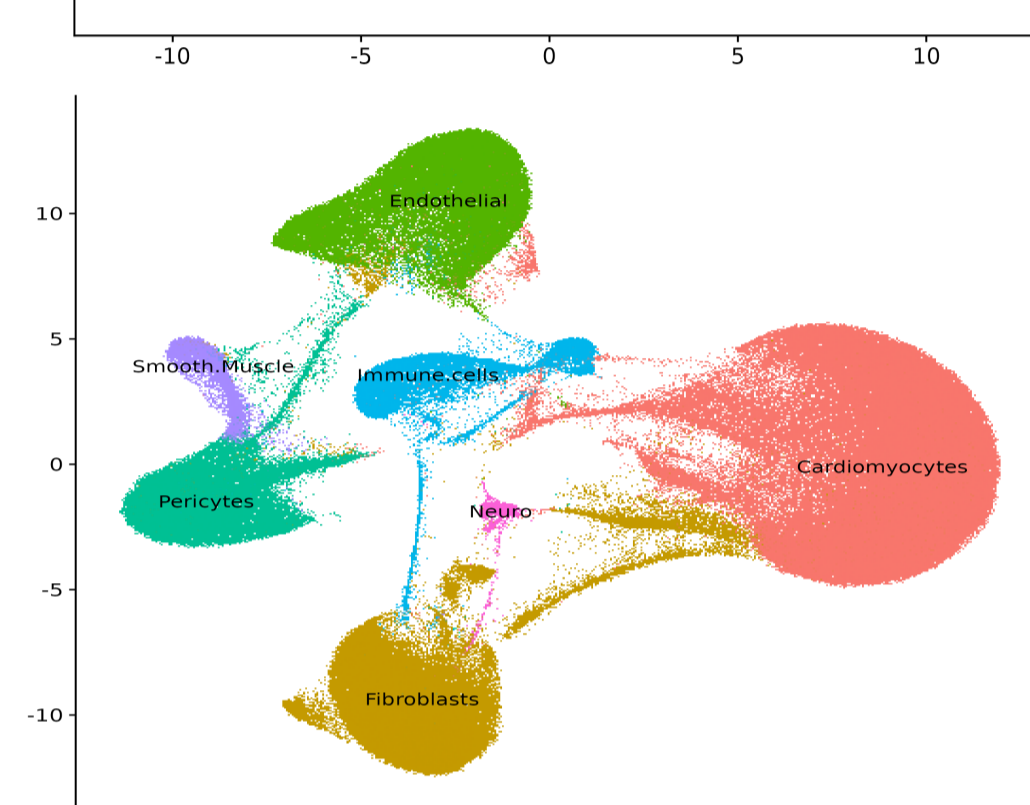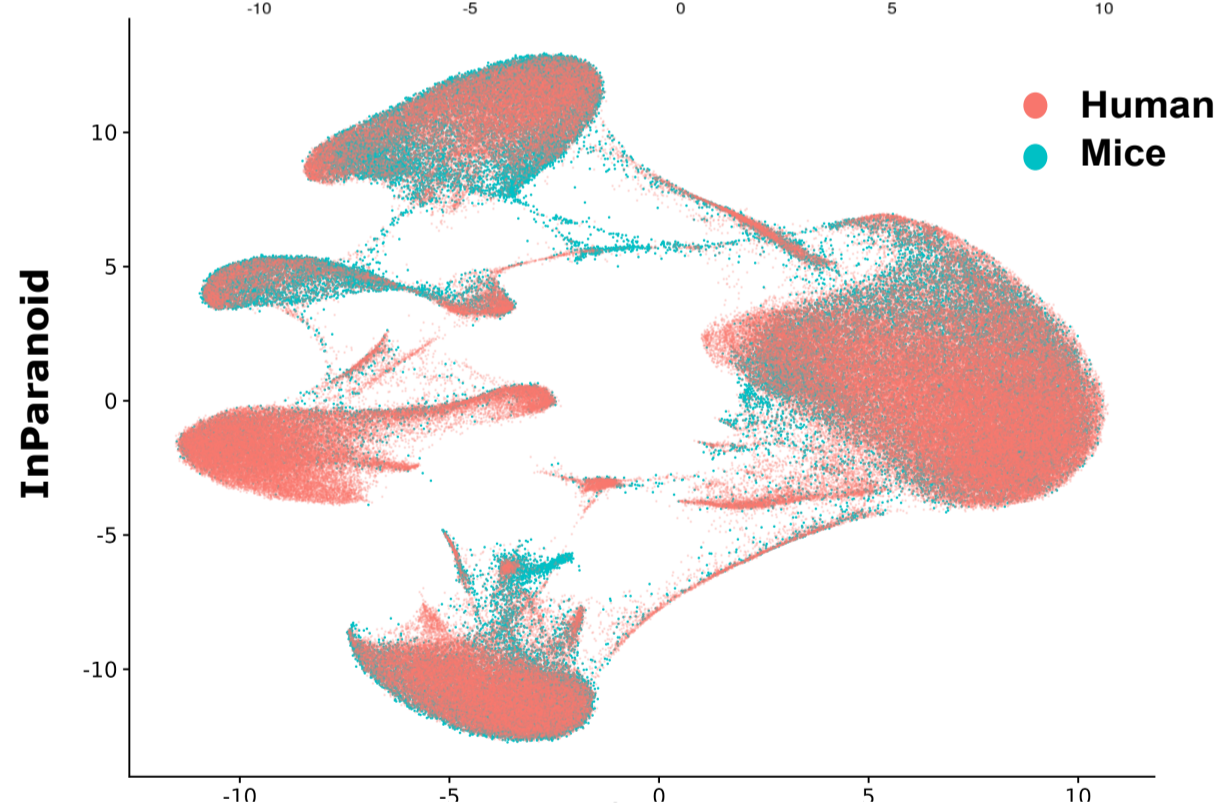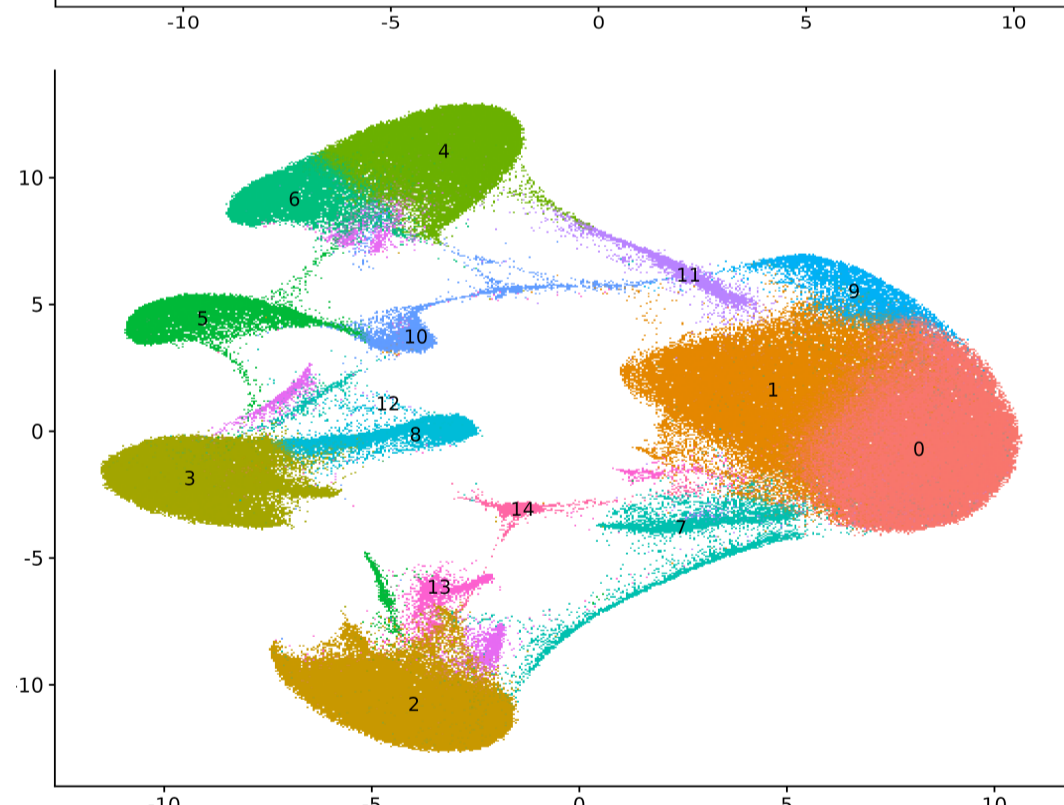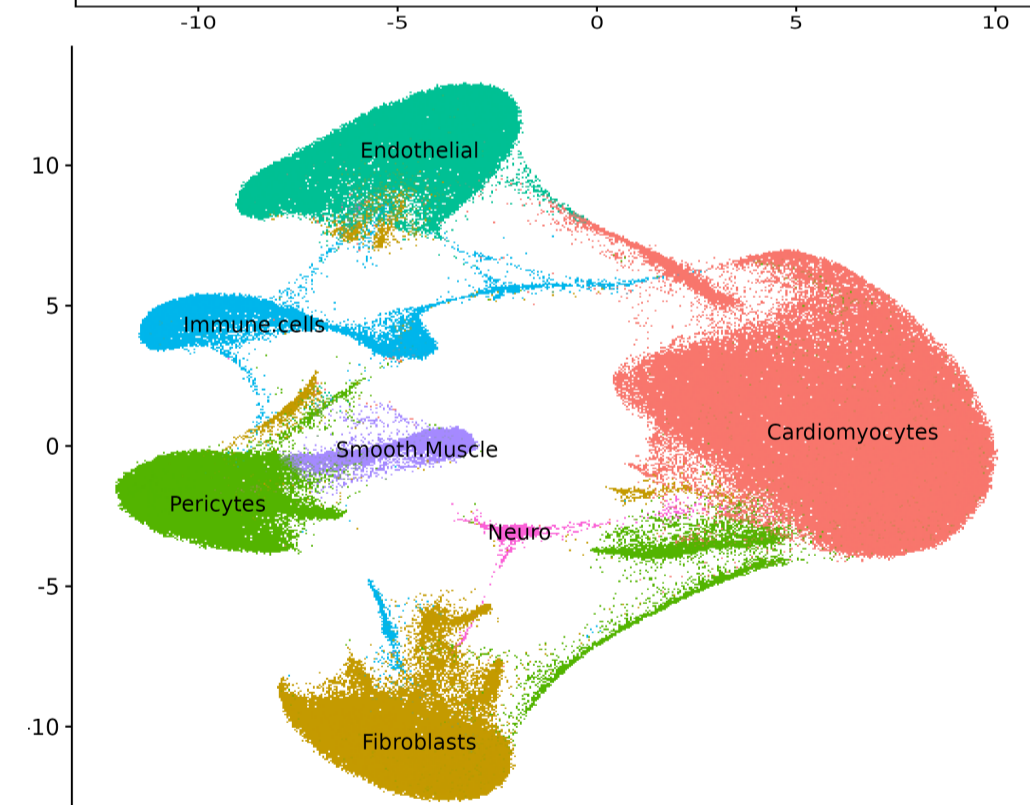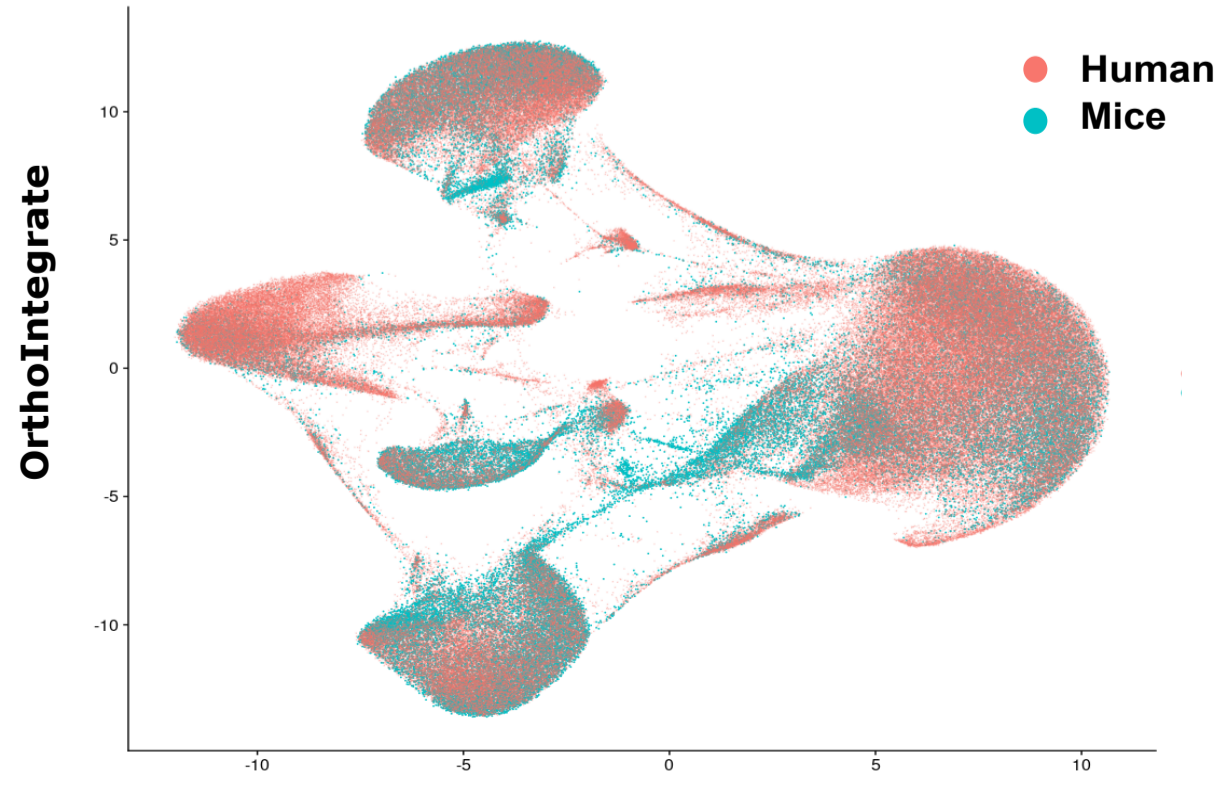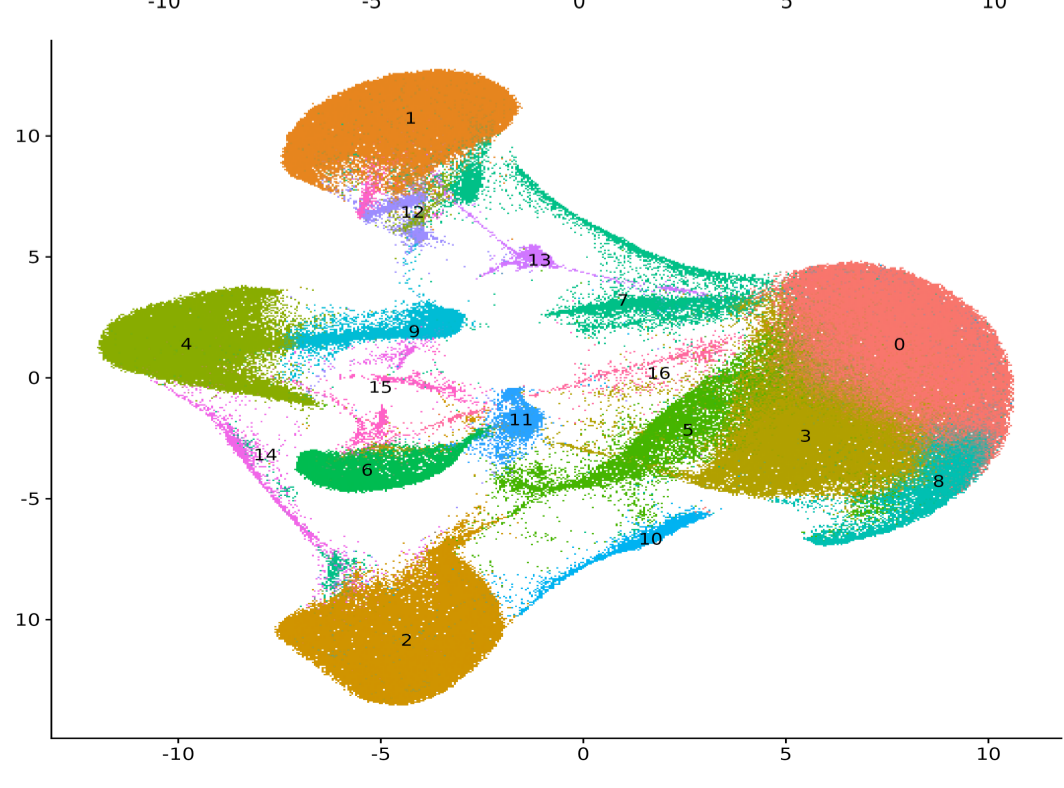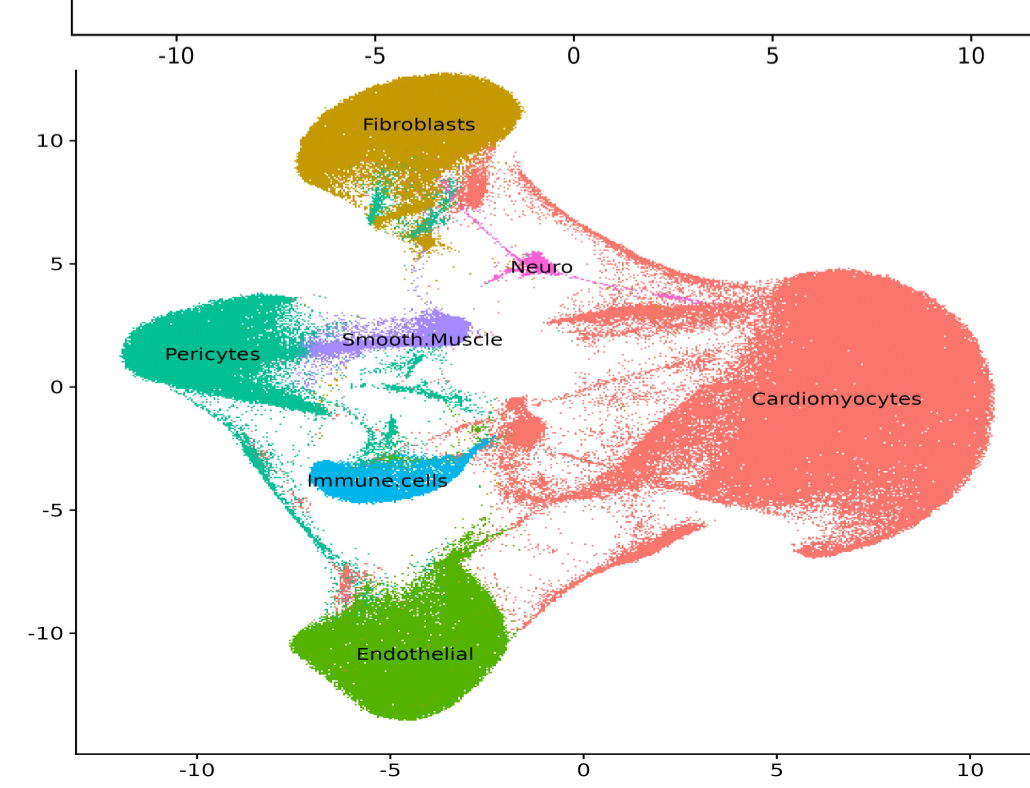

Supplement: giae011_Supplemental_Files [file giae011_supplemental_files.zip › Supp_Fig_2_UMAPs_comb.pdf]

A

Fold Change HUMAN DEGs HFrEF vs CTRL

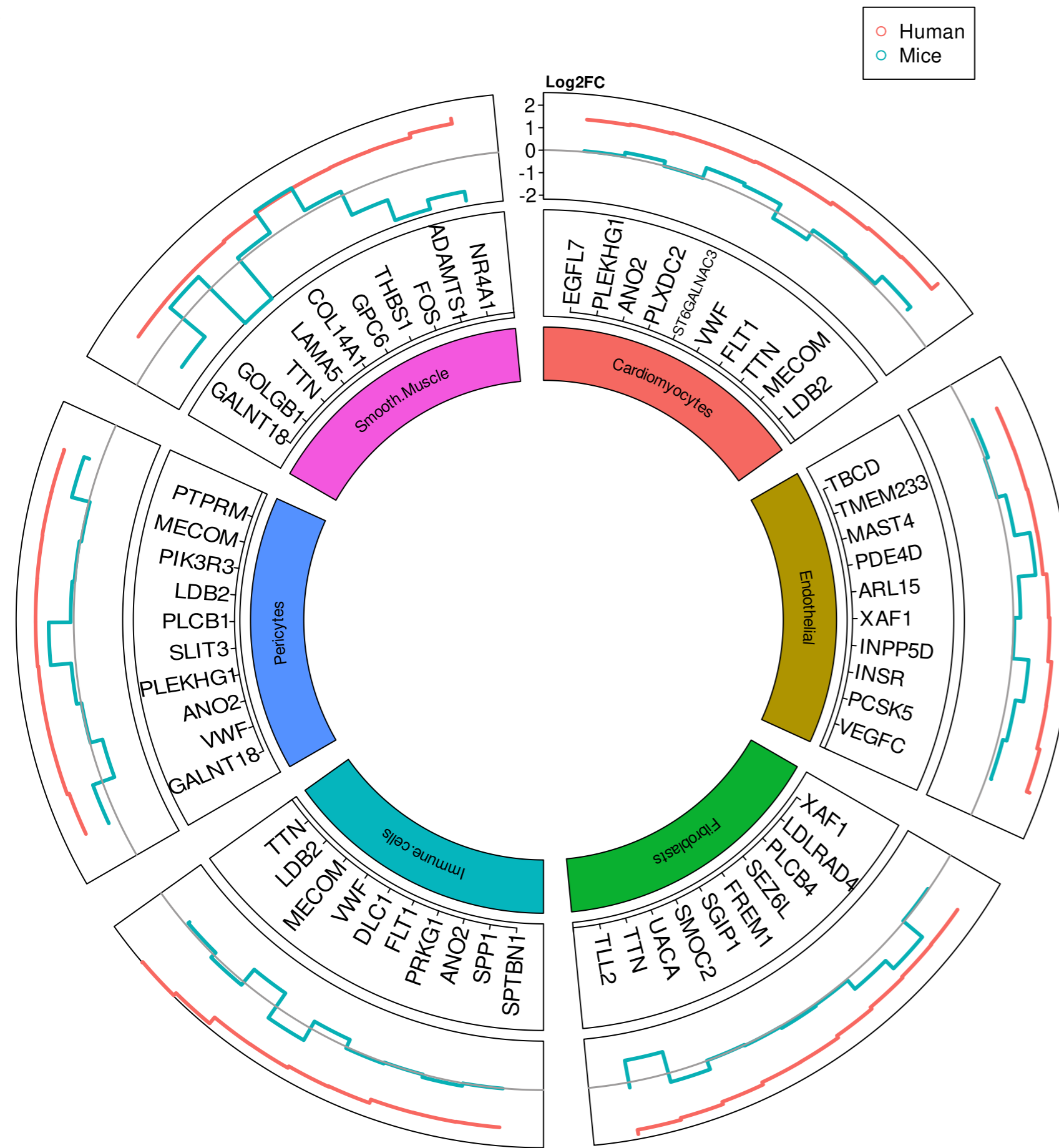

B

Fold Change MICE DEGs HFrEF vs CTRL

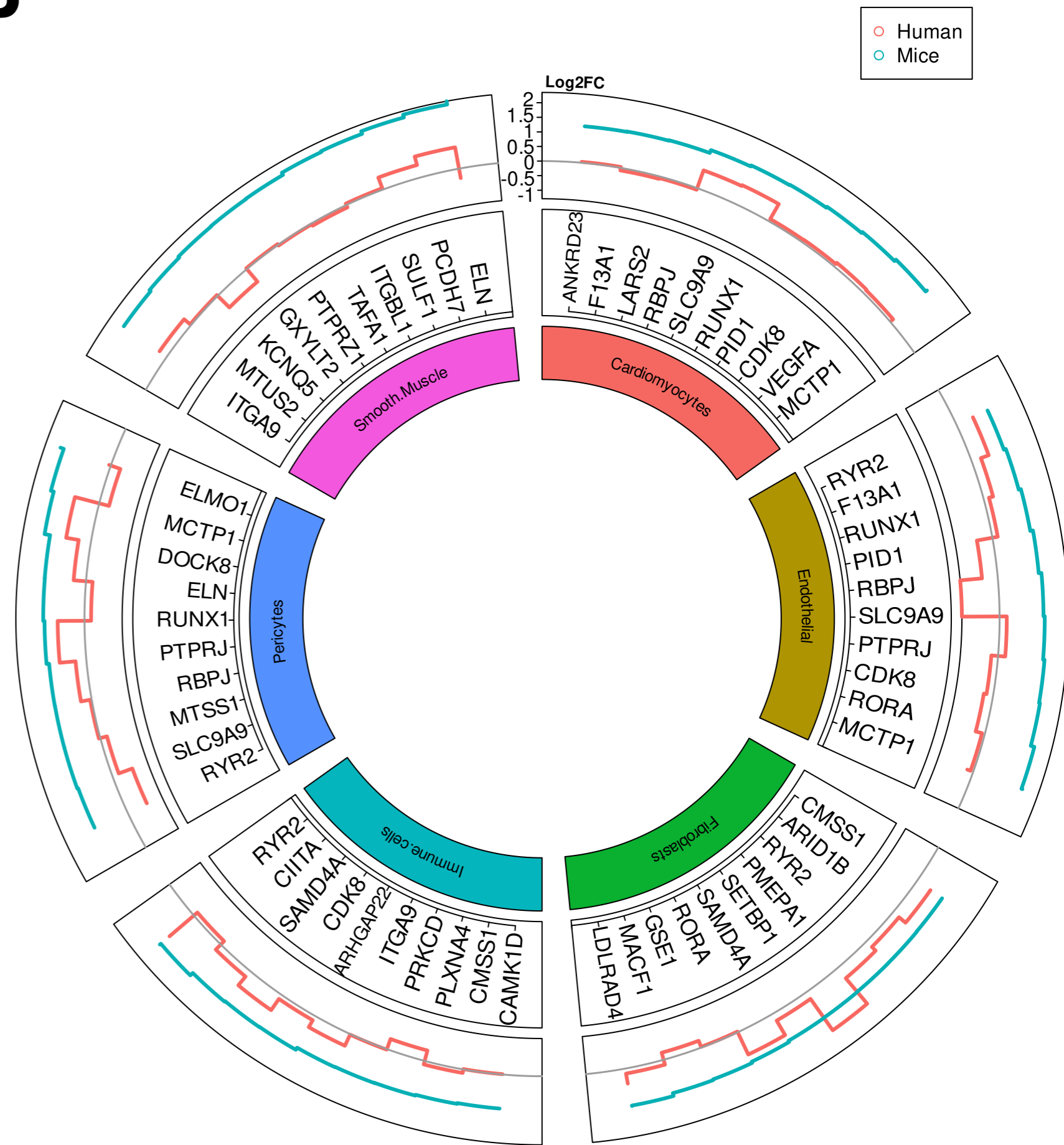

Supplement: giae011_Supplemental_Files [file giae011_supplemental_files.zip › Supp_Fig_3_circos.pdf]

# A

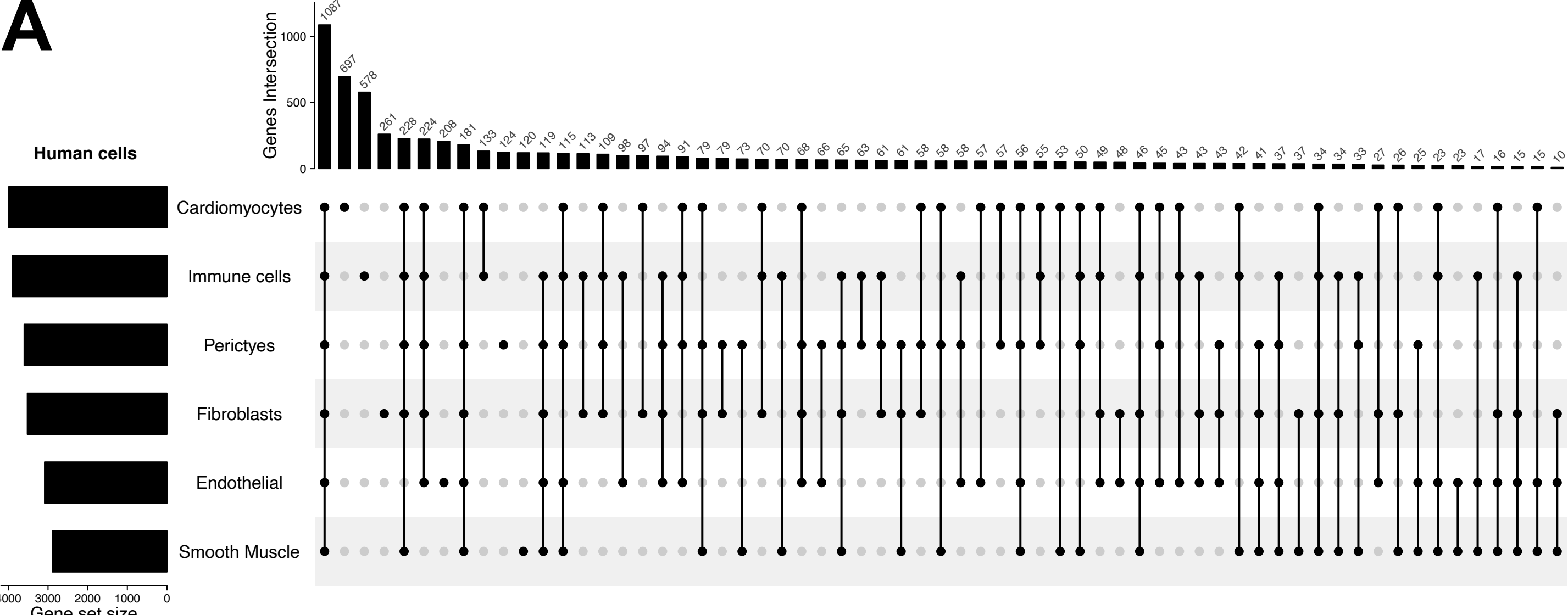

# B

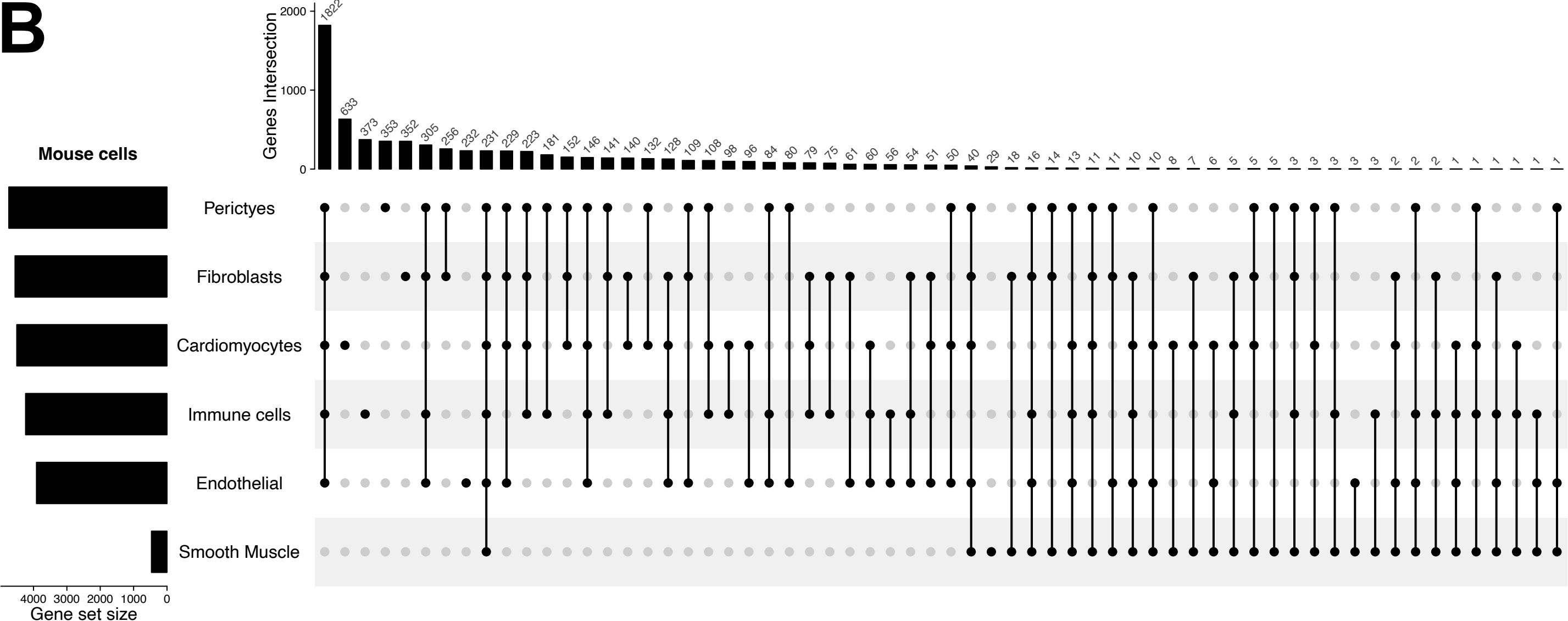

Supplement: giae011_Supplemental_Files [file giae011_supplemental_files.zip › Supp_Fig_4_Upset_DEG.pdf]

A

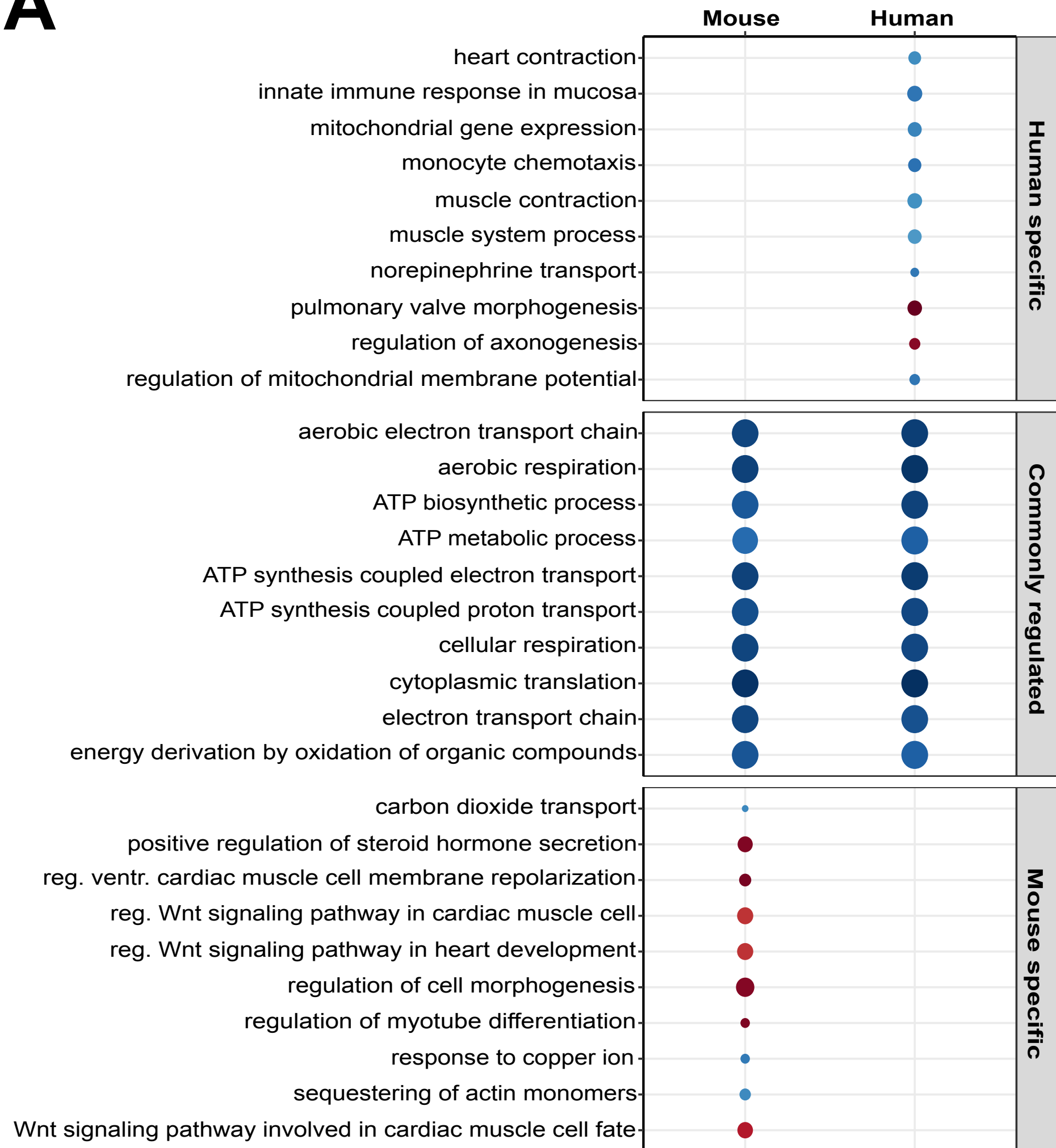

B

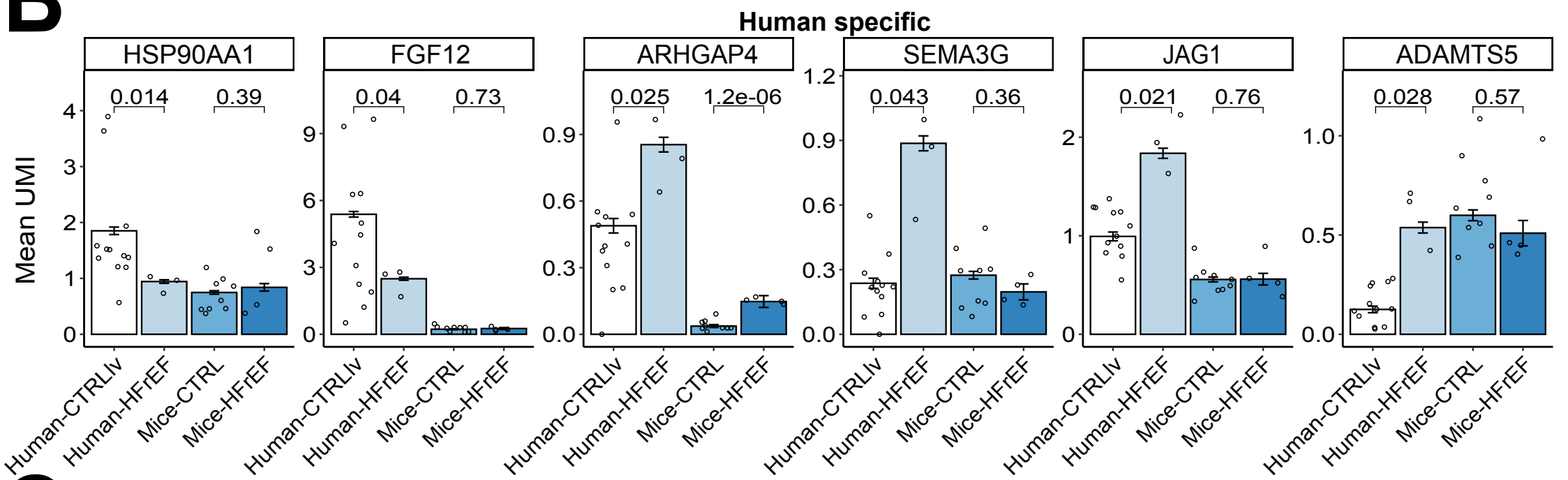

C

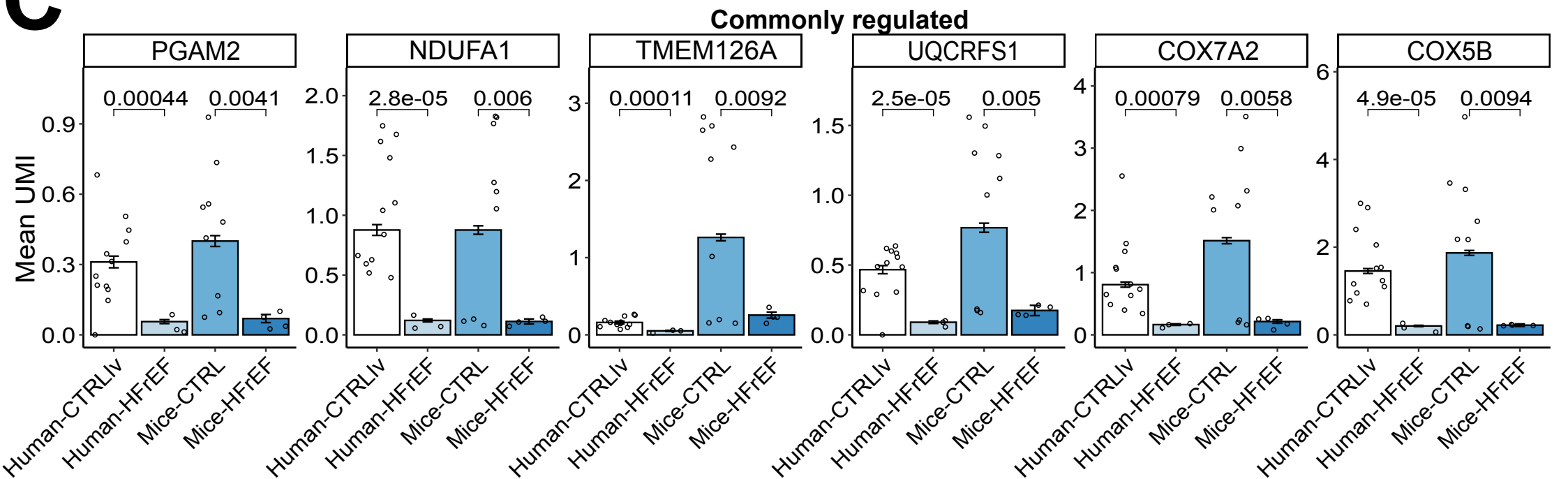

D

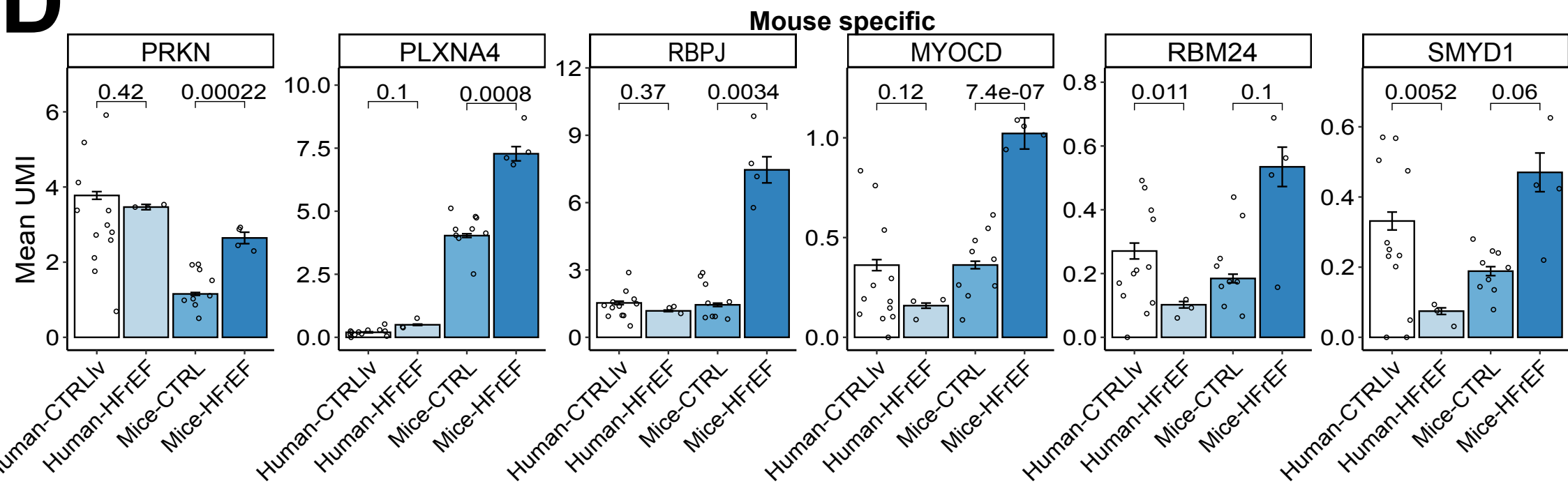

Supplement: giae011_Supplemental_Files [file giae011_supplemental_files.zip › Supp_Fig_5_GSEA_Endothelial.pdf]

**A**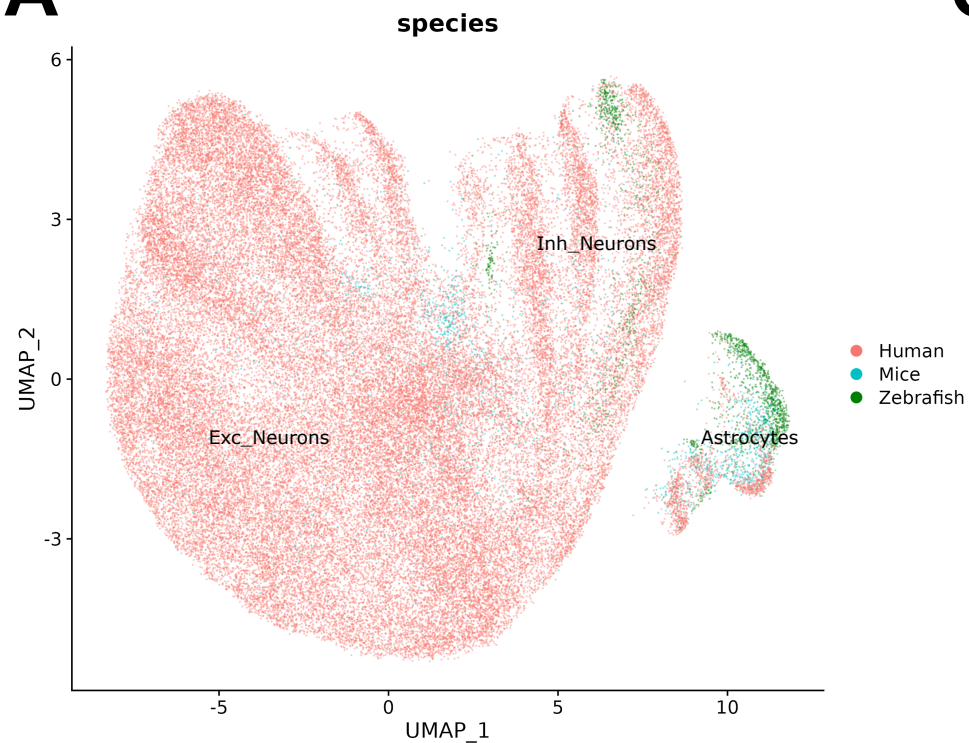**B**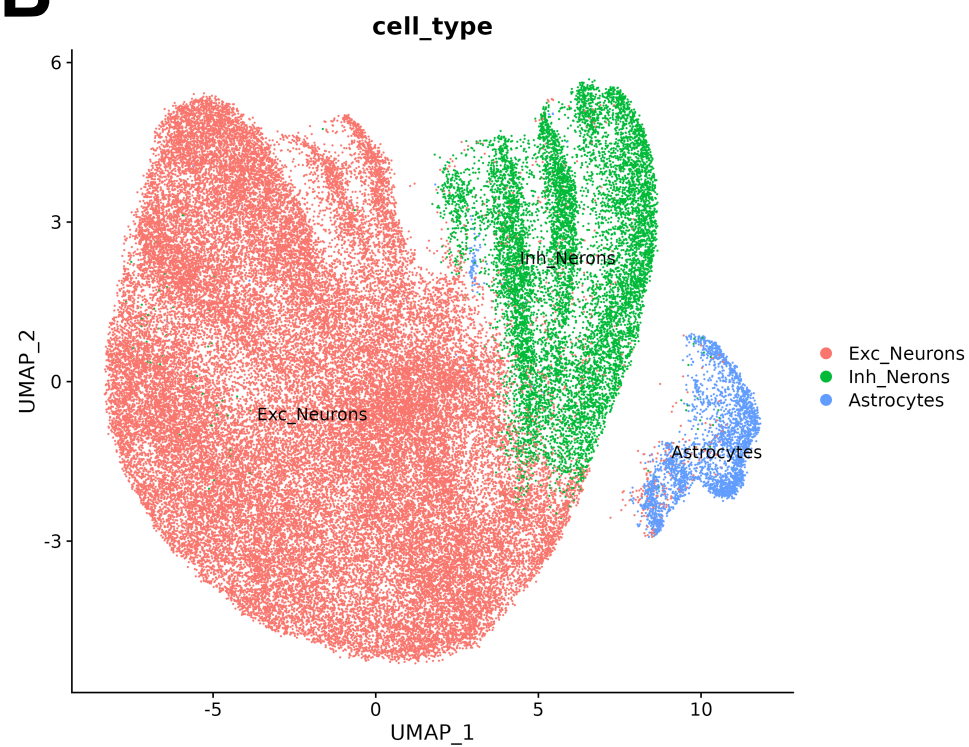**C**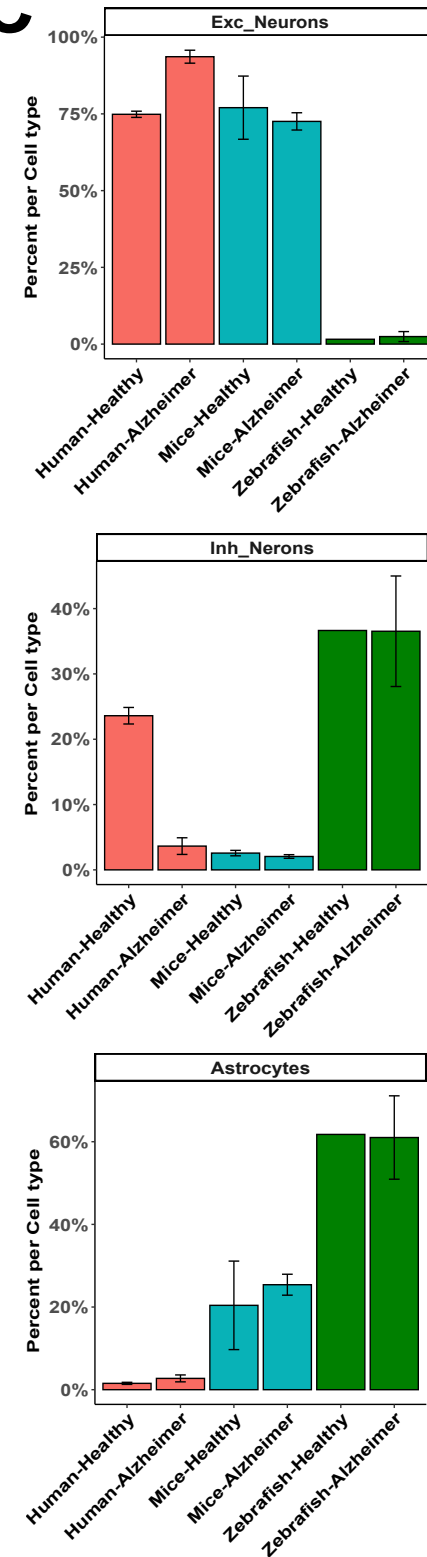**D**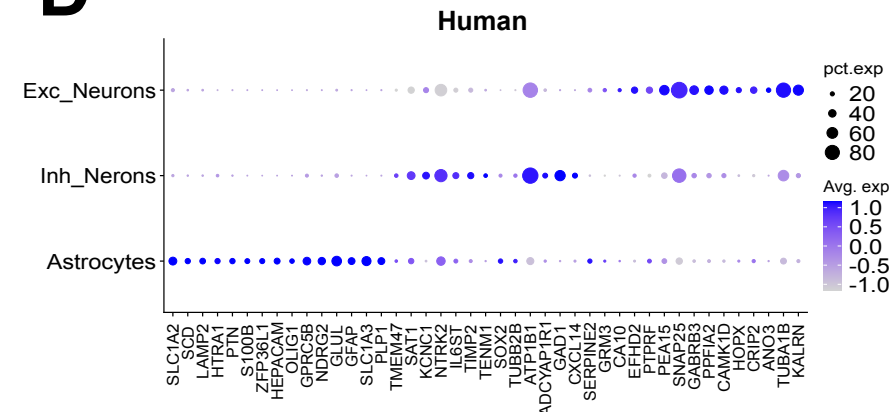**E**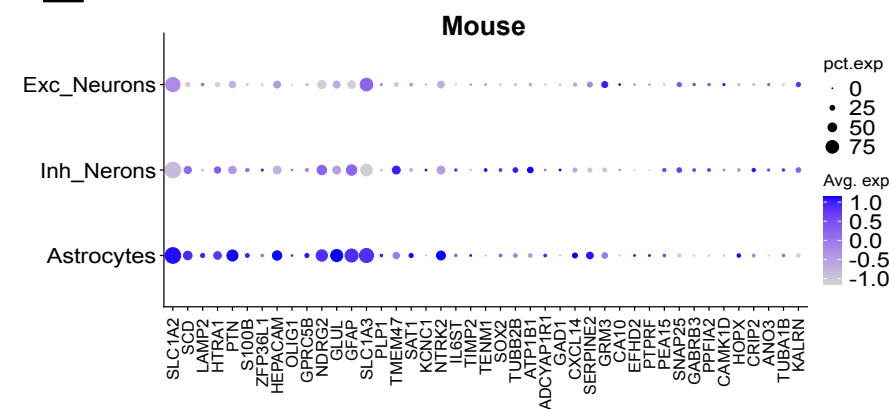**F**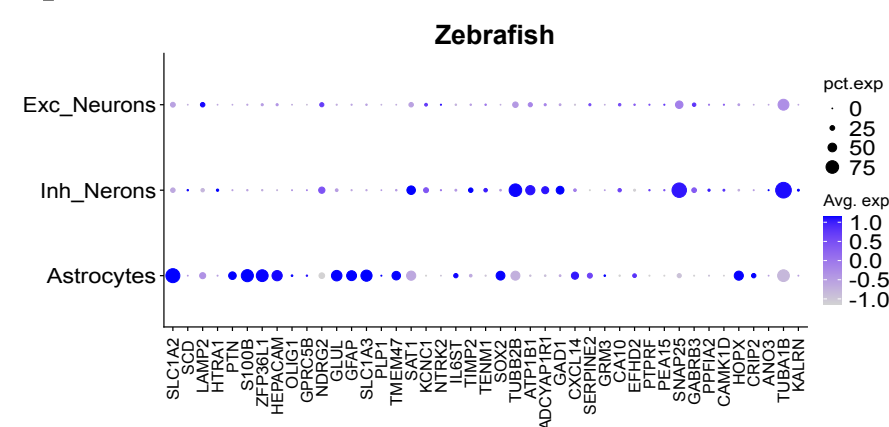

Supplement: giae011_Supplemental_Files [file giae011_supplemental_files.zip › Supp_Fig_6_Hu_Mi_Ze.pdf]
