# Supplementary material for: Improved integration of single-cell transcriptome data demonstrates common and unique signatures of heart failure in mice and humans
Source: Gigascience. 2024 Apr 4;13:giae011. doi: 10.1093/gigascience/giae011 (PMC10993718; doi:10.1093/gigascience/giae011)
Supplement: giae011_Response_to_Reviewer_Comments_Original_Submission [file giae011_response_to_reviewer_comments_original_submission.pdf]

Author's Response To Reviewer Comments

Response to the reviewers
In general, we think that the review process strongly improved the paper quality and therefore we would like to thank the rev [illegible]
suggestions. Te answers to the reviewer questions and remarks can be found below the comment, marked in green.

Reviewer1 :

A few points need to be addressed before publishing

1. The authors utilized the Needleman-Wunsch algorithm to generate one-to-one orthologs between human genes and mouse [illegible]
using this algorithm compared to other algorithms i.e., SAMap uses BLAST?

We appreciate the reviewer's comment. In our study, we focused on aligning sequences with multiple orthologues listed for o [illegible]
ensemble database. To ensure accurate comparisons, we opted for global alignments since the reference and query sequence [illegible]
gene set of potential homologues, our aim was to select the orthologue with the highest overall agreement with the reference [illegible]
chosen because they are less likely to show false homology [1].
On the other hand, local alignments, as employed in SaMap (Blast), have advantages in identifying homologues in species wi [illegible]
between evolutionarily distant species [2]. Their purpose is to pinpoint similar sequencing regions within larger sequences. H [illegible]
overall sequence conservation of sequences with similar lengths [1].
SaMap is specifically designed for defining homologs between species with higher evolutionary distances, but it comes with co [illegible]
focused on comparing well-annotated species with high evolutionary conservation, and we achieved the best results with the [illegible]
OrthoIntegrate. However in order to provide functionality for more distant species we added a parameter to select between g [illegible]
assignment of one-to-many orthologues. The function BuildOrthologues() now provides an argument alignment_type, which [illegible]
thereby switching between the Smith-Waterman and the Needleman-Wunsch algorithm.

2. The authors have shown the application of OrthoIntegrate in the context of heart failure between mice and humans. Could [illegible]
more example of using OrthoIntegrate in other disease conditions or between other species to show the versatility of OrthoIn [illegible]

We thank the reviewer for this reasonable comment. As suggested, we applied OrthoIntegrate to another species and anothe [illegible]
obtained single cell data from human mouse and zebrafish for healthy and Alzheimer's conditions and integrated it with Ortho [illegible]
summarized in Suppl. Fig. 6 and an additional paragraph was added to the discussion.

"To further demonstrate the functionality of OrthoIntegrate, we integrated scRNA-SEQ data from human [41], mouse [54] ar [illegible]
under alzheimer condition. Besides the evolutionary distance between these species, we could jointly cluster different cell typ [illegible]
A-C) and detect commonly expressed marker genes within these cell clusters (Suppl. Fig 6 D-F)."

3. To assess the quality of clustering after integration, the authors calculated silhouette coefficients/scores and found that int [illegible]
in an improved clustering performance. Could the authors include more benchmarking metrics to assess the performance of [illegible]
methods? The authors could consider metrics like the species mixing score used by BENGAL (Song et al., 2022, biorxiv; https [illegible]
Genomics/BENGAL)

We would like to thank the reviewer for drawing our attention to the Bengal Paper [3] . We applied all their benchmarking me [illegible]
results can be seen in Figure 2D. Additionally we added the following paragraph to the discussion section.

"We demonstrated the usability of combining cross-species single cell data by using data sets of human and mouse heart failu [illegible]
In order to evaluate the species mixing and the biological conservation of different integration methods, we applied certain m [illegible]
which were also suggested by Song et.al [3–5]. The results are summarized in Figure 2D. We found that most batch correctio [illegible]
OrthoIntegrate.
For biological conservation scores, we demonstrate that some metrics, like the "cell cycle conservation" are improved by usin [illegible]
the variance caused by different cell cycle states of the cells is conserved via OrthoIntegrate. Other parameters like the NMI- [illegible]
example is strongly influenced by the cell type labeling [3], which was focused only on main cell type groups in these dataset [illegible]
subpopulation or mixed cell type population clusters. In other words, subclusters of different cell types were not annotated in [illegible]
numbers of features that are included in OrthoIntegrate, the clustering might be more diverged, likely by species specific nor [illegible]

which are not included in the other databases. Therefore, the more divergent clustering, due the increased number of feature
the broad cell type labeling might explain the slightly reduced NMI scores. “

4. Miscalling of figures: silhouette coefficients are shown in Supp_Fig_4 rather than Suppl_Fig_3.

We changed the text accordingly and added Supp_Fig_4 to the main manuscript as Fig 2.

5. Some information on the used datasets in the manuscript has been shown in supplementary table 1, but it's still a bit conf
and human HFrEF datasets come from. I am not exactly sure, but I presume HFrEF datasets are from E-MTAB-13264? This in
explicitly in the method section.

We changed the text accordingly and added detailed information regarding the origin of the samples. Additionally, we added
the Alzheimer datasets.

Reviewer2:

- [ ] 1. Ortholog identification has long been a critical and essential step for many comparative, evolutionary, and functional g
performance of an orthology inference method, there are some gold standards available for benchmark testing, such as the Q
(https://orthology.benchmarkservice.org). Whether OrthoIntegrate outperforms other methods should be comprehensively b
metrics, rather than relying solely on the silhouette coefficient score from a heart single-cell RNA sequencing (scRNA-seq) da

According to the reviewer suggestions, we incorporated the Quest Orthology Benchmark Service and tested the 4 ortholog da
OrthoIntegrate has the second highest Gene Ontology Conservation score and the second highest Enzyme Classification score
was achieved with InPara.
We tried to perform other tests as well. But since we are only comparing two species and these tests require multi species ev
perform all tests from the Quest Orthology Benchmark Service. The results from the Orthology Benchmark Service were incor

- [ ] 2. According to the authors' integration pipeline, both human and mouse scRNA-seq data are individually clustered to as
further integrated with orthologous genes for clustering to assign new labels. How do the labels for each cell and each cell typ
integration approach? Does cell type assignment become more reasonable after the integration? The authors should demonst
genes for clustering improves the accuracy of cell type assignment. The silhouette coefficient score is not a direct metric for a
influenced by biological factors. For example, in Supplementary Table 3, the silhouette scores of mouse-HFrEF samples gener
consistently higher than those by OrthoIntegrate, which is opposite to the control groups and human-HFrEF samples.

In order to assign cell type, we manually applied previously established marker genes (Tombor et al. 2021) to assign the hum
we used singleR, to transfer cell labels from the human to the mouse samples, based on published marker genes.
Furthermore, we applied all benchmarking metrics from the BENGAL paper [3] to our pipeline in order to validate celltype ass

We added the following paragraph to the discussion:

“We demonstrated the usability of combining cross-species single cell data by using data sets of human and mouse heart failu
In order to evaluate the species mixing and the biological conservation of different integration methods, we applied certain m
which were also suggested by Song et.al [3–5]. The results are summarized in Figure 3D. We found that most batch correctio
OrthoIntegrate.
For biological conservation scores, we demonstrate that some metrics, like the “cell cycle conservation” are improved by usin
the variance caused by different cell cycle states of the cells is conserved via OrthoIntegrate. Other parameters like the NMI-
example is strongly influenced by the cell type labeling [3], which was focused only on main cell type groups in these dataset
subpopulation or mixed cell type population clusters. In other words, subclusters of different cell types were not annotated in
numbers of features that are included in OrthoIntegrate, the clustering might be more diverged, likely by species specific nor
which are not included in the other databases. Therefore, the more divergent clustering, due the increased number of feature
the broad cell type labeling might explain the slightly reduced NMI scores. “

- [ ] 3. The data analysis needs to be expanded further if there are findings with potential biological significance. For example
25, we observe a group of genes showing increased expression in human FBs, and we also identify a set of genes that are ne

human ECs.' However, there is no functional analysis, such as GO or KEGG pathway enrichment analysis, conducted to interp
findings.

We thank the reviewer for the valuable input. To expand the biological significance of the data analysis, we performed gene s
are either enriched in humans, mice or genes that are commonly regulated, in all other cell types. Furthermore, we plotted en
As all these analysis would exceed the scope of the supplementary figure, we uploaded them to the paper specific github acco
(https://github.com/MarianoRuzJurado/RuzJurado_et_al_2023/tree/main/Expanded_Analysis_Figures). Additionally, we perfo
that were regulated in the fibroblast cluster 25 and endothelial cell cluster 28. The results were incorporated in Figure 4B and
accordingly.

- [ ] 4. The discussion section is confusing. The authors should clarify whether the paper is primarily focused on research met
analysis paper, the authors should conduct additional investigations to include further data analysis. If it is a research metho
the discussion to relate to the algorithm itself.

According to the reviewers suggestions, we restructured the discussion section and added additional paragraphs regarding th
benchmarking of OrthoIntegrate. Additionally we have strengthened the research part by incorporating the benchmarking res
paper.

Minor comments:

MinorThings:
- [ ] 1. The cell number for each sample and each clustered cell type is critical for assessing the reliability of the results; howe
in the paper.

We thank the reviewer for this remark and added all QC statistics to the Supplementary Table 6.

- [ ] 2. As the mouse model is generated through chronic infarction, it raises the question of why very few T/B cell markers a
1F. Is it possible that these cell types are not adequately captured in the mouse samples? In data integration analysis, the au
understanding how species-specific cell types perform, particularly when, for instance, only macrophages are the dominant in

We thank the reviewer for this comment. In single nuclei sequencing of isolated hearts, almost no T-cells or B-cells are found
heart cell Atlas by Litviňuková et.al.; Nature 2020 showed mainly Myeloid cells (see https://www.heartcellatlas.org/v2/global
cell cluster in our dataset, we also could not detect any t- or b-cell clusters (see Reviewer Fig.1 in the GitHub Repository
[https://github.com/MarianoRuzJurado/RuzJurado_et_al_2023/blob/15e208c336b668402aa201cfc45d6433c1479ea6/Review
However, for scientist interest in mouse or human specific immune cell responses through chronic inflammation, we provide c
our github repository (https://github.com/MarianoRuzJurado/RuzJurado_et_al_2023/tree/main/Expanded_Analysis_Figures/I
celltype are analyzed there in detail.

.- [ ] 3. On page 5, clarify "latter ones" in the sentence "Most of the latter ones were long non-coding RNAs with identical gen

We clarified the text in this paragraph and changed the sentence to:
"Most of the 86 matches found by lowercasing were long non coding RNAs with identical gene names"

- [ ] 4. On page 5, correct the reference to Supplementary Figure 4A instead of Supplementary Figure 3A and Supplementary

We changed the text accordingly and added Supp_Fig_4 to the main manuscript as Fig 2.

- [ ] 5. On page 16, replace "regulated genes" with "differentially expressed genes (DEGs)" to accurately represent what the

We changed the text accordingly.

References

1. Brudno M, Malde S, Poliakov A, Do CB, Couronne O, Dubchak I, et al.. Glocal alignment: finding rearrangements during ali[illegible]
1:i54–622003;
2. Tanay A, Sebé-Pedrós A. Evolutionary cell type mapping with single-cell genomics. Trends Genet. 37:919–322021;
3. Song Y, Miao Z, Brazma A, Papatheodorou I. Benchmarking strategies for cross-species integration of single-cell RNA sequ[illegible]
14:64952023;
4. Otero-Garcia M, Xue Y-Q, Shakouri T, Deng Y, Morabito S, Allison T, et al.. Single-soma transcriptomics of tangle-bearing [illegible]
reveals the signatures of tau-associated synaptic dysfunction. bioRxiv.
5. Luecken MD, Büttner M, Chaichoompu K, Danese A, Interlandi M, Mueller MF, et al.. Benchmarking atlas-level data integra[illegible]
Methods. 19:41–502022;
