# Supplementary material for: Improved integration of single-cell transcriptome data demonstrates common and unique signatures of heart failure in mice and humans
Source: Gigascience. 2024 Apr 4;13:giae011. doi: 10.1093/gigascience/giae011 (PMC10993718; doi:10.1093/gigascience/giae011)
Supplement: giae011_Response_to_Reviewer_Comments_Revision_1 [file giae011_response_to_reviewer_comments_revision_1.pdf]

Author's Response To Reviewer Comments

Response to the reviewers:

Again, we would like to thank the reviewers for the remarks and valuable suggestions. And also for the fair evaluation of our scientific work. We addressed all the point in the section below and hope that the paper is then acceptable for publication.

Reviewer 2:
The author's additional analysis is commendable. With the inclusion of new evaluation metrics, the benchmark section now appears relatively comprehensive, and the explanations provided for the reduced NMI score are reasonable. In the results section, the supplementary information on functional enrichment further elucidates the biological functions of fibroblast cluster 25 and endothelial cell cluster 28. There are still some minor suggestions for improvement:

1. The presentation of the biological findings in the discussion section could be more succinct to improve clarity.

We agree with the reviewer and tried to shorten the biological discussion section of the manuscript as much as possible. However, in order to show the advantage of OrthoIntegrate to perform a single cell side-by-side comparison of from different species, we think that it is crucial to at least discuss one example for human or mouse specific pathways/genes and commonly regulated genes. Thereby we can exemplify the usability of OrthoIntegrate and show potential research targets for other researchers.

2. There is a lack of discussion on the impact of the numerous lncRNAs generated by OrthoIntegrate. This topic requires further exploration and elaboration.

According to the suggestions we added the following paragraph, to the discussion section of the paper:

“Due to the increased numbers of features that are included in OrthoIntegrate, the clustering might be more diverged, likely by species specific non-coding RNAs or other features, which are not included in the other databases. Therefore, the more divergent clustering, due the increased number of features in OrthoIntegrate combined with the broad cell type labeling might explain the slightly reduced NMI scores. However, since various publications have shown that long-non-coding RNAs have important regulatory roles in the heart [42–44], we think that these additional non-coding RNA’s are an important resource to study species specific responses to different disease condition, especially in the field of heart failure.”

3. Reorganize the paragraphs for "Single cell pre-processing" and "Study samples" to clarify the source of the data used in the article. Emphasize the data generated by authors (E-MTAB-13264) and provide details on the single-cell sequencing process (not only the raw data pre-processing).

We thank the reviewer for this remark. Accordingly we reorganized the paragraphs and added the citation which contains the exact protocol on how the nuclei isolation was done and how RNA was processed and sequenced.
We added the following paragraph to the paper:

"Nuclear isolation steps and single-nucleus RNA-sequencing library preparation were conducted as described in Nicin et al.; NCVR 2022 (Nicin et al. 2022). “
