# Supplementary material for: Improved integration of single-cell transcriptome data demonstrates common and unique signatures of heart failure in mice and humans
Source: Gigascience. 2024 Apr 4;13:giae011. doi: 10.1093/gigascience/giae011 (PMC10993718; doi:10.1093/gigascience/giae011)
Supplement: giae011_Reviewer_1_Report_Original_Submission — Ruoyan Li -- 9/3/2023 Reviewed [file giae011_reviewer_1_report_original_submission.pdf]

Reviewer Report

Title: Improved integration of single cell transcriptome data demonstrates common and unique signatures of heart failure in mice and humans

Version: Original Submission **Date:** 9/3/2023

Reviewer name: Ruoyan Li

Reviewer Comments to Author:

In the manuscript entitled 'Improved integration of single cell transcriptome data demonstrates common and unique signatures of heart failure in mice and humans', the authors developed a pipeline (OrthoIntegrate) to assign gene orthologs across species and integrate cross-species single-cell RNA-seq data based on Seurat workflows. The authors further compared OrthoIntegrate to other orthologue databases and tools methods and highlighted a better performance of their method. To illustrate the potential applications of OrthoIntegrate, the authors integrated single-cell/single-nuclei RNA-seq data from cardiac tissue of heart failure patients with reduced ejection fraction (HFrEF) and a mouse model mimicking HFrEF using the pipeline. This revealed commonly regulated genes in the disease condition between species (i.e., genes related to cardiomyocyte energy metabolism) and species-specifically regulated genes (i.e., angiogenesis-related genes in humans).
Overall, this is a well-designed study with the development of a useful cross-species single-cell data integration pipeline whose applications have been showcased in the context of heart failure (to me it is more like an improved orthologue assignment method)
A few points need to be addressed before publishing
1. The authors utilized the Needleman-Wunsch algorithm to generate one-to-one orthologs between human genes and mouse genes. What is the advantage of using this algorithm compared to other algorithms i.e., SAMap uses BLAST?
2. The authors have shown the application of OrthoIntegrate in the context of heart failure between mice and humans. Could the authors include at least one more example of using OrthoIntegrate in other disease conditions or between other species to show the versatility of OrthoIntegrate?
3. To assess the quality of clustering after integration, the authors calculated silhouette coefficients/scores and found that integration by OrthoIntegrate resulted in an improved clustering performance. Could the authors include more benchmarking metrics to assess the performance of OrthoIntegrate compared to other methods? The authors could consider metrics like the species mixing score used by BENGAL (Song et al., 2022, biorxiv; https://github.com/Functional-Genomics/BENGAL)
4. Miscalling of figures: silhouette coefficients are shown in Supp_Fig_4 rather than Suppl_Fig_3.
5. Some information on the used datasets in the manuscript has been shown in supplementary table 1, but it's still a bit confusing, for example, where the mouse and human HFrEF datasets come from. I am not exactly sure, but I presume HFrEF datasets are from E-MTAB-13264? This information should be described more explicitly in the method section.

Methods

Are the methods appropriate to the aims of the study, are they well described, and are necessary controls included? Choose an item.

Conclusions

Are the conclusions adequately supported by the data shown? Choose an item.

Reporting Standards

Does the manuscript adhere to the journal's guidelines on minimum standards of reporting? Choose an item.

Choose an item.

Statistics

Are you able to assess all statistics in the manuscript, including the appropriateness of statistical tests used? Choose an item.

Quality of Written English

Please indicate the quality of language in the manuscript: Choose an item.

Declaration of Competing Interests

Please complete a declaration of competing interests, considering the following questions:

- Have you in the past five years received reimbursements, fees, funding, or salary from an organisation that may in any way gain or lose financially from the publication of this manuscript, either now or in the future?
- Do you hold any stocks or shares in an organisation that may in any way gain or lose financially from the publication of this manuscript, either now or in the future?
- Do you hold or are you currently applying for any patents relating to the content of the manuscript?
- Have you received reimbursements, fees, funding, or salary from an organization that holds or has applied for patents relating to the content of the manuscript?
- Do you have any other financial competing interests?
- Do you have any non-financial competing interests in relation to this paper?

If you can answer no to all of the above, write 'I declare that I have no competing interests' below. If your reply is yes to any, please give details below.

I declare that I have no competing interests

I agree to the open peer review policy of the journal. I understand that my name will be included on my report to the authors and, if the manuscript is accepted for publication, my named report including any attachments I upload will be posted on the website along with the authors' responses. I agree for my report to be made available under an Open Access Creative Commons CC-BY license (http://creativecommons.org/licenses/by/4.0/). I understand that any comments which I do not wish to be included in my named report can be included as confidential comments to the editors, which will not be published.

Choose an item.

To further support our reviewers, we have joined with Publons, where you can gain additional credit to further highlight your hard work (see: https://publons.com/journal/530/gigascience). On publication of this paper, your review will be automatically added to Publons, you can then choose whether or not to claim your Publons credit. I understand this statement.

Yes Choose an item.
