# Supplementary material for: Improved integration of single-cell transcriptome data demonstrates common and unique signatures of heart failure in mice and humans
Source: Gigascience. 2024 Apr 4;13:giae011. doi: 10.1093/gigascience/giae011 (PMC10993718; doi:10.1093/gigascience/giae011)
Supplement: giae011_Reviewer_2_Report_Original_Submission — Yinqi Bai -- 10/16/2023 Reviewed [file giae011_reviewer_2_report_original_submission.pdf]

Reviewer Report

Title: Improved integration of single cell transcriptome data demonstrates common and unique signatures of heart failure in mice and humans

Version: Original Submission **Date:** 10/16/2023

Reviewer name: Yinqi Bai

Reviewer Comments to Author:

Jurado et al. reported a pipeline designed to optimize the detection of orthologous genes and utilized it to enhance the integration of cross-species single-cell RNA sequencing (scRNA-seq) data. They demonstrated the effectiveness of this pipeline by comparing shared and distinct regulatory pathways between human HFrEF (Heart Failure with Reduced Ejection Fraction) patients and the corresponding mouse model. The work provided reliable results that emphasize the importance of exercising caution when using mouse models to study disease mechanisms. However, many important factors should be critically thought about and benchmarked.

Here are a few major issues:

1. Ortholog identification has long been a critical and essential step for many comparative, evolutionary, and functional genomic analyses. To evaluate the performance of an orthology inference method, there are some gold standards available for benchmark testing, such as the Quest Orthology Benchmark Service (https://orthology.benchmarkservice.org). Whether OrthoIntegrate outperforms other methods should be comprehensively benchmarked on diverse datasets and metrics, rather than relying solely on the silhouette coefficient score from a heart single-cell RNA sequencing (scRNA-seq) dataset.
2. According to the authors' integration pipeline, both human and mouse scRNA-seq data are individually clustered to assign cell type labels and are then further integrated with orthologous genes for clustering to assign new labels. How do the labels for each cell and each cell type change before and after the integration approach? Does cell type assignment become more reasonable after the integration? The authors should demonstrate that the selection of orthologous genes for clustering improves the accuracy of cell type assignment. The silhouette coefficient score is not a direct metric for assessing accuracy, as it can be influenced by biological factors. For example, in Supplementary Table 3, the silhouette scores of mouse-HFrEF samples generated by Paranoid and OMA are consistently higher than those by OrthoIntegrate, which is opposite to the control groups and human-HFrEF samples.
3. The data analysis needs to be expanded further if there are findings with potential biological significance. For example, the authors mentioned, 'In cluster 25, we observe a group of genes showing increased expression in human FBs, and we also identify a set of genes that are negatively regulated in cluster 28 in human ECs.' However, there is no functional analysis, such as GO or KEGG pathway enrichment analysis, conducted to interpret the data and validate these findings.
4. The discussion section is confusing. The authors should clarify whether the paper is primarily focused on research methods or data analysis. If it is a data analysis paper, the authors should conduct additional investigations to include further data analysis. If it is a research method paper, the authors should extend the discussion to relate to the algorithm itself.

Minor comments:

1. The cell number for each sample and each clustered cell type is critical for assessing the reliability of the results; however, this information is not provided in the paper.
2. As the mouse model is generated through chronic infarction, it raises the question of why very few T/B cell markers are found in immune cells in Figure 1F. Is it possible that these cell types are not adequately captured in the mouse samples? In data integration analysis, the audience may be more interested in understanding how species-specific cell types perform, particularly when, for instance, only macrophages are the dominant immune cells found in human samples.
3. On page 5, clarify "latter ones" in the sentence "Most of the latter ones were long non-coding RNAs with identical gene names."
4. On page 5, correct the reference to Supplementary Figure 4A instead of Supplementary Figure 3A and Supplementary Table 3.
5. On page 16, replace "regulated genes" with "differentially expressed genes (DEGs)" to accurately represent what the authors referred.

Methods

Are the methods appropriate to the aims of the study, are they well described, and are necessary controls included? Choose an item.

Conclusions

Are the conclusions adequately supported by the data shown? Choose an item.

Reporting Standards

Does the manuscript adhere to the journal's guidelines on minimum standards of reporting? Choose an item.

Choose an item.

Statistics

Are you able to assess all statistics in the manuscript, including the appropriateness of statistical tests used? Choose an item.

Quality of Written English

Please indicate the quality of language in the manuscript: Choose an item.

Declaration of Competing Interests

Please complete a declaration of competing interests, considering the following questions:

- Have you in the past five years received reimbursements, fees, funding, or salary from an organisation that may in any way gain or lose financially from the publication of this manuscript, either now or in the future?
- Do you hold any stocks or shares in an organisation that may in any way gain or lose financially from the publication of this manuscript, either now or in the future?
- Do you hold or are you currently applying for any patents relating to the content of the manuscript?
- Have you received reimbursements, fees, funding, or salary from an organization that holds or has applied for patents relating to the content of the manuscript?
- Do you have any other financial competing interests?
- Do you have any non-financial competing interests in relation to this paper?

If you can answer no to all of the above, write 'I declare that I have no competing interests' below. If your reply is yes to any, please give details below.

I declare that I have no competing interests.

I agree to the open peer review policy of the journal. I understand that my name will be included on my report to the authors and, if the manuscript is accepted for publication, my named report including any attachments I upload will be posted on the website along with the authors' responses. I agree for my report to be made available under an Open Access Creative Commons CC-BY license (http://creativecommons.org/licenses/by/4.0/). I understand that any comments which I do not wish to be included in my named report can be included as confidential comments to the editors, which will not be published.

Choose an item.

To further support our reviewers, we have joined with Publons, where you can gain additional credit to further highlight your hard work (see: https://publons.com/journal/530/gigascience). On publication of this paper, your review will be automatically added to Publons, you can then choose whether or not to claim your Publons credit. I understand this statement.

Yes Choose an item.
